# Supplementary figures and images for: “If I don’t take my treatment, I will die and who will take care of my child?”: An investigation into an inclusive community-led approach to addressing the barriers to HIV treatment adherence by postpartum women living with HIV
Source: PLoS One. 2023 Apr 20;18(4):e0271294. doi: 10.1371/journal.pone.0271294 (PMC10118130; doi:10.1371/journal.pone.0271294)

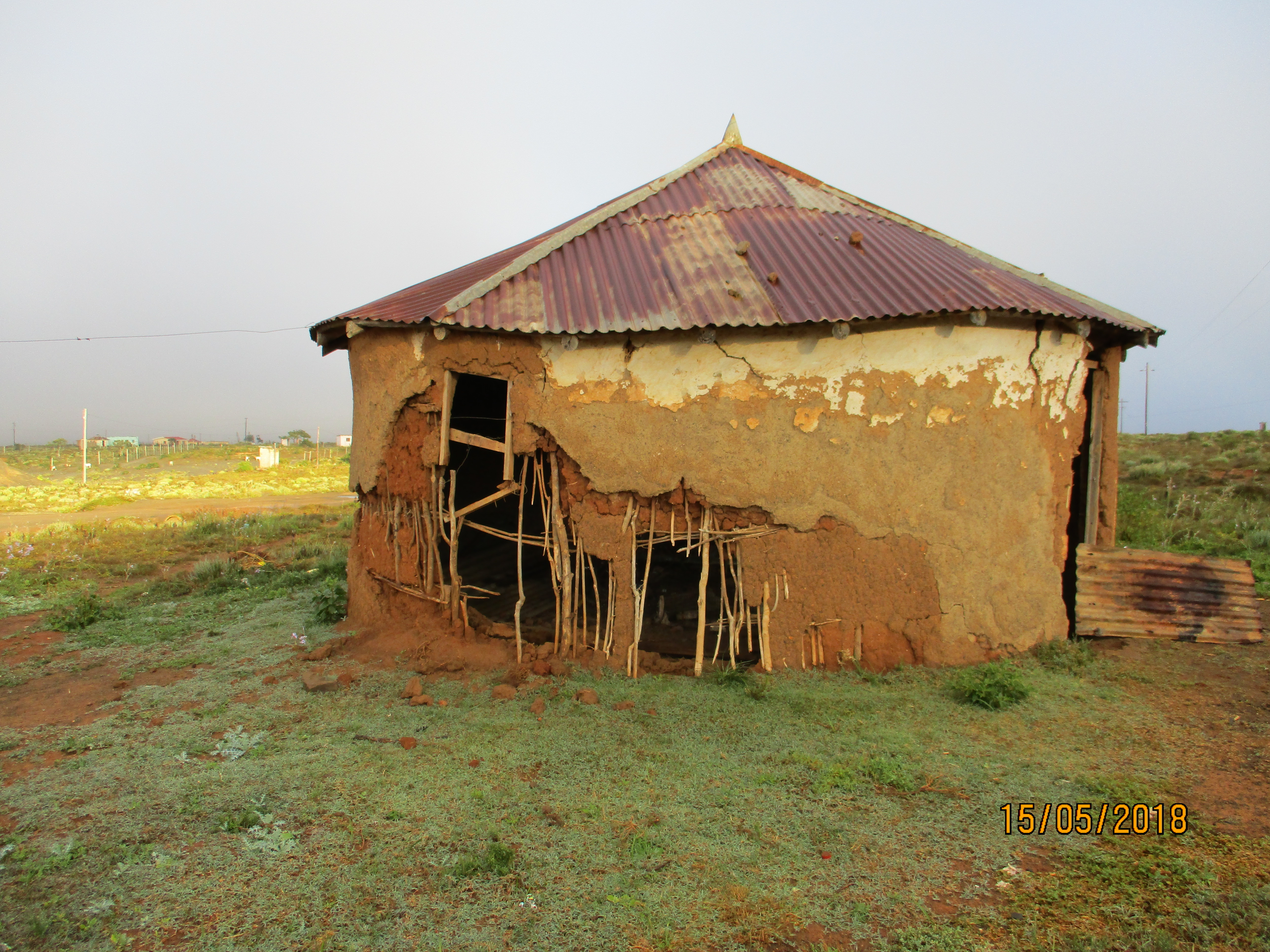

Supplement: S1 File — (ZIP) [file pone.0271294.s001.zip › 14-18 3rd.JPG]

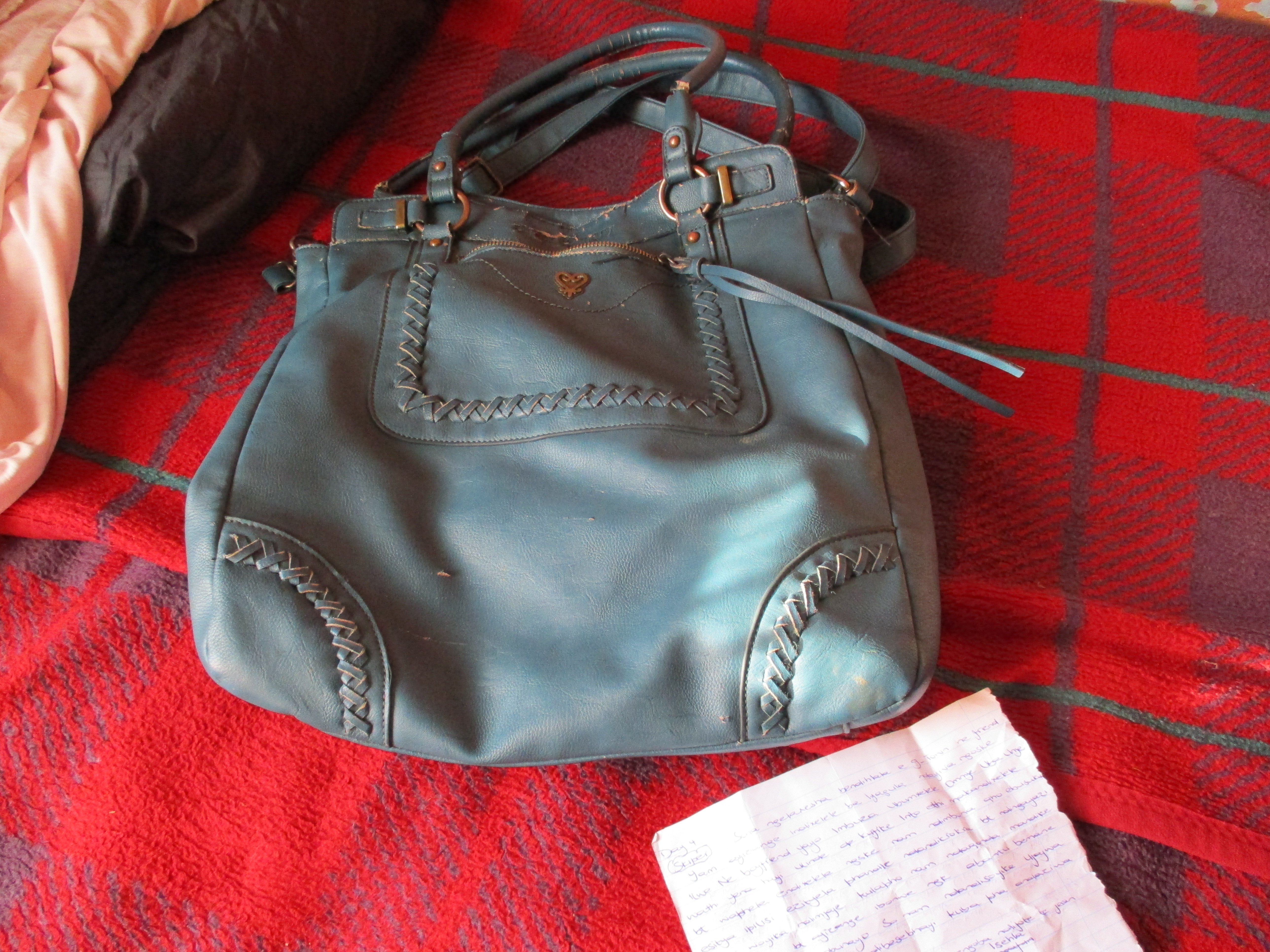

Supplement: S2 File — (ZIP) [file pone.0271294.s002.zip › 22 -18 4th Photo 3 IMG_0018.JPG]

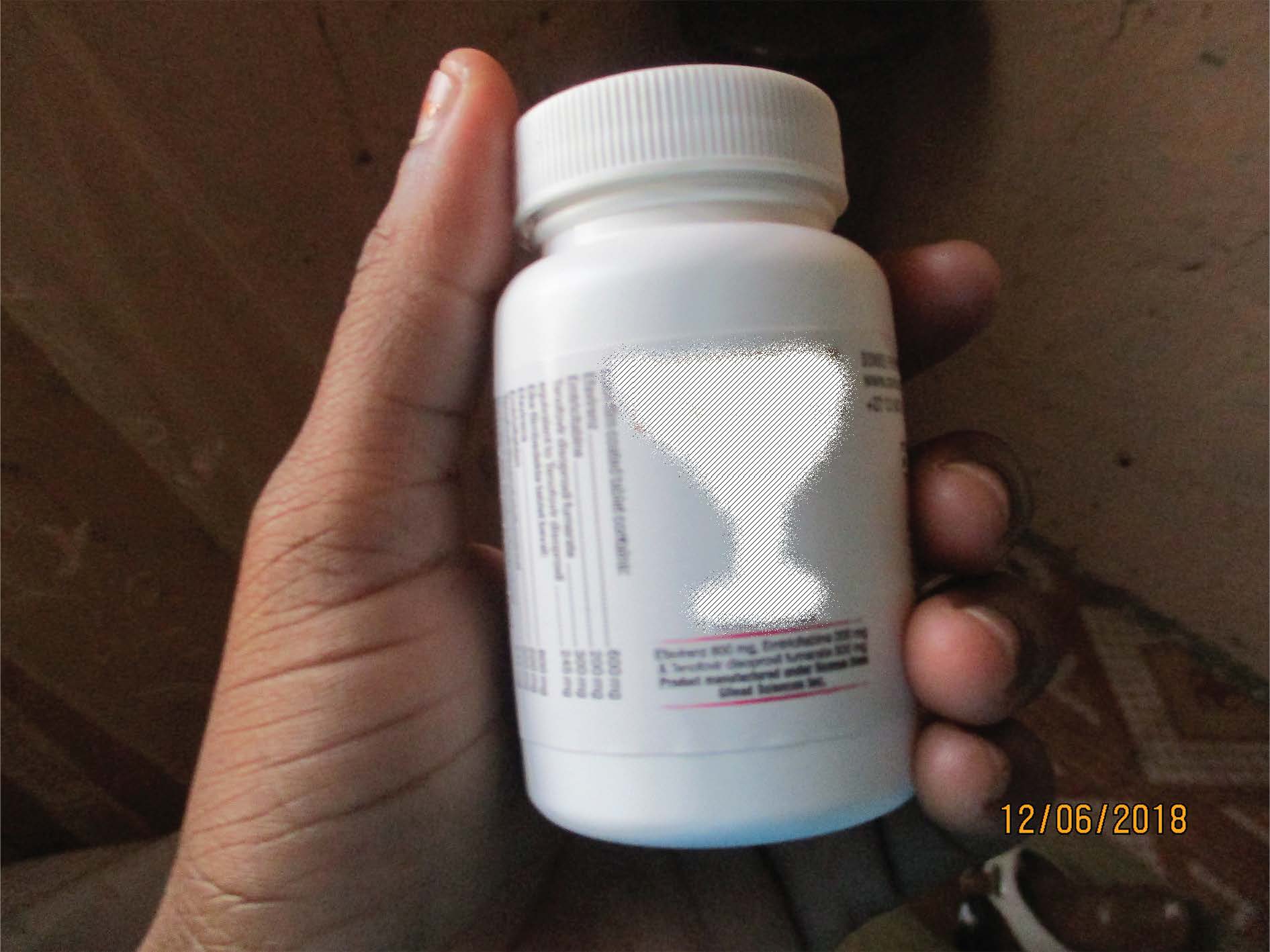

Supplement: S2 File — (ZIP) [file pone.0271294.s002.zip › 22-18 4th Photo 4 IMG_0040.jpg]

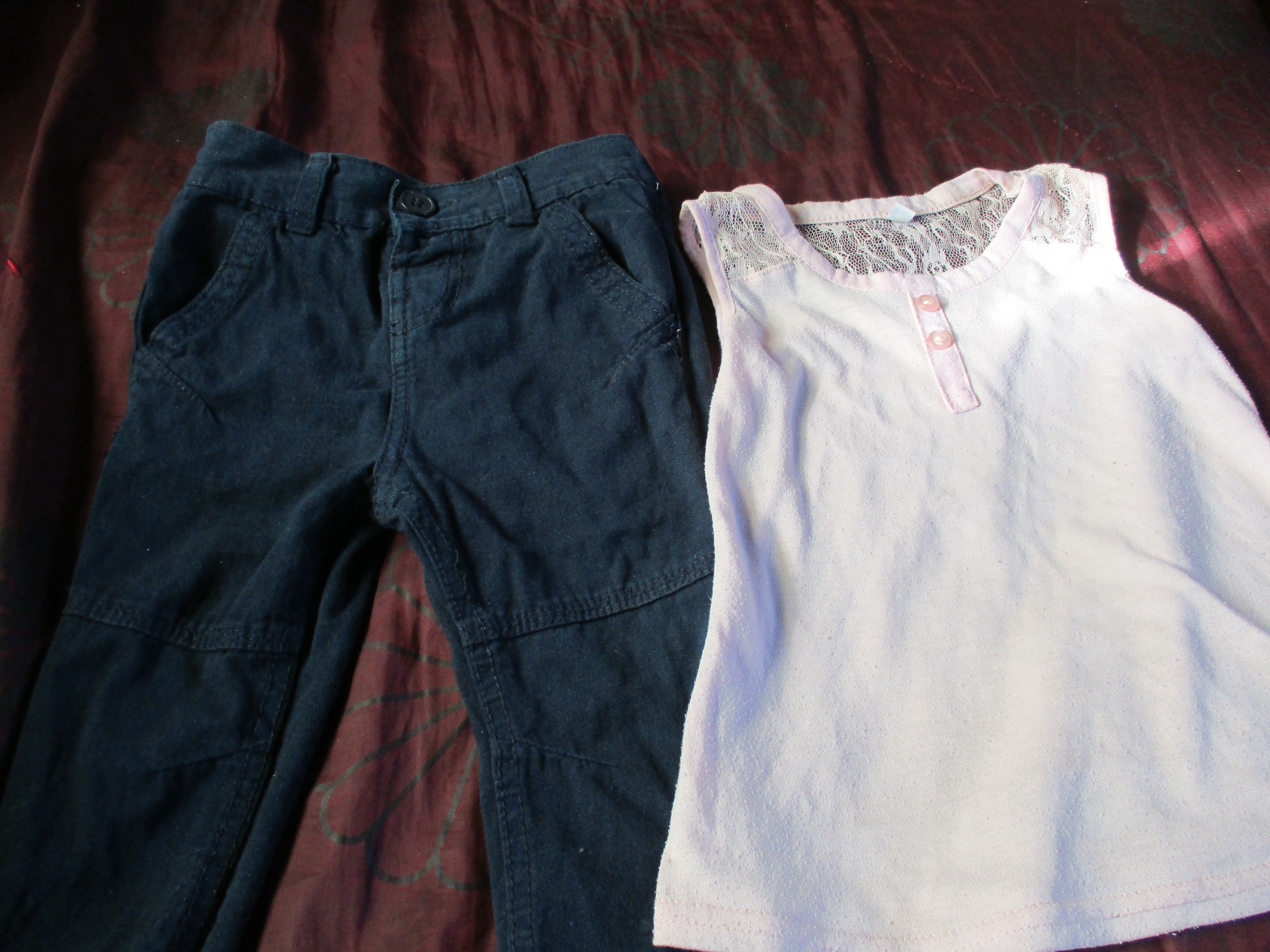

Supplement: S3 File — (ZIP) [file pone.0271294.s003.zip › 24-18 4th Photo 5 IMG_0035.JPG]

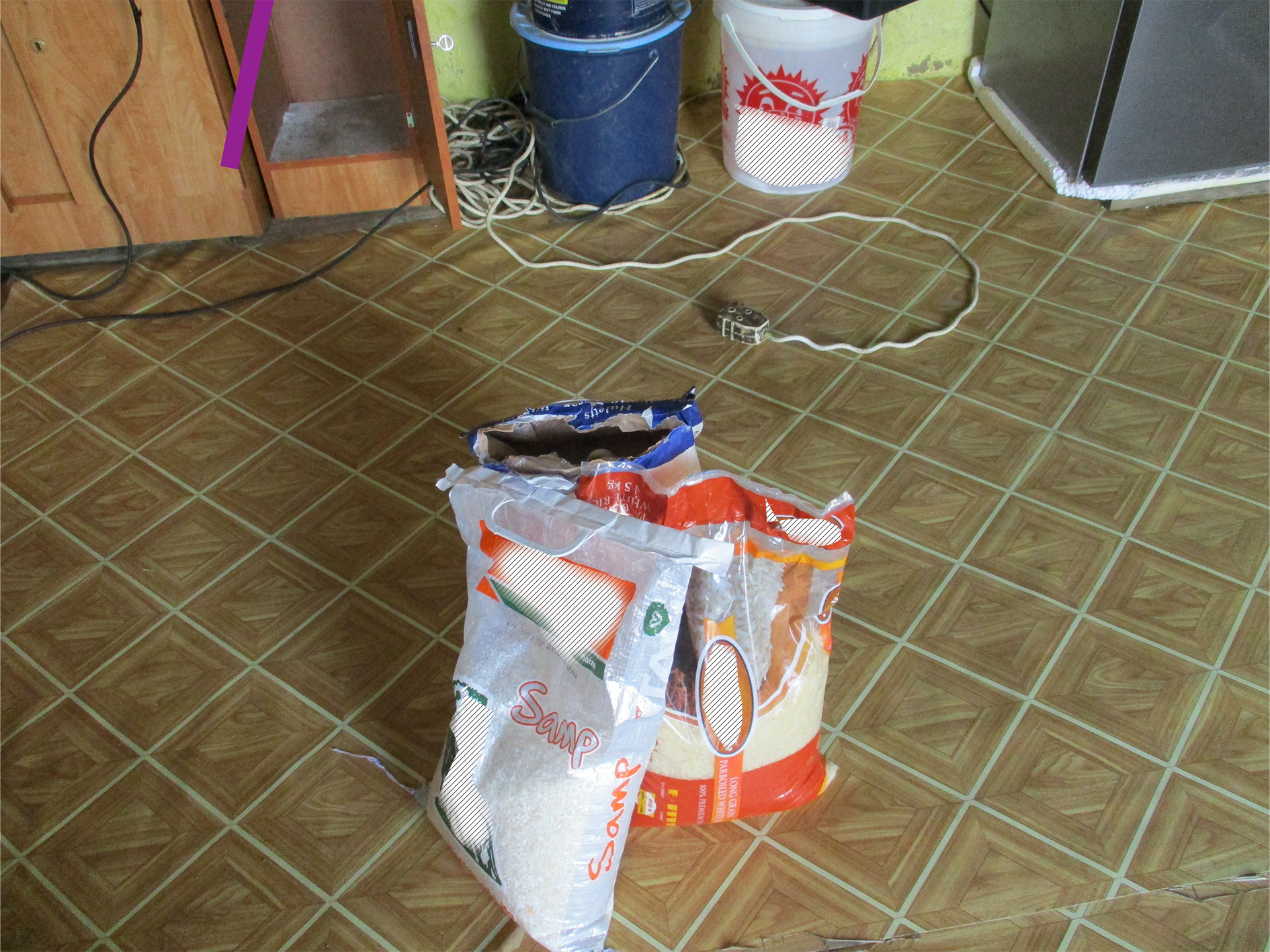

Supplement: S4 File — (ZIP) [file pone.0271294.s004.zip › 32-18 2nd Photo 3 IMG_0040.jpg]

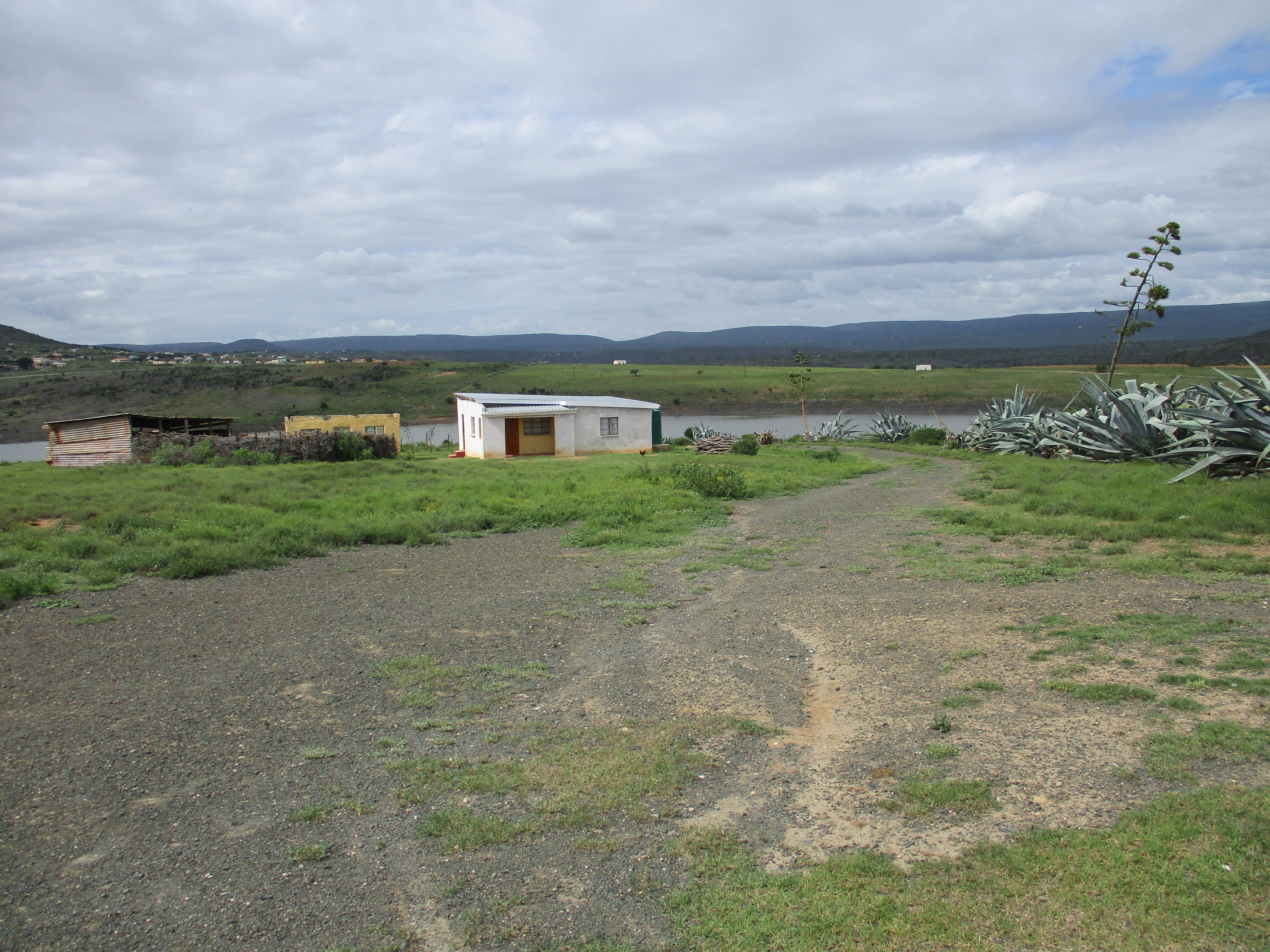

Supplement: S4 File — (ZIP) [file pone.0271294.s004.zip › 32-18 2nd Photo 8 IMG_0049.JPG]

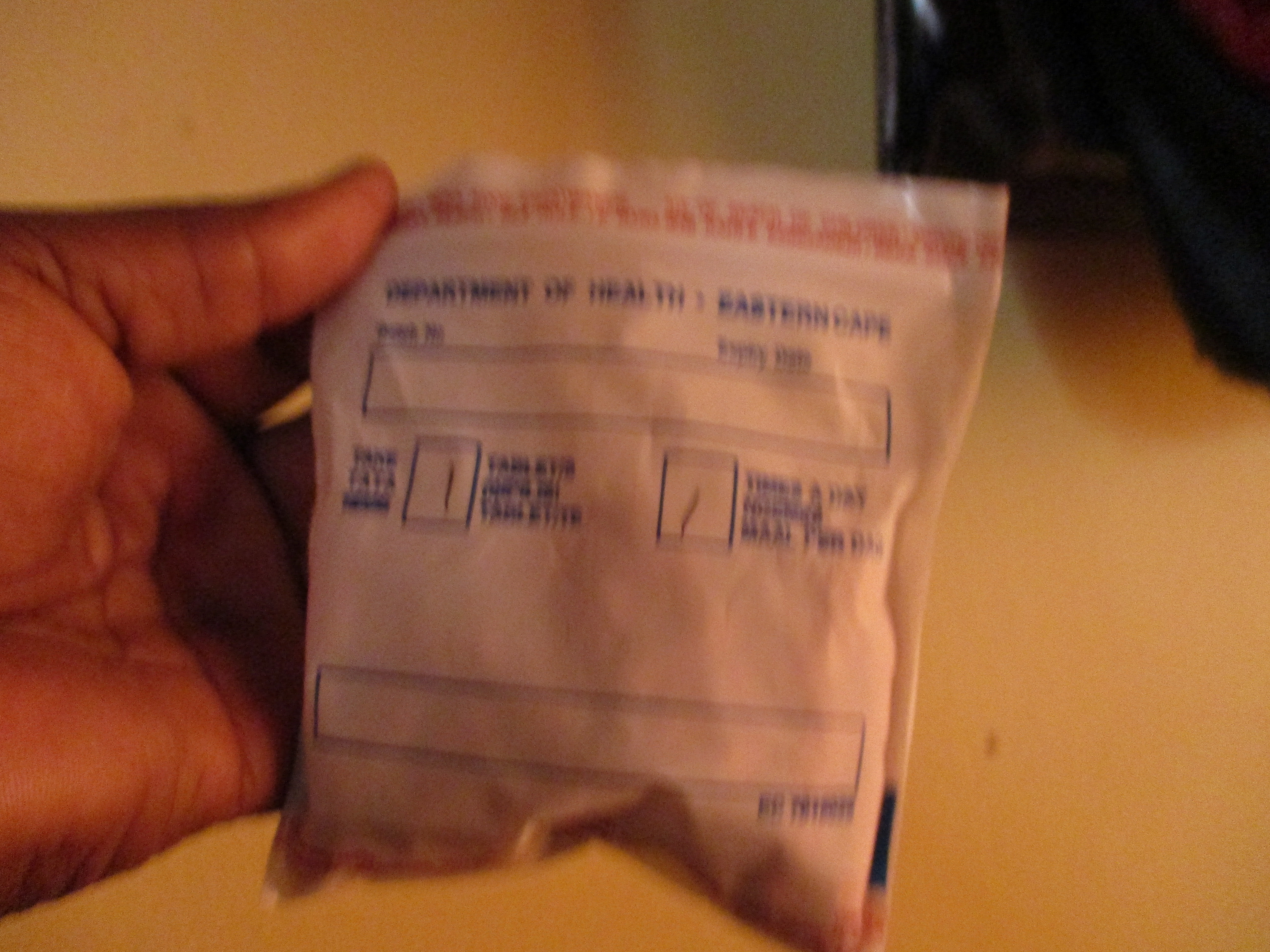

Supplement: S5 File — (ZIP) [file pone.0271294.s005.zip › 34-18 3rd Photo 2 IMG_0074.JPG]

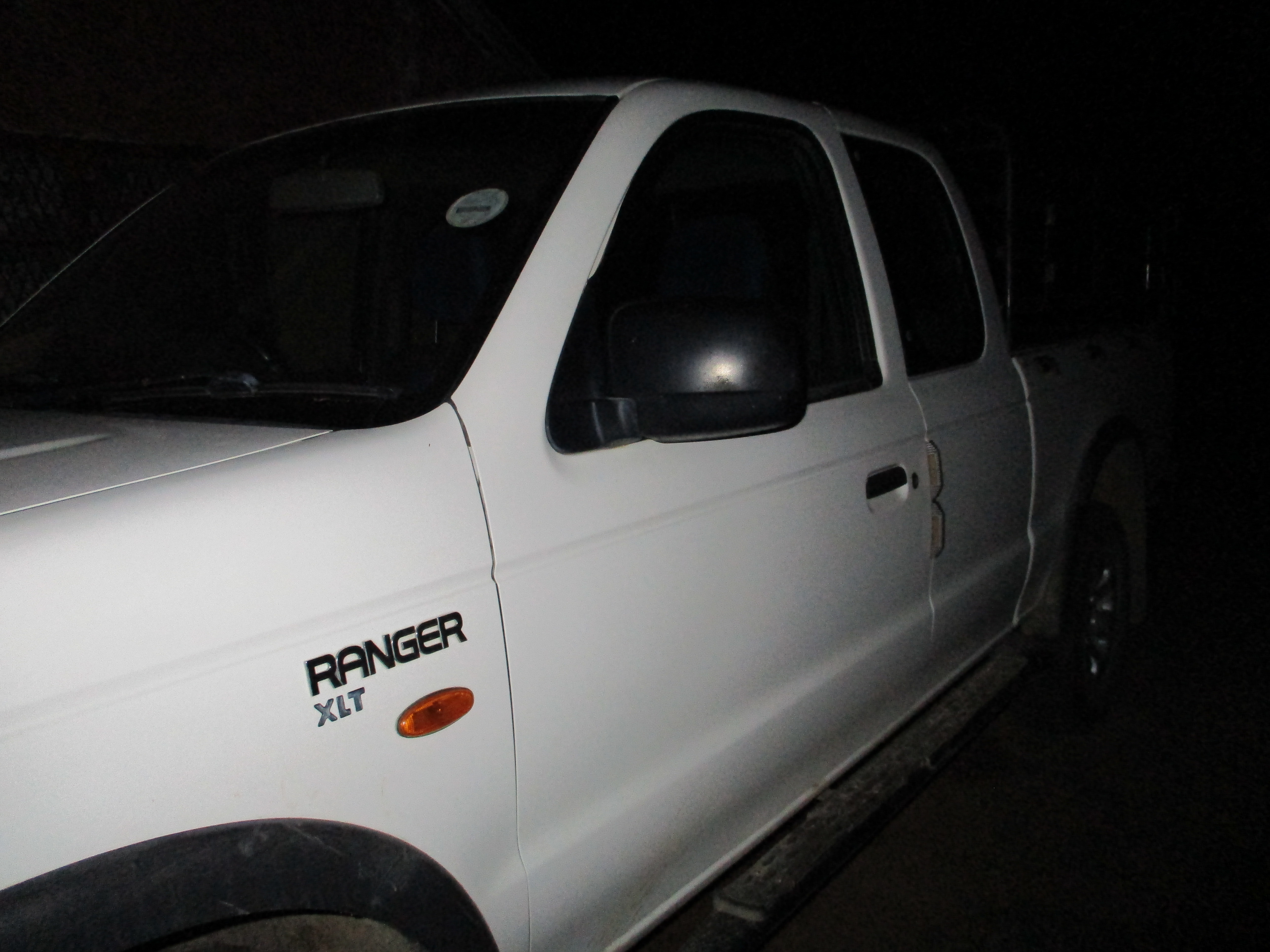

Supplement: S6 File — (ZIP) [file pone.0271294.s006.zip › 42-18 1st Photo 5 IMG_0007.JPG]

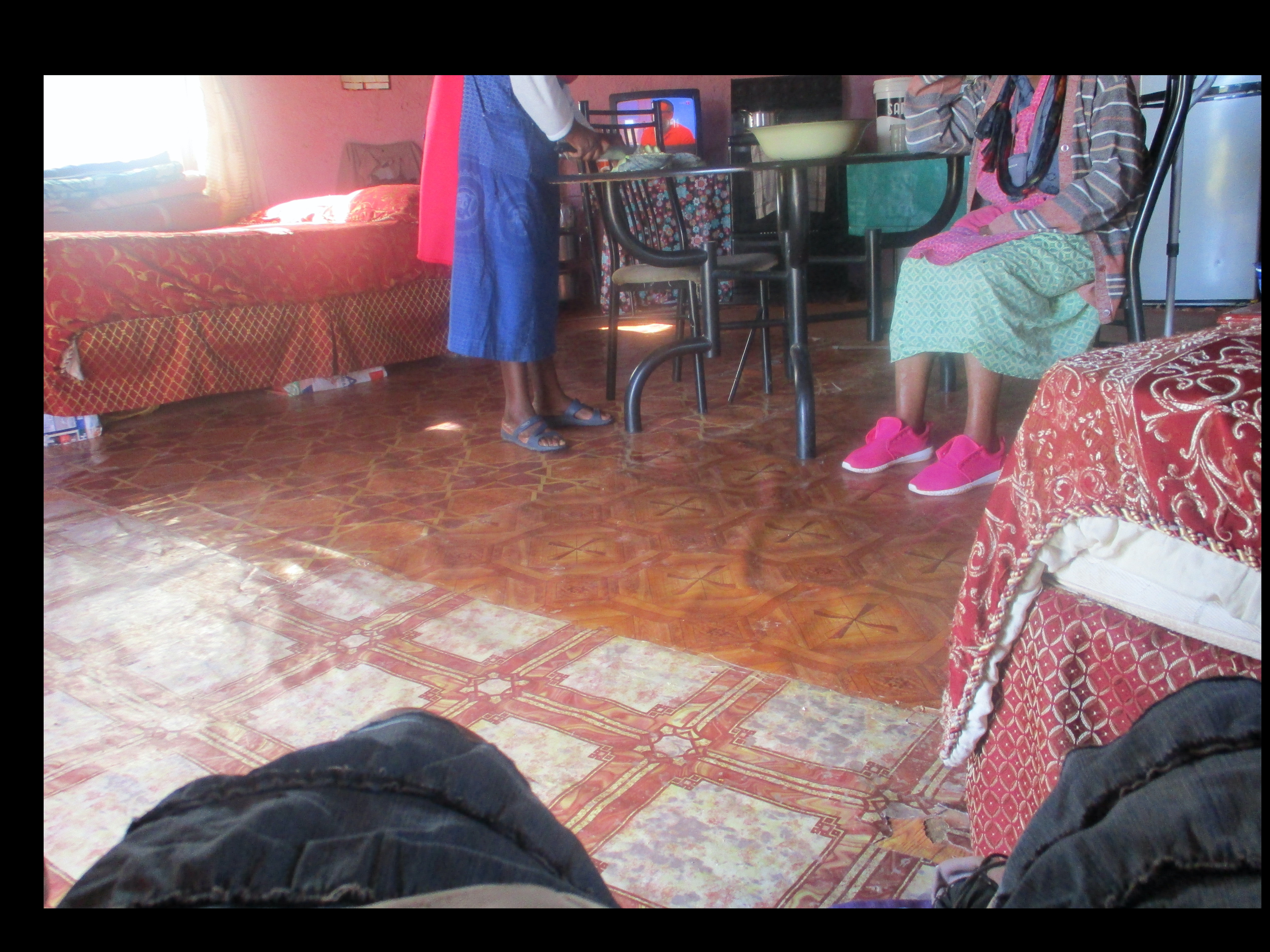

Supplement: S6 File — (ZIP) [file pone.0271294.s006.zip › 42-18 3rd Photo 8 IMG_0077.JPG]

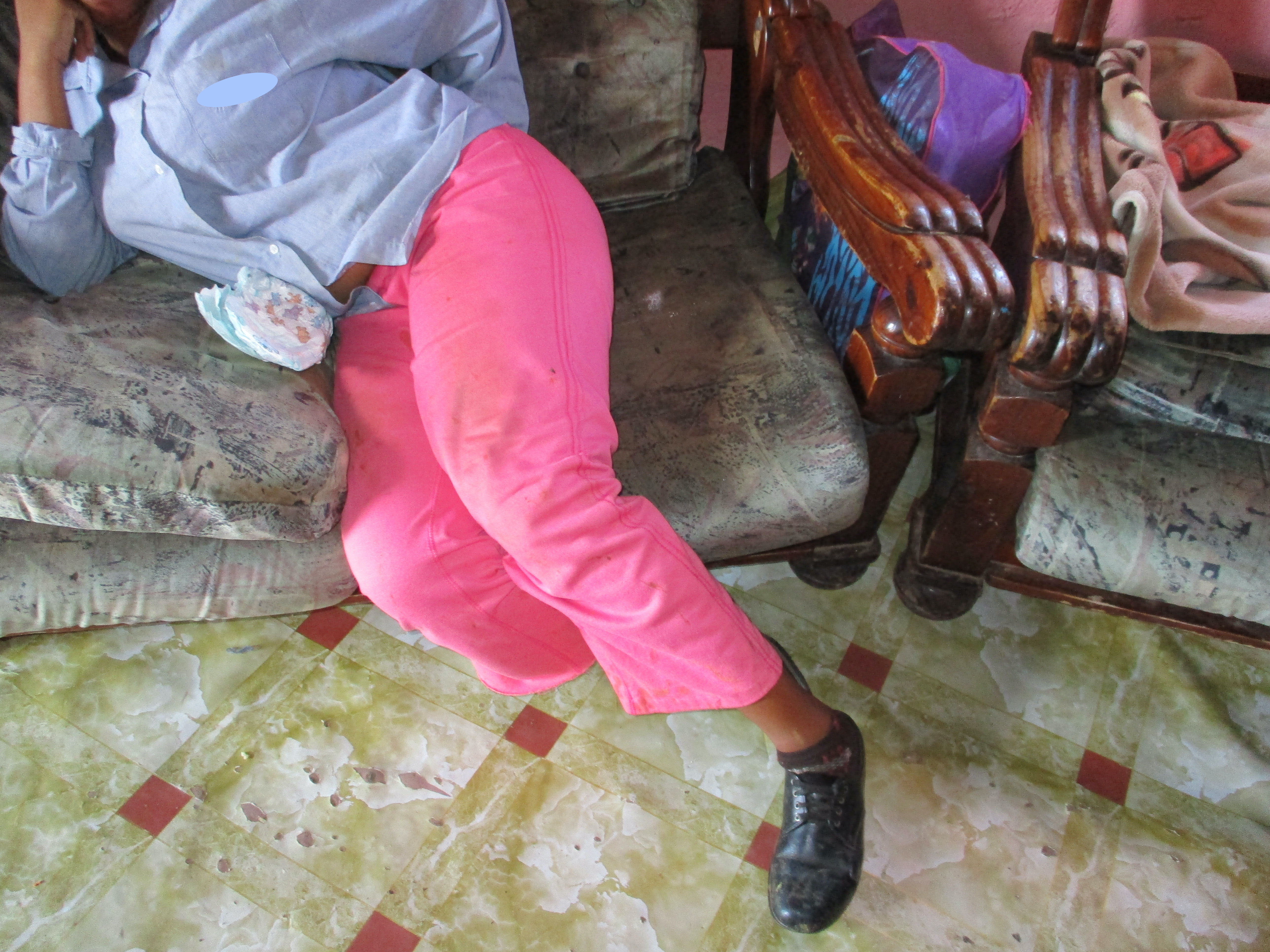

Supplement: S6 File — (ZIP) [file pone.0271294.s006.zip › 42-18 4th Photo 1 IMG_0083.jpg]
