# Supplementary material for: “If I don’t take my treatment, I will die and who will take care of my child?”: An investigation into an inclusive community-led approach to addressing the barriers to HIV treatment adherence by postpartum women living with HIV
Source: PLoS One. 2023 Apr 20;18(4):e0271294. doi: 10.1371/journal.pone.0271294 (PMC10118130; doi:10.1371/journal.pone.0271294)
Supplement: S3 File — (ZIP) [file pone.0271294.s003.zip › 24 18 4th 180621_0038.docx]

1. Good morning today is the 21^st^ of June we are with the participant number 2 4 1 8. It’s her last but unfortunately she took one photo. She is going to talk about it today. Thank you. Ungaqala ke sisi. Iphoto yakho oyifotileyo ufuna uthetha ngantoni

Good morning today is the 21^st^ of June we are with the participant number 2,4,1,8. It’s the last day but unfortunately she took one photo. She is going to talk about it today. Thank you. You can start sisi. What do you want to say, about the photograph you have taken?

1. Le foto ndiyifotileyo kuthetha, ekuqhubekekeni kwam neepilisi khange abantwana bam bachaphazeleke. Khange basuleleke kwisifo sam

This photo I have taken it, to show that my children did not get infected because of the fact of taking the treatment. It was me that made it bad for them, I thought it was my fault

1. Ewe

Yes

1. Ndifuna uthetha lo nto kule pic

I want to say that in this picture

1. Okay
2. Yindlela endiye ndaqhubekeka ngayo netreatment yam

By the way I have progressed with my treatment

1. Ewe

Yes

1. Ndiyabulela ekutyeni kwam itreatment khange ndichaphazele abantwana bam

I thankful by taking the treatment I didn’t infect my children

1. So ngamanye amazwi ufuna uthetha ngeebenefits zetreatment zokutya itreatment.

By other words you want to talk about the benefits of the treatment, of taking the treatment

1. um u

yes

1. ungakhe uchaze nje kakuhle ibi…kwakukwenzeka ntoni engqodweni yakho ngokuya wawukhulelwe kodwa uzazi uphila nesifo intsholongwane. Ewe into eyathi yenzeka apha kuwe ngelixesha ukhulelwe.

Can you say clearly it was… what was happening inside your head by the time you were pregnant knowing that you are HIV positive. Yes something happened to you while you were pregnant.

1. Ndandinayo lanto ithi uba hee ndiyazitya ezipilisi

I had that thing that hee I’m consuming these pills

1. Um u

Yes

1. I wonder njengokuba ndizitya na angekhe mhlawumbi ndiphazame ngenye imini ndingazityi kusuleleke umntwana okanye ingaba lomntwana xa ndigqiba ukubeleka uzophuma eklini na. njengokuba ndizixelele ukuba nditya iipili kanti ezipilisi azindicedi kwelinye icala kodwa khange ndiphele mandla ndiye ndaqonda ukuba manditye iipilisi zam. Xa betshekishwa abantwana baklini.

I was wonder while I was consuming them if I can forget and miss one day what would happen, would my child get infected, does he/she is going to up clean. Whilst I’m taking the pills maybe to happen that at the other side are not helping but I didn’t lose hope, I just realized that I must take my medication. When the children were checked, were clean.

1. O! mani

O! shame

1. Ekutyeni kwam iipilisi khange ndiphele amandla. Bendinazo iintanda buzo ngelinye ixesha ubenalanto yokuba O hayi suka kwezipilisi kufuneka ndizityile kukho nezi zokhulelwe zininzi zizondidika qha khange ndiphele mandla kuyo yonke lo nto leyo. Ndiye ndaqhubekeka ndatya iipilisi zam

When I started with the medication I didn’t stop. I had some doubts, other times have that ‘O in these pills there are even these ones of pregnancy, they are too many and they will be annoying me soon but I never stopped through all those doubts. I continued with my pills.

1. Um u

Yes

1. Ndathi ekugqibeleni ndathi ekugqileni ndizokucedakala. Khange ibe lilize le nto le

Then I said at last, I said at last I’m going be saved. I was not a waste of time

1. Um u. So abantwana khange ba…mhlawumbi na ngesinye isigulo mhlawumbi abe weak. Ubafumene benje…benjani

Yes. So the children didn’t…maybe by some other illness be weak. To find them like this and like that

1. Beright

She or he was fine

1. So nakulo wokuqala umntwana ubusowuhleli upositive

So even with the first child you were already positive

1. Ewe

Yes

1. Ubosowuyitya le treatment, okay unangaphi kanene yena?

You were already taking the treatment, okay, how old she or he is?

1. Una5

She or he is 5

1. Una5… O

She is 5… O

1. Khange ndibenamntwana…nangoku andikabi nam mntwana, caba ndiyolala esibhedlela

I never had a child… even now I don’t a child again, to find out I’m going to admitted to the hospital.

1. Um

Yes

1. Okoko ndaphuma esibhedlela ngalamini ndandigqiba ukubeleka andizange ndiphinde ndilale sibhedlela

Since the last day of the delivery I was never again admitted to the hospital again

1. O mani…mh..hayike ziindaba ezimnandi ezo. So ukutya itreatment kuwe kuyakukhuthaza

O those are good news. So to you taking a treatment motivates you

1. Kuyandikhuthaza

It is motivating

1. Ewe…ayikho enye into ke kuba namhlanje iyimini yethu yougqibela. Yiveki yethu yokugqibela ayikho enye into bungayithetha nje. Nangantoni emalunga netreatment okanye neklinikhi amanesi okanye onoompilo nje..obawela ukhe uthethe ngayo. Noba mhlawumbi zizinto ebezingakuphathi kakuhle okanye ebeikuphatha kakuhle

Yes… so don’t have something else since is our last day. This is our last week, you don’t have something else that you would like say. Anything that is associated with the treatment or the clinic, nurses or the social workers…something you would like to talk about. Even if that the things that make you feel unhappy or happy.

1. Into endinothetha…kukukhuthaza ukuba umntu aqhubekeke nokuthatha itreatment yakhe. Ukutya itreatment akulolize andiboni alohlobo akulolize ukutya itreatment kukucedakala kuwe uzokwazi uyokuceda nomnye ongafuni ukutya

Something that I can talk about…it is to encourage that someone must continue with his or her treatment. It is not the waste of time to take your treatment, I don’t see in that way, it s not a waste of time it is to help yourself so that you can go and help those who refuses to take their treatment

1. Um u

Yes

1. Ubona uqina ukhuthaza nalo kwitreatment yakhe

It motivates even this one to take her or his treatment to see yourself strong

1. Ngoku wena apha ekuhlaleni bakhona obakhuthazayo

So there are here in your community that you motivates

1. Bakhona abanye qha umntu askhe apholelwe aqonde ukuba hayi suka uyadlala akutyi treatment wena

There are some but you know someone will say no you are playing you are not taking any treatment

1. Um u

Yes

1. Akudele athi hayi suka wena awuguli

And disapprove you by saying you are not sick

1. Um u

Yes

1. Andazi nokuba xa ugula kufuneka ulale flat na kwabanye abantu. Phofu wonke umntu ugula ehamba.

I don’t know maybe when you are sick you must be bed ridden to known as a sick person. Any way almost is sick though they are still on their feet

1. Um u

Yes

1. Suka apholelwe yinto oyithethayo ube wena ekugqibeleni umceda

Someone just disapprove what you saying while you are helping him or her

1. Mhlawumbi kaloku babona lento ukuba uhappy. Abanye nyani bacinga ukubana heheheeekufuneka ude ubonakale

Maybe they see that you are happy. Truly others think that, heheheeee you must be bed ridden to look like someone ill.

1. Ndadlala

I’m joking

1. Okay ngekliniki ke mhlawumbi ikhona into ongathetha ngayo ngekliniki okanye amanesi asekliniki okanye onompilo ntle chw

Okay don’t you have anything to say about the clinic or the nurses or the socialworks

1. Ikliniki iyayidlala indima yayo emntwini gqithi and naxa mhlawumbi kunokwenzeka ugqube idate yakho ababe bekubuza ukubana bekutheni ukuze ugqube idate bakunika itreatment yakho bakuxelele ukubana ukubana awuzokwazi ukuza ngaladate yikhawulele idate bamhlawumbi uzoba bhizi ngalodate yikhawulele idate ukwenzela ungagqubhi ukutya iitreament zakho

The clinic plays its part in a human, and they even say if you think that you are going to miss your date, you can even come earlier than the desired date, if maybe you are going to be busy, come earlier so that you don’t miss the date of the treatment

1. Um u

Yes

1. Namanesi ke nawo athetha kakuhle nabantu ayakhuthaza

Even the nurses speak well with people, they encourage

1. Um u

Yes

1. And awafuni ungayithathi itreatment yakho

And they don’t want you to miss your treatment

1. Um u

Yes

1. Afuna uyithathe ngoba iluncedo kakhulu

They want you to take it because it is helpful

1. Um u…hayi ke enkosi kakhulu ke sisi siyabulela nangexesha lakho nangona ke besibawela mhlawumbi nokuba kungathiwa ioifoto zininzi because izitori zakho ziyakha and siyafunda into eninzi njengokubana benditshilo ukubana siyafundisana apha, siyakhana, siyazama nokubana iiformation le sikwazi ukuyidibanisa nenye esiyifumana kwabanye sizokwazikangoku ukuba silungise iindlela ezithile esiqhuba ngazo. Eee sibakhuthaza nokuba khuthaza ukubana siyafuna ukukhuthaza…enkosi kakhulu

Yes…thank you very much sisi we are thankful about your time although it was going to be a good thing to hear that your photos are many, because your stories are motivating and we are learning from the things which are like this hence I was saying we are teaching one another here, we are building each other, we are trying to add the information we are getting even from others so we can fix some other ways we are doing things. Motivating them a lot, because that’s the thing we want to do. Thanks a lot.

1. Um u

Yes

1. Siyabulela ngexesha lakho

We are thankful about your time
