# Supplementary material for: “If I don’t take my treatment, I will die and who will take care of my child?”: An investigation into an inclusive community-led approach to addressing the barriers to HIV treatment adherence by postpartum women living with HIV
Source: PLoS One. 2023 Apr 20;18(4):e0271294. doi: 10.1371/journal.pone.0271294 (PMC10118130; doi:10.1371/journal.pone.0271294)
Supplement: S4 File — (ZIP) [file pone.0271294.s004.zip › 32-18 2nd 1226 180314_0017.docx]

Good morning today the date is the 13^th^ of March 2018 and I’m now going to talk about the photos of our participant 3,2,1,8, participant 3,2,1,8 she says she enjoyed taking the photos she didn’t had problems last week. This is the second week of the photos then we can start.

2. Okay, photo number 1. Le, mmm yiplisi, yipakethi yepilisi. Indikhumbuzi i…ngokuya bendinale…itreatment yam yeARVS kuwe kwadevelopa ipressure ndangumntu kangoku otya ipilisi zepressure

Okay, photo number 1. This one, mmm it is a pill. It is a pack of pills. It reminds me a...when I had this...my ARVS treatment, then a high blood pressure developed as well and then I became someone who takes the treatment of high blood pressure.

1. O! khange utsho

O! You never said so

2. Khange nditsho, ziye… zi… ziye… zi...vela ezizinto apha kum as ba kudala ndingumntu osebenzisa itreatment yeARVS kuba…kwakukwenzeke ntoni nini ngoku kuye kwavela khumbula kaloku wawuye wadivelopa ippressure kwacaba uyacinga ndaya ekliniki kwabhaqwa ippresure. Ndatya iipilisi zeppressure…

Wathi uNesi, kwenzeka ntoni kuwe?

Ndathi andiyazi.

Wathi kanti andikufuni ufole namaxhewukazi, yabona ngoku sowukwippresure uzoba neetreatment ezimbini eyeRAVS plus nepressure su..wandicebisa ke wathi kum sukuyicinga into engasoze incedwe nanguNesi…yibaryt awuguli uyagula na ndathi andiguli qha into eyenzekeliyo apha kuwe awugile qha kuphazamisekile andiyifuni le pressure yeepilisi apha kuwe washo noNesi. Yiba rayithi yiba nguwe qha kuphazamiseke igazi.

Ndathi kulungile ke ndizozama ukungayicingi.

Wathi andiyifuni le pilisi kuwe ndifuna le pilisi iyione. Le ipilisi iyione yile yeARVS. Yile ingaze incedwe nagunesi watsho kum. Yitya qha… into esiyenzayo zizama ukuthomalalisa watsho kum so ndifuna uyiyekile le ipilisi yesibini ungafoli namaxhewukazi yibaryt.

Xa ndiphinda ndisiya ekliniki ndathi izothini le nto kum iARVS, iPressure, mmmhh xa ndiphinda ndisiya eklinikhi, ha ha ipressure ayabonakala. Ndazixelela inoba ngoku...inoba ngoku ndizixelele ukuba uthe uNesi ndizoba rythi mandizixelele ukuba ndirythi igazi lam qha liphazamisekile mna ndisengulamntu bendinguye yavela yanyamalala kangoku ipressure so kangoku ndingumntu otya le ARV yodwa mmmh

I never said so, they...they...these things (sicknesses) gradually appear in me. I’ve been this person who was using ARVS because...what happened when it happened what is going, now out of the blue, think back you developed a high blood pressure because it happened that you were stressing too much then I went to the clinic and they saw a high blood pressure. Then I took pills of high blood pressure...

The nurse said, what is happening with you?

I said I don’t know.

And then she said, you know what, I don’t want you to queue with the grannies; you see now you are treated for high blood pressure you are going to have two treatments, the treatment of ARVS plus the treatment of high blood pressure don’t...then she advised me and said ‘don’t think too much about the thing that cannot be reversed even by a nurse. Be alright, you are not sick.’ “Are you sick.” And I said no I’m not sick. ‘The thing is, the thing that had happened to you is “you are not sick” the thing is you are just disturbed. I don’t want this pressure of pills on you’ and the nurse said so as well. Be well, just be you. The thing which had happened, the blood is disturbed. Then I said is all well I will try not to think too much. She continued by insisting ‘I don’t want this pill in you. I want only this one.’ This pill is referring to as the one, is this one of ARV. The one that will never be reversed even by a nurse she said that to me. Just take your treatment...the thing is, we are trying to control your sickness she said to me ‘so I want you to lose the second pill so that you don’t queue with the grannies. Be well.

I said to myself, when again go to clinic what this is going to mean to me it is ARV, Pressure, mmmh, when I went to clinic again, No, no pressure is seen. Then I told myself maybe now...maybe now I’m going to be fine as the nurse had told. I must tell myself that I’m going to be fine it is just my blood is disturbed. I’m still that person I was and the pressure just disappeared. So now I’m just this kind of person who takes ARVS only.

1. Ooh...usakhumbula kwakunini ngokuya wawuthatha iipilisi zepressure?

Ooh! Do you still remember the day you were taking the pills of high blood pressure?

2. ithi...le pilisi yepressure ndiyathathe kunye nalepilisi yeRAV. IRAV iskhe ingathi ndiyiqale 2009

It says...this pill of high blood pressure I have taken it on the very same time with this of ARV

1. O okay

2. ngelixesha iskhe ingathi bendingeka mkeli kakhle kakhle ukuba mna ndiphila nale ntsholongwane...ummh...ndangumntu ocingayo kwazodevelopa ipressure...kwathiwa ucinga ntoni...ndathi Nesi andazi...kwadevelopa ipressure...wathi uyacinga wena...kanti lento...ayi...ayizo..ayizo...ayizolantika...awuzophila qha siyayithomalalisa sukuyicinga ayizokuncedwa nanguNesi. Sucinga into engasoze incedwe nanguNesi. Yibarythi. Ndayibona kangoku i...ipressure yam seyirythi. Ndabe kanti ndohlukana nepressure ndijongana nepilisi eyione

It seems very likely by this time I was still battling to come to terms that I’m living with this disease... ummh... I became this somebody who stresses a lot and then the pressure developed. Then they said ‘what are you stressing about’... then I said ‘nurse I don’t know’... the pressure developed... then she said ‘you, you stresses a lot’... and this...it will... it will never...it will never... it will never do what?... you are not going to be cured... what we are doing, we are just controlling it. Don’t stress too much about the thing, even a nurse won’t be able to reverse. Be well. Now I saw then... my... my pressure was right. And by that way, I parted with the pill of pressure and I was face to face with one pill.

1. so yastotywa le nto yepilisi yepressure

So this thing of the pressure pills was stopped

2. yastotywa...yastotywa kangoku ipilisi yepressure

It was stopped...then pill of pressure was stopped

1 Oh!

2. so le pilisi iye yandikhumbuza ukuba hayi kaloku wawusitya ipressure kunye neARV kumacebiso kaNesi ndangumntu oryth kangoku wathi suyicinga le nto ingasoze incedwe ngunesi.

So this pill reminds me that I was the person who was taking pressure treatment and through the advises of Nurse I became a better person, then she said ‘don’t stress so much about the thing that will never be reversed by a nurse.

1. umm...siyagqitha kangoku

Umm...we are going forth now.

2. siyagqithat kangoku...siya kula...kwiii...ku..

We are going forth now...we are going to that...in this...in...

1. kufoto namba 2

In photo number 2

2. kufoto namba 2...ufoto namba 2. Hayi kengoku sendiyigqibile ndidlulile kweyepressure sendisitya iARVS zam...nding...ndiyazonwabela futhi... qha into eyenzekayo kaloku...andithi singamaXhosa. Ngelinye ixesha ndisemigidini phakathi kwabantu lifike ixesha lam lokutya iipilisi lidlulile

In photo number 2... photo number 2. Yes! By this time now. I have already gone past the treatment of high blood pressure. I’m the only my treatment of ARVS...I’m... I’m enjoying them as well... by the way... this is the thing which is happening...you that we are people of amaXhosa. Sometimes, I’m in the traditional gathering, among the people, the time to take my pills come and go past

1. Ohh

2. liyadlula ke lona ixesha ndiqonde okuba ohhh lidlulile ixesha ndiyakwazi ngelinye ixesha ndifike ndiyity but diyitye le pilisi ndive ba ndiyayitya le pilisi kodwa andiyazi into kuba izokwenzeka emzimbeni wam.

The time goes past, and I become surprised ohh! The time had went past but sometimes arrive at home and take the pill, feel that I’m taking in this pill but clueless of what is going to do inside my body.

1. umm

Yes

2. Izqonde uba ixesaha lidlulile bendipha and ndiyalibona ixesha lifikile ixesha letreatment yam ladlula lona. Ndasendlini kangoku ndathi ha a ifane sendisenza nje ndizozibona apha emzimbeni wam izokwenza kanjani na yona

As you may understand my had went past and I’ve noticed the time to take the treatment had arrived and went past. And I was at home, then I said any way for the sake of just doing it, I must take my treatment I will it then what is going to do inside my body.

1. so xa uhambile mhlawumbi uye emgidini kanjalo awuyiphathi ipilisi udla ngokuba uyishiye apha

So when you are going to attend the traditional gathering you don’t carry the pills with, something like that.

2. mhlawumbi umgidi u kwaround apha ekuhlaleni

Maybe the traditional gathering is around here in my area

1. kodwa qha

But why

2. ndizixelele ndabendizixelele ndizaphinda ndibuye before ixesha leepilisi kwenzeke kumnandi, ndingakwazi ubuya ngexesha leepilisi kubemnandi libe kanti ixesha lidlule kodwa xa ndise ndlini ndifane ndiye epesini yam ndithathe ipilisi nditye ndilale

Sometimes I tell myself that I’m going to leave the traditional gathering early before the time to take my pills reaches, then it happens that I’m overwhelmed by the excitement of the gathering just then to notice that the time to take my medication had went past but I’m at home, for the sake of my routine I go to my purse, take my pills and go to sleep.

1. Okay

2. but libe lingadlulanga nam ndenze ukuba lingadluli kakhulu ukwenzela ukuba umonakalo ungenzeki kakhulu ndithathe iipilisi nditye

But the time had not went past, because I made it a point that the time does go past too much to prevent the occorance of a great disaster and then take the pills

1. um um...siyagqitha kangoku

Yes...we are going forward then

2. sidlule

Yes we can go forward

1. ngufoto namba 3

It is photo number 3

2. Foto namba 3. Leya ngufoto namba 3. Ezba ke ba ndingumntu ongaphangeliyo apha kufoto namba 3 le ibubugrocerantyana. Ezba ezipilisi zityiwa kunye nokutya mna ndingumntu ohleli nje akanakutya ngelintye ixesha kufuneka ndihluthi ndijonge iipilisi zam ndivele ndizigqume ndingayityi ngoba ngelinye ixesha andinakutya hayi ingaskhe nje kubekho umntu ndifumane noba yihamper ethile ndibengumntu nje ofumana ihamper ethile ndazi ukuba ngexesha elithile likhona ithemba ndiyayifumana igrowusari ndipheke ukuze ndizotya le pilisi

Photo number 3. That one is photo number 3. Because I’m the unemployed person, this was a little bit of a grocery in photo number 3. Because these pills are taken after the food, you must be full and I’m someone who is always without food. I look at my pills then just cover them with something and not take them because sometimes I don’t have food. O! If there can be someone, even be sponsored with a hamper, just be someone who gets a kind of a hamper so that I can know sometimes there is hope. I will get a grocery and cook. Then take my pills.

1. so apha uzama uthini apha malunga nepilisi nokungabi nakutya ?

So here what are trying to say with this issue of pills and of having no food?

2. apha kulefoto ndizama ukuthi eyona nto indipenekisayo kungabi nakutya ngamandla. Eye indityhafise ngelinye ndiye...lifike ixesha sibe isusu sam siempty ndivele ndigqume ndiyayazi ndithi ndiyayazi le nto ndiyenzayo iwrongo but isusu sam sona siempty

Here in this photo I’m trying to make this point. The only thing that makes me panic a lot and makes me despair, is the fact of not having food...the time to take the pills comes but then my stomach is empty. I just covered them with something. I know what I’m doing is wrong but it is just, my stomach is empty.

1. Uhhh!

2. kufuneka ndibe nokutya. Ukutya andinakongo

I must have food. I don’t have the food.

1. okay sigqithe kangoku

Okay let’s go forward then

2. siyedlula kangoku

We are forth then

1. foto namba 4

Phot number 4

2. foto namba 4 ezi...

Photo number 4 these...

1. ungaqhubeka

You may proceed

2. Okay ezi zipilisi zostopa iTB. Ifoto namba 4. Zipilisi sosistopa iTB. Zona ndiziqale kulonyaka uphelileyo ngoAgasti. Ndizozigqiba kulonyaka ngoAgasti still. Um um zipilisi zositopa iTB ezi ndiye ndazilibala phaya ngaphambili kuthi kanene ndiyazisebanzisa

Okay. These are pills to stop the TB. Photo number 4. Are pills to stop TB. I have started taking them as from last year by August. I will still finish the course of taking them in this year by August. Yes these pills are to stop the TB I forgot to talk about earlier that I take them as well.

1. ubuyiqale nini itreatment yeTB

When did you start with TB treatment?

2. ITreatment yostopa iTB kuba ibinga...ibingafunyanwa iTB iqale

It is the treatment of TB, to because...TB was untraceable at first

1. oh ibingafunyanwa

Oh! It was untraceable

2. yeyopreventa iTB

It is used (the pill) to prevent TB

1. yeyopreventa okay

Is of preventing okay

2. ewe ndiye ndazilibala kwiifoto ezingaphambili ngoku kwathi hayi mani kaloku nale mandiyiveze ngoba kaloku ndingumntu otya ipilisi zokusitopa iTB kusasa um um but aku...zityiwa kusasa sometimes nakusasa ndingabi nanto yakutya mhlawumbi into yokutya ndiyokuyifumana ngokuhlwa...ngokuhlwa nditya le yangokuhlwa so mna sendizidibanisa zonke kangoku ibezezipilisi ziyi4 because ezi ziyi3 zidibane nale yasebusuku nditye kangoku ukuba ndiyifumene into yokutya ndibe ngoko nditya yonke itreatment yam

Yes because I forgot to mention it earlier. Now it comes to my mind that I must even disclosed that I’m someone who in the morning takes the pills to prevent TB... yes but there...they are taken in the morning even then sometimes it happens that I don’t have something to eat and I get it in the evening. Then in the evening I take the one (pill) of the evening, so I add them together with the ones of the morning to make four, because these three add up this one that is taken in the night. Eat then if I have something to eat, and it happens then that I have taken all of my treatment.

1. ngoku eklinikhi ubukhe wamxelela usister uyithatha ebusuku ngoku itreatment yakho?

Then now you have explained to the sister that you take your treatment at night time?

2. andikhange ndimxelele unesi ubayibayi..ukuba ndinamaxesha okuyithatha ebusuku ndinamaxesha okuyithatha ebusuku xana ndingakhange ndibe nanto yokutya eku..ekseni. andikhange ndimxelele unesi ukuba ndenza lo nto ngoku..ngoba ngelinye ixesha andifumenanga into yokutya fori kuba nditye ezipilisi zam zakusasa mna ndizenzele nditye naleeya yam yangokuhlwa mhlawumbi ngokuhlwa sele ndiyifumene kangoku into yokutya ndizidibanise zonke

No I didn’t tell nurse Byebye... if sometimes I take the treatment at night when I didn’t have something to eat early... early in the morning. I didn’t tell the nurse if I’m doing that...because sometimes I go on without finding something to eat so that I can take my morning pills

1. okay enkosi singegqitha

Okay thanks, we can proceed

2. singadlula ke

We are proceeding then

1. foto namba 5

Photo number 5

2. foto namba 5...foto namba 5 naye ngokunjalo ngelixesha ndibasemthandazweni bendikhe ndasemthandazweni ladlula ke nalo ixesha lokutya iipilisi ladlula ixesha lokutya iipilisi ipilisi ndayitya leyithi kuba bendisemthandazweni

Photo number 5... photo number 5. Even it by the way in this time I attend mourning prayer assembles. I was once in the mourning assemble then the time to take pills went past – the time to take pills went past then I ate a pill later because I was in this assemble.

1. foto namba 6, ufuna uthetha ngale

Photo number 6. Do you want to speak about this one?

2. masithethe ngale

Let’s talk about this one

1+2. foto namba 6

1+2. Photo number 6

2. foto namba 6 apha kusekliniki ikliniki le klinikhi iklinikhi ndiyayithanda kakhulu iyandikhuthaza malunga netreatment iyandikhuthaza ukuthi manditye itreatment and u.. isihoyo inkathalo ikhona kuyo...kwiklinikhi endihamba kuyo. Ndiyayithanda kakhulu indinika umdla fori ukuba mandiqhubekeke khona oku ndiqhubekeka oku ndithathe itreatment yam

Here is a clinic, a clinic, this is the clinic. I love the clinic very much. It motivates me about the treatment. It motivates me to take the treatment and a... and the hospitality is there in it... in the clinic I attend to. I love it a lot it rejuvenates me to go on taking, yes go on taking my treatment.

1. O! That’s nice

2. mmm uba nosana lwam bayandikhuthatza kakhulu uba usana luxhomekeke kwalapha kum kufuneka nditye itreatment ukuze usana lwam ndizolibona lukhula ndingabi nakugula ndibe noshiya usana lwam

Mmm! To be with my child, they are encouraging me a lot because the child is depending on me as well. I must take the treatment so that I can witness my child growing up, so that I don’t get sick and leave my child behind

1. okay

2. ewe

Yes

1. siyayigqitha na le

We are going forth

2. siyayidlula na le. Sidluleee. Ingathithi kanti ziphelile...ziphelile

We are going forth and this one... we have went past... let’s just hope they are not finished...they are finished

1. ziphelile kangoku?

Are they finished?

2. ziphelile kengoku

They are finished then.

1. Okay

2. nantsiya yeyendlu...leyaa yindlu ke lena foto namba 7

There it is...is of a house...that one, this is a house. Photo number 7

1. namba 7 umum

Photo number 7 yes

2. yindlu ke le yindlu just nje yindlu diyayibawela ndiyabawela ubasemagunyeni diyayibawela indlu kakhulu indlu eyeyam khona ndizokutya itreatment...itratment yam ndigamane ndiyitshava ndiyazi ukuba itreatment yam ukuba ibikula wodrophini isewodrophini qha indlu ibe yeyam ndodwa kunye nosana namasana am omabini. Yeyona nto ndiyibawelayo ke mna apha ebomini bam ndozotya itreatment yam kakuhle

This is a house this is just a house. I’m liking to have one, I like the idea of owing my own house where I will take my treatment... my treatment without hiding it now and then. I would put it in that wardrobe, it is in that wardrobe, the house where it would just be of me together and my child – together with my children both. That is the thing I would like to have in my life to take my treatment very well.

1. so lento yokuba ungabinayo indawo eyeyakho okanye indlu yakho yenza ntoni apha kwitreatment?

So this issue of not having a place or a house of your own what impact it does on taking treatment?

2. le nto yokubana ndingabi nandowo iyeyam iyandiphazamisa kuba ndihleli nje nditshavana netreatment kanti ukubana bendinendawo eyeyam bendizoyazi ukubana erumini yam endaweni ethile kunepilisi zam ngexesha lam ndisiya erumini yam nditya ipilisi yam

This thing of not having my own place makes me struggle because I’m always hiding the treatment but if had my own place I would know that my pills are inside my room in such a place I was going to go to my room and take my pills.

1. Okay

2. ngoku kangoku xa ndisendaweni engeyoyam kufuneka

Now when I’m not in a place of my own I need to

1. utshintsha tshintshe

Keep on changing places where you hide your medication

2. ndiyazi ukuba ndiyayitshava endaweni...iipilisi zam ndiyazitshava...ndiyazifihla..nditye...iyandiphazamisa kakhulu

To know that I hide it in a place… I hide my pills… I keep them in a safe place…then take them…this gives me some problems

1. Okay

2. Um um

yes

1. masibe

Let’s

2. lena ke yifoto namba

This one, is photo number

1. 8

2. lo ngufoto namba 8 yinto nje endiyizobileyo ngamehlo nagmehlo la ndiwazobile andiyazi ndizokuthi ngawakabani na

This is photo number 8. It is just something I have drawn. These are eyes, these are eyes, I have drawn them. I don’t know I will refer to them as of who.

1. awasemahle

They are so beautiful

2. ngamehlo la xandingatyanga mhlawumbi ndingatyi kusasa ndingafumani nalookofu nallobreakfasti ndithi mhlawumbi ndingafumani naloodinara mhlawumbi ndiyokufumana xana liqala ingathi liyatshonisa ilanga into iyenzekayo kum mna uzobona ngamehlo am ndingafuni nokuthetha amehlo am abomvu

These are eyes. When I didn’t eat maybe in the morning not even had a coffee or a breakfast, even such dinner, maybe get something to eat in the evening. The thing which happens to me is, you will notice with my us, I don’t even want to talk then. They become so red…

1. owu

O!

2. ukuba umntu uyandibuza ndivele ngoku ndifune ukhala

If somebody asks me why, I begin to cry

1. owu

O!

2. ngoku kuba kufuneka ndiexplainile ukuba kwenzeka ntoni na apha kum. Ndingumntu funeka ehleli nje uyashwabadisa uyatya noba utye ntoni kufuneka nditye nditye kancinci ndifumane ngexesha elirythi ngexesha elirythi andityi kakhulu nditye nje ngexesha elirythi nditye

Because now I have to explain what is happening here at me. I’m kind of a person that needs to eat every time. Take bits and bits of food now and then. I don’t eat much. I have to eat get food to eat at the right time, eat at the right time.

1. um um

Yes

2. But ukubana andityanga kusasa andifunanaga ti andifunanga brakfesti mhlawumbi ndofumana xana ingathi liyatshonisa ilanga ndiyabona ndibabone ndiyabonwa nangu boyfriend athi yintoni amehlo ndifune ukhala ndimphoxe yena uzobe engekho mos ebesezijopini ndimphoxe ke yena ke ngoba uyayazi ingxaki um um amehlo am ababomvu kakhulu

But if it happens not to eat in the morning, not to have tea or a breakfast, maybe get it when the sun goes down. I notice and I noticed them, even my boyfriend notices. Then asks what is wrong with your eyes. Then I would want to cry. Slash him with words because he is always away attending his jobs, slash him because he knows my problem. Yes my eyes become so red.

1. um um

yes

2. ndinaloongxaki ki ke

I have such a problem

1. hayi ke

O no!

2. ziphelele kangoku apho iifotos

We have come to a last photo in this way

1. okay hayike siyabulela kakhulu sisi

Okay, o no we thankful a lot sisi

2. Alright

1. siyabulela ngembheko yakho nokuba usinyamezele

We thankuful with your respect to take time on listen from us

2. okay ke mama enkosi

Okay mama thank you

1. e e

yes

2. owu hayi nam ndabulela ngokusebenzisana kunye nani

O yes even me I’m greatful to work with you
