# Supplementary material for: “If I don’t take my treatment, I will die and who will take care of my child?”: An investigation into an inclusive community-led approach to addressing the barriers to HIV treatment adherence by postpartum women living with HIV
Source: PLoS One. 2023 Apr 20;18(4):e0271294. doi: 10.1371/journal.pone.0271294 (PMC10118130; doi:10.1371/journal.pone.0271294)
Supplement: S5 File — (ZIP) [file pone.0271294.s005.zip › 34 18 4th 180531_0036.docx]

1. Good afternoon today I’m with the participant number 3 1 4 8, she got 3 photos and she would like to talk about. Ungaqala ke sisi uqale uthethe ngophoto number 1,

Good afternoon today I’m with the participant number 3 1 4 8, she got 3 photos and she would like to talk about them. You can begin sisi to speak with photo number 1.

1. Ufoto number 1 yifoto apha eneklasi esasingenela kuyo eneklasi, kulefoto into eyenzekayo. Iyandikhumbuza, yintobana ndandikhuthazeka kakhulu zeziklasi ngesizatho sentobana kweziklasi ndaye…phambi kokuba nditye iipilisi kwakuba buhlungu kakhulu ngelo xesha because ngokuya sasisenza eziklasi wawusiye ufumanise intokuba before utye ezipilisi funeke wazi ukubana ipilisi ityiwa njani na

Photo number 1, this is a photo that has a room that we were attending a class in it, what is happening in this photo, it reminds me of how I was motivated by these classes because of this reason in these classes I began to…before taking these pills it was strenuous because in that time before taking these pills you were suppose to know how to take it first

1. Um u

Yes

1. Now kangoku into eyenzekayo kwakubabuhlungu because kwakufuneka, la mntu, umzekelo umntu ogula kakhuulu ohamba ngewheel chair

Now what was happening, it was painful because you had, there was that someone maybe, a very sick person coming with a wheel chair

1. Um u

Yes

1. Umfumanise into bana nemilomo uyinto enje but kufumaniseke kufuneka ezile azothatha le pilisi ngoba kufuneka ayifumane. So eyona nto eyayindihlupha ndandihlutshwa ngababantu. Iminqweno yam eyabangela intobana ndithi intobana iyandikhuthaza yinto bana ndandithi xa ndibona ababantu bagula kakhulu ndiqonde eyi andibaweli de ndifike kule meko yalomntu

Find even his or her mouth is something horrible that you cannot explain but he or she must be there to take this pill because he or she must get it. So the only thing that troubled me was the sight of these people. The hopes that made me to motivated was this I said ‘eyi I don’t want to get sick until I reach the stage of this person’

1. Um u

Yes

1. Ndiyacela into ba ndide ndiyipase iklasi ndizame ngakho konke endinakho ngoba babendenzela iworry because umntu uqeshe imoto ngokuza kwakhe kule klasi kuba enonelele ukuba afumane iipilisi ancame eze, cinga kangoku umntu ongaphilanga kunzima nokuhamba kakhle kakhle so mna into eyayindeza iworry yayinto kubana, ndandingafuni intokubana ndide nditshone deskhe ndigule ndibe kule meko although ndingazange dikhe ndigule nje but ndandingafuni de mandigule

I asked for the speed up of these classes so I can fish quickly with them because these people made feel sorry about them because they hired cars to come to these classes so that they can get these pills, then sacrificed everything and come, can you imagine then someone who is ill struggles even to walk, the thing that worried me much was this I did wanted to gravely ill until be in that situation, although I was never sick but I didn’t wanted to be sick

1. Um u

Yes

1. Yayibabuhlungu kakhulu la nto leyana. Umntu umfumanise uphelile ngamathambo so yeyona nto yandikhuthaza ukubana iipilisi zibalulekile

That scene was very painful. To find out someone is skinny she or he is the bones, so that sight is one which motivated me to understand the pills are very important

1. Okay
2. Kakhulu

Great

1. Um u

Yes

1. Zibalulekile iipilisi kakhulu because lamntu, ndiyacinga into bana mhlawumbi omnyye akasazanga isitatus sakhe wahlala

The pills are very important because that person, I think he was not aware about his or her status and the she sat

1. Um u

Yes

1. Omnye mhlawumbi uthe ezinikwa iipilisi akafuna ukuzisebenzisa, so kemna ndandingafuna ukuba kobobantu

Maybe someone of them when she or he was given the pills but she or he refused to use them, so I did to be counted among those people

1. Um u

Yes

1. So that’s why ndisithi le picture lena indikhumbuza kwezaklasi

So that’s why I’m saying this picture reminds me about those classes

1. Um u

Yes

1. Iyaaa

Yes

1. So kweziklasi bebengekho abanye ababedla ngokusherisha ukubana kutheni bade bafike kwiimeko yokuba babe bagula ngolahlobo because abanye mos njengokuba ndi..ngokuva kwabanye bathi bebengazisebenzisi basebenzise mhlawumbi izinto sesiXhosa neento

So in these classes they were nobody among these ones who were not sharing why they waited until they were in a situation which led them to be sick like that because others…by hearing through others they are saying these one were not using these pills – they were using traditional medicines

1. Lamixube

Those mixtures

1. Eee

Yes

1. Yhaa, baye babe khona abatshoyo omnye athi ndandigazazi ukubana ndiHIV,

Yes, there were those one who confessed by saying I didn’t know that I’m HIV,

1. Ooo
2. Ingqondo yam yayingandixeleli ukubana ndiHIV, ndandiye ndithathe intakatho, ndandiye ndibone ukubana ndithakathiwe

My mind didn’t told me that I’m HIV, I believed that I’m bewitched, I was believing that I’m bewitched

1. Ooo
2. Then kangoku nditye lamixube but ngokuye kangoku mhlawumbi lamixube mhlawumbi umntu iye yamkhulula, ba uthi iye yamkhulula ndibheke ekliniki ndiphelelwe ngamandla kwa xa etestwa kwafunaiswa intokubana ndiHIV

Then drink those mixtures but after maybe after I have taken that mixture maybe someone’s stomach get lose, say that mixture made her or his stomach lose, go to the clinic very weak without the power, when he or she is tested to be found out he or she is HIV

1. Um u

Yes

1. E e

Yes

1. Uqale late kwakuqala oku

Then you begin late in the begin

1. Uqale late

She or he started late

1. Ukuzazi

To know her or his status

1. Iya kuba bengazazi isitatus sakhe

It happens that she or he didn’t know her status

1. Okay yhaaa, photo number 2 ndiyabona ke apha…ubozothini apha

Okay yes, photo number 2 I can see here…what you were going to say

1. Kufoto number 2

In photo number 2

1. Um u

Yes

1. Kufoto number 2, ndifuna ukuthi aphakwipilisi ezi ndiyakhuthaza kakhulu ukuba abantu mabazitye ngoba nam ndakhuthazeka ngesizathu sento bana omnye umntu uye acinge xa eHIV uphelelwe bubomi

In photo number 2, I want to motivate people to take their pills because even me I was motivated to do so and was motivated because of this reason other people think that when you are HIV you don’t have

1. Um u

Yes

1. Kanti akuphelanga bomi apha, unakho uba nefamily uyabeleka unabo nabantwana ngoku uHIV

And here it is not the end of the life, you can have a family, you can have children even if youHIV

1. Um u

Yes

1. If umntwana wakho umkhusele

If you have protected your child

1. Um u

Yes

1. Nawe watya iipilisi, awuphelelwanga bubomi unakho nothenga nemoto

And taken your medication, it is not the end of your life you can even buy a car

1. Um u

Yes

1. Uyaqonda. Akukho nto imisayo kuyo yonke le nto uHIV so kunyanzelekile ukubana yenza le nto uyicingayo ngexesha ungamisi ubomi bakho ngenxa usithi uHIV if uhleli ezipilisi uhlale ezipilisini qha kubekanye ubheke phambili nobomi bakho that’s why ndisithi ndikhethe le foto njena iveza ifemeli because mna

Do you understand, this thing of HIV stops nothing, so it is to endure and do what you think at a very right time, not stopping anything because of saying you are HIV if you stick in the pills stick in the pills once and go forward with your life that is why I said I have chosen this photo which reveals a family because i

1. Ufuna uthetha ngefemeli apha

Do you want to speak about a family here

1. Ndifuna uthetha ngefemeli yam apha

I want to speak about my family here

1. O
2. Ndinabantwana bathathu ndihleli kwezipilisi. Njengokuba ndinabantwana ndihleli ezipilisini ndimi ngxi ezipilisini ndim lo andigungqi andithenini

I have three children whilst I’m in this medication. Whilst I’m having these children I’m in these pills, I’m standing still, here am I, I’m not shaking at all

1. Bonke abantwana ubafumene sele usitya iipilisi

You got these children when you were taking these pills

1. Ndibafumene sendisitya iipilisi

I got them while I was still taking these pills

1. Nalo wokuqala

Even the first one

1. Ndizazi kulo wokuqala ukubana ndiHIV

I knew that I’m HIV positive through the first one

1. O
2. So that’s why ndisithi, phambili ngepilisi phambili

So that’s why I’m saying, forward with pills forward

1. Mmm, so ikunika umdla wena

Mmm, so this encourages you

1. Indinika umdla kakhulu kangokuba andiqondi ndoze ndiphulukane nayo

It encourages me greatly and I don’t even think I will ever depart with it

1. Um u okay

Yes okay

1. Kufoto number 3

In photo number 3

1. Okay masiye ke kufoto number 3

Okay let’s go to photo number 3

1. Unesi lo

Is not the nurse this one

1. Oh hahahaa hayi bendingamboni

Oh hahahaaa no I was not seeing her clear

1. Ngunesi lo

This is a nurse

1. Hayi ndiyambona

Yes I can see her

1. Nantsi into yakhona. Kulo foto number 3

Here is the thing…in this photo 3

1. Um u

Yes

1. Mci! Eyi! Ngexesha ndandiHIV kule foto number 3

Eish! By this time in this photo number 3 I was HIV

1. Um u

Yes

1. Mosi njengokuba ndingumntu, umntu umzekelo athi ndiyagula okanye mhlawumbi sithi sisekliniki mhlawumbi mna ikhona into endiyeleyo ufumanise intobana pha kwikliniki endandihaamba kuyo ndigahambeli ukubana ndingekazazi fumanise ukuba kwakukho igumbi elali lodwa apha ekliniki ela gumbi

Mosi as am I a human being, someone maybe say for an example maybe when we are inside the clinic maybe I’m there something that needs me, you will find out in the clinic I used to attend, not attend because… I was not aware then about my status, find out that there, there was this room that was standing separately from others in that clinic

1. Um u

Yes

1. Should ba ungene kulo uyaziwa ukubana uyele ntoni na

Should you enter to that room, everybody already knows what takes you that room

1. Um u, yayilingumbi lantoni lokutesta na?

Yes, what was that room for, to test?

1. Yayiligumbi lokutesta

It was a room to test

1. Oh
2. Nolokuthatha iipilisi

And to take the pills

1. O
2. So ndaye ndathi ndoku…ndifumanisa ukubana kukho ela gumbi ndonqena ndakhe ndafuna ukrukruza uthatha iipilisi although

So when I was…when I found out what that room was standing for, I got scared and I started to drag my leg to go for my HIV

1. Wawusele uzithatha wena ?

You were already taking them?

1. Ndandingakazithathi, ngexesha ndadizongena ezipilisi ndandingayazi intobana kwezinye iindawo kwenziwa njani ni na okanye kule ndawo ndiqale kuyo uthatha iipilisi kwenziwa njani ni na

I was not taking them, when I was going to take the pills I was not aware about things are being done in other places or in this place

1. Um

Yes

1. So ndandinakho ukufukufukuza ndiqonde eyi ngexesha ndizoqala ukuthatha iipilisi ukubana ndithathe itransfer from kwesosibhedlela ndanditeste kuso ndangenela kuso iiklasi ndandiqonda eyi ndiyoyika ukuthatha iipilisi because izinto ezithethwa ngabantu phandle apha kukhona kuthiwe iinesi ziyaphuma nezitatus zabantu uyabona that’s why ndithathe le picture yabona because nazo iinesi bezichaphazeleka

So I had some doubts before the arrival time to take my treatment, thinking of taking a transfer from this hospital I was diagnosed in it which I even attended classes in it. I was scared to go take the pills from it because the things people were talking about outside, saying that the nurses are going to reveal the status of those who tested, you see that’s why I have taken this picture you see even the nurses were involved. I was scared to go take the pills from there because of what people were saying about the nurses

1. Um u ooo

Yes ooo

1. Apha ekuthatheni iipilisi a endaphe umntu athi ebefuna ukuyozithatha iipilisi angoyothatha iipilisi ngoba kuthiwa iinesi ziphuma nezitatus zabantu

When someone is taking the pills, some people ends up not taking them even if he or she wanted to because it is said that nurses go out and reveal the statuses of those who that were tested

1. So ngokunokwakho apha uzama uveza intokuba unesi ngokwakhe angenza umntu adileye uzokuthatha iipilisi

So here you are trying to reveal that even a nurse can make someone to delay to take his or her pills

1. Exactly
2. Okanye angayithathi itreatment

Or not even taking his or her treatment

1. Iyhaa

yes

1. Kwindlela le baqhuba ngayo

The way they are doing things

1. Baqhuba ngayo

The way they are doing things

1. Ezikliniki

In clinics

1. Ewe because abanye abantu yisecret yabo le na njengokuba sekhe ndatsho kwenye ifoto ukubana. Into yokuthatha iipilisi yisecrete yam

Yes because other people, this is their secret, I even said by other day in another photo that, the thing of taking the pills is my secret

1. Um u

Yes

1. Yaziwa ndim nefemeli yam if ndikuxelele so andilindelanga xa umntu

The people who know it, is my family and me, if I have told you I’m not waiting for someone

1. Xa uye ekliniki

When you going to the clinic

1. Njengokuba endinika iipilisi pha ekliniki azithithi dlivi ngaphandle kwabantu

While she or he is giving me the pills just put on the table in front of the people

1. O
2. yabo so that’s why ndiveze intobana nenesi noqala ithi xa imnika

do you see that’s why I’m revealing even a nurse in the first place

1. iba yichallenge

it becomes a challenge

1. yes
2. o
3. that is why ndiyifotile intobana mayaziwe ukubana nyani nam njengokuba ndithetha nje ndandi

that is why I have photographed that people must know this that even as I’m putting it that

1. wa deleya okokuqala

you delayed

1. zange ndideleyeye more than into yokuba yakhe yafika lo nto leyo engqondweni kum ndaba buthandabuza intobana ndiyithathe ipilisi ngesizatho sokubana kwakuthiwe nenesi kangokubana ngelinye ixesha ndandinento yokuba eyi ndibawela utshekisha ngubani inesi ekhoyo okanye uthi ngexeshe sele ulapha ekliniki uqonde ulaqaza kwa abantu aba

no I did not delayed more than that this came to my mind, having some doubts because of the reason that people said about the nurses, then I was doing this, tried to check which nurse is there first or when I’m already inside the clinic look around

1. um u

yes

1. ukuba I wonder akukho mntu undaziyo na

to wonder if there’s no ones who knows me among the people who are there

1. um

yes

1. eyi ndingena ke, elagumbi I wonder akukhonto laziwa ngalo na ukubana xa ndingene kwelagumbi ungena kuba uHIV sendisitsho ubana uyabona xa uHIV sesona sigulo esi mna ndathi ndozijonga ndazibona ubana ingathi ndonile phambi kobuso buka yeHova

then I enter, still speculating if this room is not special of a known thing, that you go in that room because of you are HIV, I’m saying this because when you are HIV, this is the only sickness that made me to think when I looked at myself as if I have sinned before the face of Jehova (God)

1. um u

yes

1. xa ndandifumanise ukubana ndiHIV yangathi ndenze eyona nto iwrongo although ngelixesha ndifumana isifo ndingazazi ukubana ndifumana isifo na ngoku okanye ndingayazi nokbana kwenzeka ntoni na yabo

when I understood that I’m HIV it was like I have done something which is wrong although I didn’t know that I was getting the disease when I was getting it or not even aware of what was happening you see

1. um u

Yes

1. that’s why ngoku uye ufumanise ukubana omnye uye angazithathi iipilisi uye azizonde. Omnye athi babekhona abanesibindi bathi asisosifo sazinja aphinde omnye athi eyi I wonder umyeni wam I wonder iboyfriend yam xa indiva ndinjena I wonder usister wam xa endiva ndinjena uzophinda andithande I wonder apha ekhaya andizocatshukelwa…andizocatshukelwa na apha ekhaya ndithi noba umzekelo ndivase izitya okanye icephe ndizocalucalulwa ngamacephe ngoba uqalakwaso esisifo abantu babenezitya zabo

that is why you will find out some hating himself or herself abandoning his or her treatment because of that. And you will find those one who courageous by saying this is not a dog’s disease and againg hear someone saying eish I wonder what my husband is going to say when he hears that I’m like this or I wonder if my sister will she love me again when she hears that I’ like this, then again says I wonder here at home they are not going to hate me… are not going to hate me, for an example when I’m doing the dishes, washing a spoon I have used, aren’t they going to say they don’t want to use the same spoon, because when this disease started people had to have to use their own plates?

1. um u, so ibinabo ubunzima, ubucinga kaninzi before uqale

yes, so we are still having some obstacles, things that makes you thing hard before you start

1. ewe

yes

1. um u

yes

1. ubucinga

think around

1. bekungelula uvele uqale wena

it was not easy for you to start

1. bekungekholula although

it was not easy although

1. uyokuthatha

to go and fetch

1. intobana bendibawela ukuba impilo yam ndibesafe

that I wanted my health to be safe

1. um

yes

1. ewe ubusafe bona bendibufuna kangokubana intobana…ndaye ndayicinga kancinci lo nto leyo intonbana eyi ndinesisifo ekugqibeleni nezipilisi bayazincoma intobana abantu bayancedakala kuzo, so yintoni kangoku ezondenza mna ndidileye uzibuza xa unesisifo uziphendula uzibuze uziphendule because sesona sifo besize kakubi besisistrongo kuqala

yes to be safe that I wante even that… I thought for a while and I came to realized that at the end I’m sick and I even heard from other people that these pills they do help, people were helped by these pills, so why I must delay, you see when you are having this disease you converse with yourself a lot, asking yourself questions and answering yourself as well because this is a disease that came with a full force at the begining

1. um u

yes

1. simnandi ngoku usicontrola ngokwakho

it is enjoyable now because you just control by yourself

1. ewe, ngubani kengoku unolwazi

yes, now who is knowledgeable

1. uphela unolwazi ngoku

then you end up having this knowledge

1. um u

yes

1. uphela unolwazi ngoku because ekugqibeleni ezizinto ubuzifundisiwe okanye unakho intobana uxelele umntu intobana hayi mani nam nditya iipilisi itya iipilisi

you end up having this knowledge because you were taught these things or you advise someone that no don’t do this do that, I’m taking this medication, take yours too

1. abantu bayashera ngoku

people are sharing

1. abantu bayashera yabo

people are sharing you see

1. iiformation

informations

1. abanye bayakwazi intobana ngelixesha kufuneka betyile iipilis abanye bayakwazi intobana ngelixesha kufuneka betye iipilisi umntu azithathe umzekelo nam ndinaye omnye okay qha igama lomntu alibizwa ukhona omnye ebendimcenga gqithi

others they do this, when the time to drink pills, take them, I had a person that I know, but I’m not mentioning names, for an example

1. um u

yes

1. fumanise uzithatha azifake phantsi komqamelo kodwa ndingasichazanga isistatus sakho

find out that she or he takes them and put them under the pillow but not mentioning my status

1. o
2. ndimcenga qha khawukhe unyamezele apha kwezipili

I was encouraging her to stay on these pills

1. um u

yes

1. kodwa uThixo wala ngoba wayengasamkelanga

but God denied her or him a chance to leave because she or he didn’t accept the situation

1. um u

yes

1. yhaa

yes

1. um u, hayi ke ndiyabulela, sabulela kakhulu yiveki yakho yokugqibela ke le

yes, I’m thankful , we are very thankful this is your last week

1. e e

yes

1. kodwa ke njengokuba sasithembisile asizokwahlukana somana sisiza sofumana neinformation njengokubana sasichazile ukubana, oluphando lelokufumana iiformation kuni ikakhulu

but like we have promised you before that we will be in touch we will always come to get the information as we have said so that, this research needs us to find information in people like you

1. e e

yes

1. siyashera apha nathi sizokwazi kangoku nokubaza izinto ezingasicacelanga sifunda kwalapha kuni

we are sharing here so that we can improve in things that are not important to us learning from you

1. iyhaaa

yes

1. e so yonke into ibalulekile ezizinto uzithethileyo

all these things you have said are very important

1. ewe

yes

1. zibaluleke kakhulu because zizakusetyenziswa nakuba kucediswe amanesi la uthetha ngawo iikliniks namhlawumbi indleala makuqhutywe ngayo nabantu batreatwe ngayo ewe zininzi izinto ezinjalo enkosi kakhulu ke sisi

they are very important because they are going to be used to help nurses the ones you are mentioning, the clinics, even the way things must be conducted even how people must be treated, there’s a lot of things which are like that, thank you sisi

1. alright
2. ndabulela ngesitopu sakho

I’m thankful about your patience

1. okay
