# Supplementary material for: “If I don’t take my treatment, I will die and who will take care of my child?”: An investigation into an inclusive community-led approach to addressing the barriers to HIV treatment adherence by postpartum women living with HIV
Source: PLoS One. 2023 Apr 20;18(4):e0271294. doi: 10.1371/journal.pone.0271294 (PMC10118130; doi:10.1371/journal.pone.0271294)
Supplement: S6 File — (ZIP) [file pone.0271294.s006.zip › 42-18 1st.docx]

18.03.05

V1: And you make sure it’s working.

V2: JA!

V2: YES

V1: And you can put it anywhere it doesn’t matter.

V2: OK

V3: So besilapha mos nge 26

V3: So we were here mos on 26

V2: ewe

V2: yes

V3: kaFeb so icamera numba yako ngu 4

V3: of February so your camera number is 4

V2: 42/18

V3: 42/18 ja! then sizakuthetha ngezifoto zilapha kulenantsika kulecamera yako so singayivula okwangoku icamera

V3: 42/18 yes! Then we are going to talk about the pictures that are in your camera so we can open it now

V2 : akho problem

V2: there’s no problem

V1: Was the camera ok you can use it, u have no problem with it?

V2: no problem its ok.

V3: Sizakufuna ukubona mhlawumbi ifoto yokuqala.

V3: We are going to need to see the first photo.

V2: Ifoto yokuqala nhe? Yeyokugqibela le!

V2: First photo? This is the last one!

V1: So u need to say photo number1.

V3 Sifuna ukubona ifoto yo

V3: We need to see photo number

V2: Ifoto yokuqala it’s a blue card.

V2: First photo it’s a blue card

V3: Yokuqala it’s a 1

V3: First one it’s a 1

V2: This one

V3: The first one

V1: That’s right, you’ve said photo 1

V3: It’s a photo1 nhe!

V1: You must say the date the number of the camera and the number of the photo, so now you are going to do photo1

V3: Ok

V2: Foto 1 iblue card mos pha xa uphaya eclinik kukho ifolder ezi2 phaya

V2: Photo 1 is a blue card mos there at the clinic they use tow folders

V3: Phi ?

V3: Where?

V2: Eklinik

V2: At the clinic

V3: Kwihealth desk okanye

V3: At the health desk or

V2: No! zipha kubo nhe but mandithi zikwi

V2: No! there with them but I should say

V3: Pha kulamagumbi oosister?

V3: At the sister’s rooms?

V2: No! Kulamagumbi emacardi onke

V2: No! At the reception area

V3: Ok

V2: But zinecartbaord zawo nhe le blue card le wena mntu uHIV+ usebenzisa leblue card

V2: But they are in courtboards the blue card is for HIV+ patience

V3: Oh ok

V1: Mmh

V2: Kukho iyellow card bendifuna ukuyifotaa kakuhle apha naleblue card, kukho iyellow card which is azifani ezifolder zethu

V2: There is ayellow card I wanted to take a picture of it with this blue card, the yellow card is for just sick patience they are not the same as ours.

V3: Le iyellow yeyomntu ongekho hiv+

V3: The yellow one is for HIV- patience?

V2: Yes! Le iblue yeyomntu oHIV+ but that time mna ndandifound out mna uba im a hiv+ ndandingayazi le blue card uba yeyantoni nhe

V2: Yes! The blue one is for HIV+ but when I found out I was HIV+ I didn’t what the blue card was for

V3: Lefoto sithetha ngayo apha yeyokuqala sinayo apa uyithathe eclinik nhe?

V3: The photo we are talking about is the first one that you took at the clinic?

V2: No! kuba ndineblue card mna apha ifile nhe e blue

V2: No! It’s just that I have a blue file here

V3: Ikhona apha endlini?

V3: You have it here at home?

V2: Ewe ndinayo mna ayiyo eyaphaya, but ndafumaniseka ukuba indiriminder leblue card ukungazifeely comfortable xa upha eclinic because xa ubonwa pha eclinic uphethu leblue card uyaziwa uba.

V2: Yes I have myown not the clinic’s, but I found out that it’s a reminder that makes me not feel free when im at the clinic with this blue card. When you are seen at the clinic with this blue card you are known that you.

V3: Ok, ustigmatised!

V3: Ok! U are stigmatised

V2: Aba bazaziyo bona ukuba nam ndisebenzisa lento umzekelo asazani phaya eclinic uya ngedate yako uyokutata itreatment yako.

V2: Those that know that they use the same e.g we don’t know each other at the clinic, you go ther on your date and take your treatment.

V3: Because khange udiscloser mntwini akakwazi lamntu

V3: Because you didn’t disclose to anyone so that person doesn’t know your status

V2: But oyena mntu uyaziyo lablue card ngulo naye uyaziyo uyayisebenzi naye lablue card so mna uba ndibona wena

V2: But the only one that will know is the someone that is also using the same blue card so if I see you

V3: Okanye umntu oclose omaziyo osebenzisa leblue card

V3: Or if you know someone close to you that is using the blue card

V2: Okanye mna ndibona wena uphethe leblue card ndiyazikhuzela Oh uLuleka uhiv+ ndibona leblue card

V2: Or maybe I see you holding the blue card and I would be surprised that oh Luleka is HIV+ because I see the blue card

V3: Ayicoverishwanga ngeyellow card?

V3: Is it not covered by the yellow card?

V2: Ayicoverishwanga yiblue card ilelihlobo, ilelihlobo licard eliblue inento zakho ngaphakathi ibhaliwe aph’ igama lakho ngaphakathi

V2: it’s not covered it’s a blue card like this, this blue card has you information inside and it’s written your name inside

V3: aha

V2: Ufumanise ukuba kengoku xa uzokuthatha itreatment yako wena apa eclinic ufumanise bakhona abantu azile wothuke ukuba ubani because lamacard xa ungena aphe clinic kuthathwa ifolder yako ibekwe ecaleni xa uphumayo kuphinde kungene omnye umntu which is wena uzaziyo its easy ukumbona

V2: When you come to the clinic to take your treatment you find other people at the clinic as well and you see they are using the same card as you know because cards are put on the bed and you enter the room and see someone else’s card as you saw them leaving the room. So its easy to know

V3: Its easy ukumbona

V3: It’s easy to see the one that is HIV+

V2: Which is kueasy ukumbona umntu oHIV+ aphelalini because eza blue card zibekwa apa phezu kwebhedi eclinic

V2: Which is easy to spot HIV+ patience her in the village because these blue cards are put on the bed at the clinic

V3: And uyayazi ukuba bekuphuma bani apha

V3: And you know who has just left the sister’s room

V2: So iye indiriminder lento ukuba andikho safe because I know leblue card yeyomntu otheni

V2: So this reminds that im not safe because I know what the blue card id for

V1: And everybody else knows

V2: Ja zange indifeelisher kakuhle that time I had ukuba ndiyi excepter because kaloku ekugqibeleni iyenzeka so zange ndiyishare namntu because ndonqena ukuba ngathi ndiza nezam izinto but then ngoku

V2: Yes I didn’t feel good bit had to except it because its happening so I never shared it with anyone because I didn’t want to be like im coming with my own things

V3: Oh ok so zange uespresser lento kosister nangona ikhona indawo ekukhubekisayo

V3: Oh ok so you never expressed it to the sisters even though it wasn’t seating well with you

V2: but apha uyabona apha as from last year

V2 but here do you see here as from last year

V3: yifoto yesibini le oyitate nini ke yona?

V3 this is the second photo, when did you take it?

V2: same day ngokuya ndanditata le,

V2: same day when I took that one

V3: same dayngale26?

V3: same day 26^th^?

V1 photo2

V3: foto2 oyithate 26 okucamera number 42/18

V3 photo2 that I took on the 26 in camera number 42/18

V2: uyabona ke ngoku as from last yr andiyazi beva ntoni okanye kwathinina but ngoku ifolder zethu ziyafana

V2: you see know as from last yr I don’t what happened or who said what but now our folders are the same

V3: aha

V2: zinje that is why kengoku

V2: they are like this that is why now

V3: as from last yr?

V2: yes

V3: kwibeginning?

V3: at the beginning?

V2: I think it’s the beginning nhe apa pakathi so akekho umntu oyaziyo andiyazi beva ntoni okanye kwathini

V2: I think it’s the beginning or here in the middle so no one knows what happened or anything

V3: but zange uthethe kusister ukuba ufeelisher njani?

V3: but you never told the sister how you feel?

V2: but zonke zinjena ziyafana

V2: but now they are like this, they are the same

(ukukhala umntana,V2 ina bhala ina Ino)

( baby crying, v2 take and write take Ino )

V2: uyabona ziyafana kengoku which is andisenaproblem because umntu uzakucinga ndizise lonto ngiyazigulela kuba ke andisenayo laproblem because ziyafana but iproblem before

V2: you see these are the same which is no problem because now noone will know why I have come to the clinic. They will think im sick just like anyone else. So I don’t have a problem anymore.

V3: le ifoto ikuvisa kamnandi

V3: This picture makes you happy

V2: indivisa kakuhle kuba kaloku umntu akazoyazi ndizokuthina kanti kuqala xa endibona ndiphethe leya iblue uyayazi umntu ndizokuthinina yabona which is yinto endingazange ndayiteta mntwini nam actually andingomntu ufane ashare

V2: it makes me happy because none will know what am I doing at the clinic, at first when they saw me with that blue card they would know why I was at the clinic even though I never told anyone actually im not a person that shares

V3: ok

V2 iproblem yokuba ndingashari kungenxa yokuba uyalibona mos iphepha iphepha uba ubhalile elaphepha liyakwazi ukuwa liphaphatheke licholwe ngomntu afunde ubani ubhale into ethile nento ethile althou kungeko gama lakho but uzakuyiva lento seyithetha ukuba ndichole iphepha elithi elithi but ngencoko ukuba ey andingomntu ufane ashare

V2: My problem with not sharing is that you see a paper mos a paper a paper if you write on it that paper can fly away and someone can pick it up and read what you wrote and you here them talking about picking a paper that has this written on it even though it doesn’t say who wrote it.

V3: ok

V2: uyabona apha apa kuba ndingengomntu ufane ashare I asked someone as nje ingathi ndiyadlala

V2: You see here, here because I’m not a sharing person I asked someone randomly if I can take their picture

V3: ngufoto3 lo

V3 this is photo3

V1: this is photo 3

V2: ja foto3

V2: yes photo3

V3: kulocamera 42/18

V3 in camera 42/18

V2: bendisenza ingathi ndiyadlala apa

V2: I was acting as if im playing here

V3: uyithathe kwisame day?

V3: did you take it on the sameday?

V2: no following day

V3: ucele ukuthatha lomntu

V3: did you ask to take this person?

V2: ewe ndimcele ukuthatha lomntu ndamxelela ukuba andifuni kumveza but I will do example ngawe imbangi yokuba ndifote lomntu iproblem yam as from last of last yr I think Oct Nov uqula kwam ukudibana notatomtana mna ndandicinga ukuba ngumntu oright zange ndiyazi ukuba uzakundiphatela athi akugqiba andishiye nomntana so ndafota lomntu uyindonda kuba mna ndizixelel ukuba asobe ndimthembe umntu oyindoda because ndandimthembile utatolomntana and zange achaze nyani kum.

V2: yes I asked him if I could take his picture but im not going to show his face but I will do example of a man structure. The reason I took this picture is as from last of last year I think Oct, Nov when I started dating the father of mychild. I thought he was a Mr right I didn’t know that he was going to infect me and leave me with a child, so so I took this picture of a man because I have told myself that I will never trust a man ever again because I had realy trusted the father of mychild and he was not truthful to me

V3: nge status sake?

V3: about his status?

V2: ngestatus sakhe

V2: yes about his status

V3 wena ufeeelisha ukuba wayezazi?

V3: you feel like he knew his status?

V2: ewe wayezazikuba because ndadibana naye ndiright afta ndizifumanise ukuba ndikhulelwe lomntana ndikhuleliswe ngowake mos xa upregnant uye utestwe pha eclinic ndafumanisa ngeloxesha and bendingekho nje before bendingekho naye so kuba yena engekho ndatata ipicture nje yomntu oyindoda endizakuthi ndizikhumbuze ngayo. Azange ndifeelisher kamnandi like umntu oyindoda zange ndimthembe emveni koko. Because thina singabantu ababhinqileyo uye umthande emthembe kanti lomntu ayingowothandwa ayingowothenjwa akushiye unengxaki ngasemva ube wena ucinga ukuba uzakuyithini lento nabani, uyabo

V2: yes he knew because when I met him I was right, its after I had fallen pregnant with his child that I found out. When you pregnant mos at the clinic you are tested that’s when I found out I was infected and I was not infected before I met him. Because he is not around I took a picture of a roundom man as a reminder. I didn’t feel good like I will never trust a man after this. Because we as woman we love and trust man and sometimes that man is not worthy of your trust nor love, he will leave you with problems that you are unable to solve

V3: ok

V2: uyabona le ndithe xa ndigubhulula

V2: do you see this I was going through

V3: ngufoto4 esimfumana kwicamera42/18 ngeyiphi idate kanene? Ngosuku lwesingaphi?

V3 its photo4 that we got from camera42/18 which date? On which day?

V1: its ok you can put today’s date

V2: uyabona le ndithe xa ndigubhulula apa endlini ndabona lepaketi nhe lepakathi iye yandireminder ukuqala kwam ukutya itreatment ukuqala kwam uva ukuba ndiHIV+ndafumana lepaketi andiyazi ba yintoni igama layo but ndaye ke ndayitshintsha ekuhambeni kwexesha for iprocess yomntana ndanikwa ezaARV but that time ndindifumana latoti inye letoti i1. But ngoku ndifumana l

V2: you see this one I was going through some things here in the house and I came across it. It reminded me of when I started taking treatment for the first time, when I heard for the first time that I was HIV+ I got this packet I don’t know what its called. I have changed it since for the process of a baby I got ARV’s, but then I was getting 1tin this tin here. Know im getting

V3 ufumana letoti1 nalepaketi?

V3: you are getting this 1tin and a packet?

V2: ndandifumana ngolohlobo kuba kwakusithwa zona ezi zinyusa ICD4 count yomntu

V2: I was getting like that because they said these where bursting the CD4 count

V3 ok

V2: but ngoku ndifuman le uyabonale le yibox le?

V2: but nw im getting this one you see this box?

V1 so this is photograph 6 I think

V3 6 yes

V2 ndifumana ngoke le

V2: im getting this one

V3 uyitate kwisameday or kusuku olulandelayo?

V: did you take it on the sameday or the following day?

V2 hey andibali mna xa ndicinge lento uba mandifote kuthi qatha into ndivela ndifote yabo, apa yitreatment ye2months le like umzekelo mandithi ndiya ngoJan xa ndifika phaya ndifika ngoJan ndifumane itreatment ye2months andibinalo elaxesha lokuthi ndiyahlala phantsi nosister, mhlawumbi kubekho into endifuna ukuyibuza okanye ukuyithatha andibinalo ixesha because ndiyafika phaya ndinikwa laform sendisaziwa mos uba, andithi mos ndifikelwa yisms efowunini yam mhlawumbi ithi nge 6 kufuneka uye eclinic uyokuthatha iparcel yakho so sendisaziwa ukuba ndizolanda labudgeg so andibe ndihlala pantsi mhlawubi kubekho into endiyisharayo ndifika sele iready ndifika ndisigner laform ndihambe ibarget sele isotiwe.

V2: hey I don’t count I just take pictures when ever I think of taking the picture or if I think of something. Here it’s a 2months treatment like if I go to the clinic in Jan

V3 sele isotiwe

V3 its been sorted out?

V2 sele isotiwe ewe

V2 sorted out yes

V3 ikwenza ifeeler njani lonto?

V3 how does that make you feel?

V2 sometimes ayindivisi kamnandi because xa ungumntu unazo izinto ufuna ukuziqonda apa ezinursini ube eloxesha lingafumaneki ungenalo

V2 sometimes it doesn’t make me feel good because sometimes you want to know something or ask something from the nurse’s and you don’t get that chance.

V3 aha

V2 umzekelo ngoku bendiye ngoFeb andizokuya kulenyanaga

V2 for example the last time I went was Feb I wont be going next month

V3 apha kuMarch

V3 here in March

V2:ndizakuya ngoApril yabo ixesha loba ndidibane nabantu abaziproffessionals andinalo. Ndinala date yam yesurey kuba ndifumana la2months. Andibinalo elaxesha lokuthi ndiya eclinic xa kukho into ukhubone because kaloku ekugqibeleni akukwazi ukuya eclinic ungenanto unayo. So ichance yakho wena kukuthi xa usiya eclinic uyokutata latreatment yako ubeyilachance yakho ongathi ushare ngayo because ixesha elininzi ungulo ungayiyo eclinic.

V2 I will only go know in April you see that’s the only time that I can see a professional. I have that date since I get 2months treatment. I don’t have time to go to the clinic when there’s something because in the end you cant just go to the clinic if you are fine. So the only chance you get is when go to get your treatment only then you can share anything with the nurse.

V3 should ba usiva mhlawumbi ayikuqhubi kakuhle itreatment ngendlela oyithatha ngayo ngelinye ixesha nangona uyithatha itreatment uba nayo lonto noba yintloko awukhe uziphe ithuba lokuya kosister uyokuqonda ukuba xa kuthe suba kutheni

V3 should you fill something wrong with yourself even though you take your treatment regularly, do you go to see the nurse even if its not your date just to know what is wrong?

V2 no andikhe ndiye because kaloku ukhubone ndiye ndixakwe ukuba ndizakufika ibe ingeyodate yam ndithini ndibe ndingengomntu ufane aye eclinic, ndiyakwazi ukuyigqiba i3months ndingakhange ndibe nanto ibuhlungu endithi ndiyisa eclinic

V2: no I don’t because I get worried about what am I going to say especially because it not my date, I don’t get sick often sometimes three months passes without me getting sick

V3 ok but ayiyichaphazeli indlela oyithatha ngayo itreatment apha endlini

V3: ok but does it not affect the way you take your treatment here at home?

V2: haha

V2: no

V2 apha

V2: here

V1: photo 7

V2: ngu foto7 lefoto ngumntanam lo imbangi yokuba ndimfote yintoyokuba ndiye ndizibuze umbuzo ukuba xa ndinongayityi le treatment umntanam uzakushiyeka nabani uzokuhoywa ngubani yabo mna phofu ndiye ndithi noba andifuni mos like libekhona elaxesha lokuba udikwe zipilisi but ndizibuze umbuzo wokuba xa ndizakudikwa zipilisi umntanam yena xa ndizoyitya kakuhle itreatment umntanam ndizomshiya nabani. ( khandiphe nana, enkosi) umntanam ndizakumshiya nabani? Le ifoto, ikhona enye apa

V2: this is photo7 this is mychild the reason I took a picture of her is that I sometimes ask myself that if I don’t take this treatment who will look after mychild who will take care of her. So even if I feel like not taking my pills im forced to take them for mychild’s sake. (give it here nana, thanks) there is another photo here

V1: photo 8

V2: foto 8 lo ngumzali wam lo ungumntu ogula ngesifuba swekile ihigh blood,arthritis and also ulser, imbangiyokuba ndimshute last ngo dec 27 umamam wayengekho esisbhedlele egula phaya endlini kuye kubuye ingqondo zentoba umamam uyazigulela naye unento zakhe so na xa ndingenozimisela apha etreatmentini yam umamam egula, andishiye umamam nam ndibe ndingekho umntanam uzakushiyeka esithini so yilnto ibangela ukuba ndizimisele kwitreatment yam because ndinalento umamam uyagula kwelakheside unezinto zakhe also nam xa ndingenozinakekeli mna ngokunokokwam ngubani uzakunakekela umntana wam ngubani uzakunakekela mna.

V2: photo8 this is myparent she is someone who is suffering from Diabitis, high blood, arthritis and ulser, the reason I took this on the 27^th^ Dec my mother was sick and had to be admitted at the hospital. Sometimes it makes me think that if she leaves me there wont be anyone to take care of mychild or me.

V3: lonto ikwenza uzive njani?

V3: how does that make you feel?

V2: lonto indenza ndizive kabuhlungu, imbangi yokuba ndithi ndizifeeler kabuhlungu kungenxa yokuba letreatment andizoyitya mna ndizabe ndingekho umzekelo ndiswelekile umntanam uzakuphateka njani xa mna ndingekho? Akazothukwa umntanam kuthwe umamakho wabhubha yinto ethile uyabona into ezinjalo zezonto ke zindibangelaba

V2: it makes me feel sad the reason I say it makes me feel sad is that if I don’t take my treatment I will die and who will take care of mychild. She will be called names and be told “your mother was killed by the virus” things like that

V3 zikukhuthaze?

V3: so that make you want to take them regurlary?

V2: ndibe ngathi ndiyaqiniseka

V2: regurlary

V3: ikukhathaze

V3: it troubled you

V2 apha ngumntu wefamily lo

V2: here it’s a family member

V1: ok

V2: sometimes thina kwezizstage zethu uyakwazi ukushare nomntu wefamily ucinge ukuba lomntu wefamily uyakuvela

V2: sometimes we at mystage you can share with a family member hoping for a choulder to cry on

V3: nguphoto 9 lona nhe?

V3: this is photo9 here?

V2: ayingo10?

V2: is it not 10?

V3: hayi ibisandokuthi8

V3: no its just said8

V2: mos uyakwazi ukushare nomntu wefamily ucinge uyakuvela uva kabuhlungu but at the end lanto bendiyishare nawe mna siyithetha sobabini

V2: mos u can share with a family member thinking they feel for you but at the end what you shared with them

V3: kuba undithembile

V3: because you trust them

V2: ndiyive ithetwa ngomnye pha okanye bekusesimokolweni ubani wathi lonto indibangela ukuba some of abantu basefamilini ndingabathembi nhe lento ndiyitetayo its because of yandehlala mna ndafowunelwa mna ngumntu ba fondini zime ngawe ubani uthi uthi lomna bendimthembile ndimchazela ngalengxaki yam ndiqonda yena uzakukwazi ukundi encourager andichazele ukuba mandithina. Yangulo ke ngoku uyithethayo intlungu yam wahlekisa ngentlungu yam ndibe mna ndingekho kulonto

V2: then you here other people taking about what you have discussed in privacy wiith the family member be it in a sheeben I don’t trust some family members. I have expierence with what im saying I told a family something about myself later I got a call saying news are about you here so and so is taking about you. When you taked with tht person you needed encouragement and guidance but instead she decided to tell everyone you problems.

V3: lonto yakwenza waziva kabuhlungu?

V3: how did that make you feel?

V2: yandenza ndaziva kabuhlungu kuba kaloku nabanina ngoku ozakuhamba apha uzakundijongela lonto ndibe mna bendiyixelele umntu omnye oyifamily lomntu wefamily ngulo uhambe ethetha ngam

V2: it made me feel sad because people are going to look at me in a strange way from now on

V2: ikwenza uzive njani ngoku malunga nokuthatha ipilisi zakho, ayikwenzi ukuba mawungafuni kutya

V2: how does it make you feel about taking your pills?

V2: no

V3: ufune ukuzufihla xa kufike abantu abathile libe libethile ixesha

V3: do you hide them when there are people in your house when its time to take them

V2: ja like umzekelo yabona apa bendiyiphethe apha

V2: yes like here for example here I had it with me

V1: this is photo 11

V1: this is photo11

(Enkosi hambo nika uNako, hambo nika uLerato)

V2: apha bekusebusuku besiceliwe ngumntu ukuba masiyokumtsala nemoto ngapa but khange ndikwazi ukuyitya ne because ndiye ndacinga ukuba bazakuthi yintoni le ndityayo and kube kusebusuku kubusy ndanoloyiko lentoba inoba bazakundibona mhlawumbi

V2: here it was at night we were asked by someone to help with their car, I couldn’t take it because I thought what will other people say what am I taking

V3: uyiphethe apha kuwe itreatment?

V3: you had your treatment with you?

V2: ndiyiphethe apha kum but ndanoloyiko lokuba inoba bazakuthini mhlawumbi kuba ndingumntu oqhele ukuhlala yedwa apha endlini andisokoli ngexesha lam ndiyayithatha so xa ndiphakathi kwabantu ndiye ndibenentloni ukuyithatha ewe

V2: I had it with me but I was scared to take it because im used to be alone at home so I don’t really have a problem taking them but im scared to take them infront of people.

V3: aha

V2: ndiye ndibenentloni ukuyithatha

V2: I get scared to take them

V3: because ubaleka lanto yokuba bakustigmatiser so ke ngoku uphele ungalenzi elaxesha bekumeluba uyithatha ngayo? Allright

V3: because you are afraid to be stigmatized, so you don’t take them on the time you are suppose to take it? alright

V2:but ke ndiye ndithi mhlawumbi nokuba mos kwakuthiwe ngu8 phaya ndiye ndithi uba ndimgqubile la8 atleast ndiyithathe ngeloxesha like kwasebusuku njalo atleast ngo9 ndiyendive ukuba ikhona lento ndingayenzanga yabo ndiyithathe ndiyibeke ngolohlobo uba ndihleli nabantu ndiyibe ibe ngathi ndifuna ukusela amanzi ndihambe ndiphume ndiyokusela amanzi, not ba ndiyithathe ndihleli ngoluhlobo nawe hahaha andikabi naso esasibindi

V2: so I just take it when ever they have gone like im suppose to take it at8pm and if I couldn’t I take it at 9pm because I sometimes feel like there is something that I did not do. If I'm with people sometimes I act as if I just need some water and I secretly take it. Not to take it in front of people, I don’t have the guts

V3: so ulinda bahambe kengoku?

V3: so you wait for them to leave?

V2: but then iyafana nalonto khange ndithathe foto ndizixelele nje uba ndizakuyithetha ngomlomo nefriends kuye kubenzima

V2: but then its like I didn’t take picures I thought I would say it like friends

V3: khanguthate foto?

V3: you didn’t take a photo?

V2: no uyazazi ifriends ziye zifunekuqonda, uyabona mos xa uhleli netshomi mos itshomi ingomntu kufuneka umtruste kufuneka uyikhethe onokuthetha into yakho kuyo. Kuye kubenzima sometimes xa ndihleli netshomi apha endlini ukuthi ndizakuyithathanjani ndiyisele

V2: no u know friend would want to know what is going on and it hard to trust friends. Sometimes I cant take my treatment when I have friends with

V3: ngelaxesha yalo.

V3: on time

V2: Kuba kaloku itshomi uhlala nayo apha ngoku ngomso uthatha ipilisi yako pambi kwetshomi ngomso kuhleliwe pha kwabani itshomi ithetha ngalanto ba he ubani unepilisi ethile ayityayo. Bazafunikuqonda ukuba yipilisi enjani le, uzakuyidescriber uba yipilisi elelihlobo omnye uzakuyazi ukuba yipilisi yantoni na le. Yabo iye ephinde ibenzima ke kum ukuba ndithi xa ndihleli netshomi ndisele ipilisi yam ndiye ndiske ndithi ngelaxesha bephuma ngalo ndiske ndiyithi laqa ke ngoku. Ezapilisi zenza umjojo ezapilisi ziyangxola xa uvula ezapilisi kukhale “khwahla khwahla”. So mna ndibenentloni bazakuthi yintoni le ingxolayo apha and ke ngoku omnye uzakufunukuqonda hayi marn yintoni le ingxolayo apo? Wenza ntoni? Zenza umjojo nyani yabo.

V2: you take your pill infront of a friend and the next thing you know they are telling other people that you are taking a pill that looks like this some people know how this pill look like they will know what its for. These pills make noise when you take them so Im afraid to take them because when they make that noise one would want to know what is that.

V3: alright ok, siyavuya ngalencazelo and usichazela kakuhle ndingekakukhokeli ngemibuzo but ubukhulu becala uyandikhokela ngemibuzo.

V3: alright ok, we are greatful for this explanation you have clearly explained everything but you were upfront with question

V2: but iyavakala nje also kukhona enye kanene apa

V2: but Its clear also there is one there thing here

V3: kufuneka sithethe ngazo ziphele ifoto

V3: we need to talk about all these photos until they are finished

V2: uyabona apha

V2: you see here

V3 foto namba 11

V3: photo number11

V2: yabona apha bendisuka ekhaya apha

V2: you see here I was comimg from my home

V3 ubuhamba ngenyawu ebusuku?

V3: you were walking at night?

V2: bendihamba ngenyawu ewe, bendizixelele ukuba ndizolala but kwakho langqondo yentoba bendizixelele andizolala apha and ikhona into endingayiphethanga ekufuneka ndiyisebenzisile. Ndaphuma I think its was 7:15 ndileqa u8pm kwelicala.

V2: I was walking yes, I had told myself that I was going to sleep there but I couldnot because I didn’t have my treatment with me. I went out I think it was about 7:15 rushing for 8pm this side

V3: apha kwelicala ixesha lokuthatha itreatment ngubani ngu8pm?

V3: what is the time you take your treatment is it 8pm?

V2: ngu8pm ndileqa lona, ndazixelela ukuba izakundikhumbuza bekungolwesingaphi na? ngolwesine

V2: its 8pm that I ws rushing for so I told myself its going to remind me what day was it? Thursday

V3: ngolwesine last week nhe?

V3: Thursday last week?

V2 ewe ndaqonda ukuthi izakundikhumbuza

V2: yes I thought it would remind me

V3: mawungabi salala?

V3: so you didn’t sleep over?

V2: mandingabi salala izakundikhumbuza ndaqonda mandifote lendlela izakundikhumbuza bekutheni zendidelayer for itreatment yam ngelaxesha because ndaye ndafika late sebethile la8pm

V2: so I thought I should take a picture of this road it will remind me why I delayed to take my treatment intime because I got here after 8pm

V3: la8pm ok

V3: 8pm ok

V2: but ke ndaqonda sendikhona mandizithathe kunoba ndiziyeke ndingaziseli.

V2: but I thought I must take it instead of not taking it at all

V3: oh alright

V2: apha bendifuna andiyazi ayivelanga kakuhle bekunetha apha

V2: here I wanted im not sure its not clear

V1: photo 12

V2: bekunetha apha kodwa ndizamile ukwenza lamathontsi

V2: What was this but I tried to make the rain drops

V3: ok

V2: ndizamile ukufota lamathontsi apha bekunetha apha ndandizixelele phofu ukuba ndibawela ukuya eclinic ndabethwa yimvula phofu ke actually ndindingazokusa mna ndandizakusa umntana. Yeyalast week bekunini ngokuya bekunetha sivuke kunetha?

V2: I tried to take a picture of the rain drops, here it was raining and I wanted to go to the clinic acutally I needed to take mychild to the clinic but I couldn’t because of the rain. Its last weeks one when was it when it was raining

V3: bekuFriday ngokuya bekududuma

V3: it was Friday when we had thurder

V2: bekufriday ndandizokuya eclinic apha so laweather yangalamini yabangela ukuba ndingakwazi ukuya eclinic indivale.

V2: it was Friday and I wanted to go to the clinic but the weather was against that

V3: all in all yakwenza uzive njani because I believe ibikhona into ubufuna ukuyiyela eclinic?

V3: al in all how did it make you feel because I believe there is something that you wanted from the clinic

V2: ndiye ndayiUnderstander ukuba yiweather akukho nto bendizakuyithini but ke ndazixelela into yokuba the following day ndizakukwazi

V2: I just understood that ther was nothing I could have done it was the weather but I told myself I will go the following day

V3: uzakukwazi ok

V3: did you manage ok?

V2: ok also Friday

V2: ok also Friday

V3: kukho enyifoto?

V3: is there another photo?

V1: photo12

V3: kukho enye ifoto12 lo

V3: there is photo 12

V2: Friday kange ndikwazi at all ndithi ba ndiyayithatha nhe because ndiyifote Sat yenzeke ubusukuFriday ndayifota Sat, because ndandineproblem neboyfriend yam. So kwacacuba siyalwa kengoku so uyayibona ledram, salwa kanye xa kufika elaxesha lam loba nandithathe itreatment. So andakwazi andithi siyalwa apha phandle yimoto le wafuna ukundigila ngalemoto. Yabo kuba ke sasixabana ngeproblems but lonto yayivala yabangela ukuba ndiligqube ndingalifumani. So nam ngenxa yomsindo nam ndayiyeka lonto pha. So ndiye ndayishooter Sat morning. But nayo khange indivise kamnandi ukuab la1day ndingayiseli ipilisi yam. Like andingomntu andifuni kuphosa because kaloku andiyazi ngosuku endiyiphose ngayo kuzokwenzeka ntoni.

V2: Friday I could take it at all I took it Sat but it happened on Friday. I dah a problem with myboyfriend, we had a fight so you see the dram. We fought on the time that I was suppose to take my treatment so I couldn’t take it. This is a car he wanted to run me over with. I took it as a reminder that 1day I didn’t take my treatment so that is the day I didnt take my pill. Im not a person who doesn’t take their pills because I don’t know what will happen for not taking mypill even for a day.

V3: hmm alright

V1: is that it? Ok

Graonning noise

V3: ubuthatha ifoto mos usogqiba ucime kangoko? So ibattery ayikho flat

V3: you were taking pictures and switch the camera off, so the battery is not flat?

V2: hayi ayikhoflat, jonga ibattery iyavela apha uyabona

V2: no its not flat, look you can see the battery here

V3: ok bendifuna uchecker nje

V3: ok I wanted to check

V1: you know about cameras its good

Laughter

V3: ok so sasitshilo mos uba xa sifika pha sizakuthetha ngazo ezifoto sakugqiba kengoku sizame ukuzithatha for ukuba siyozinatsika phaya eofisini then kengoku ndizakhupha lememory card ilapha ndifake enye le izakuhamba nomama uCarty aye nayo eofisini.

V3: so we had said mos that when we come back we are going to talk about the pictures then take them to the office. Im going to take the memory card out and put another one. This one mama Carty is going to take to the office.

V1: that’s it check if it’s the right number 4

V3: so sifaka lememory card intsha then kuba singafuni iphazamiseke lento yalememory card yako siye sibeke nesticker and also ukuba singafane siphazamele kuyo siyivule uyabona sinale bar code so sinale bar code sasithethe ngayo

V3: so we are putting the new memory card because we don’t want this memory card thing to disrupt you we put a sticker and also if by mustake we open it we will see this barcode. We have this barcode that we talked about

V1: are you ok with that?

V2: oh im cool

V3: otherwise ungasibuza uba unayo eminye imibuzo ngokusebenzisa lecamera

V3: otherwise you can ask any questions about how to use the cemera

V2: ndandiphendulekile ngalamini

V2: I was answered that day

V3: wawuphendulekile akukhonto ikuxakayo naxa uthatha icamera uyayazi umntu kufuneka umbuze ukuba ungamthatha na ifoto.

V3: you were answered so you don’t have problems when taking pictures, you know you have to ask for permission before you take someone picture

V1: so you are ok everything is ok?

V3: so ukuba kukho into ufuna ukuyibuza inumber yam unayo ikhona apho

V3: so if you need to ask me anything you have my number its there

V2: ewe

V2: yes

V3: noba undisendele ucall back or missed call

V3: even if you send me call back or missed call
