# Supplementary material for: “If I don’t take my treatment, I will die and who will take care of my child?”: An investigation into an inclusive community-led approach to addressing the barriers to HIV treatment adherence by postpartum women living with HIV
Source: PLoS One. 2023 Apr 20;18(4):e0271294. doi: 10.1371/journal.pone.0271294 (PMC10118130; doi:10.1371/journal.pone.0271294)
Supplement: S6 File — (ZIP) [file pone.0271294.s006.zip › 42-18 3rd.docx]

4^th^ recording

V1: yi21 kaMarch idate namhlanje 2018 ucamera ngucamera namba 42

V1: its the 21 of March 2018 today camera number 42

V2: ngu 42 okanye ngu24

V2: its 42 or 24

V1: ngu42/18 so sizakuthetha ngopicture namba 1

V1: its 42/18 so we are going to talk about number1

V2: picture namba 1 apha sinendlela uyayibona le ndlela? Lendlela indireminder into yokuba kukho abantu abanelenchaphazeleko, kufumaniseke ukuba umntu une18 okanye une20 yrs ephila nayo and akukho nto itshoyo ukuba unjena. Kemna indireminder ukuba mna ndiyafuna ukufana nalendlela kuba lendlela inde and nababantu baqale pantsi banyuka nayo le ndlela and basasucceda nanamhlanje basaphila nanamhlanje, so xa ndinokuqisekisa nam ndingangumzekelo walendlela. Like nam ndiphile ubomi ubude, kukho ezi andiyazi ezi zishiyeke njani zeziya zazikula memory card yokuqala. Apha sinefoto apha

V2: Picture number1 here is a path you see this path!? This path reminds me that there are people who are infected, u found out its been 18 to 20yrs leaving with it and there is nothing that says they are infected. It reminds me that if I want to be like them I should be consistent in taking my medication to be like them if I want to leave o long life. Im not sure how were these left behind they are from the first memory card. Here we have a photo.

V1: upicture namba2 yifoto yomntu oyindonda le

V1: picture number2 it’s a man’s picture

V2: picture namba 2 some people abakwazi ukuya eziklinic uyokuthatha amachiza abo because of there is a lot of drugs especially ezidolophini ufumanise xa umntu ethi uyizithathela amachiza ache eklinik ayaxuthwa becayuse kaloku ayatshaywa. So lepicture ikhombisa ukuba kukho ababantu basebenzisa lepilisi

V2: picture number 2 some people cant go to the clinic to get their medication as they is a lot of drug use especially in towns. People get robbed their medications due to this drug use even if you send someone else they also get robbed these pills. So this picture shows there are people who use these pills as a drug

V1: as ukuyitshaya

V1: for smoking

V2: ewe and nayo ke ngoku kuthwa iyatshaywa so ufamanise abantu abaninzi bayoyika ukuyoyithatha itreatment yabo because of kaloku kukho ababantu babaxuutha apha ezindleleni. Ibikhe yavela kakhulu apa eTV kuleminyaka idlulileyo kwacacukuba abntu bayathathwa bayityiselwa eklinik. Athi umntu noba unikele umntu uba makayokumthathela kufumaniseke ukuba nalomntu ufika ngaphandle kwayo endlini ixuthiwe. So mna ngokunokwam indibangele ukuba ndihlale kulendawo ndikuyo kungabikhomntu uyaziyo ukuba ndinalento because akukhomntu uyaziyo xa lepilisi ininzi kangaka kulelali abanakuza ukuzothatha ezi.

V2: yes even it, its said they smoke it and you find people afraid to go get their treatment because there are people who rob them their medication. It appeared on TV as well in the past years. People had to go to the clinic to take their pills, sometimes even if you send someone else they were robbed these pills. As for me im fine were I am where no one knows that I have this thing because no one knows when they will come to this village for this pill.

V1: ok now that bengasazi istatus sakho? Oh ndiyayiva ipoint yakho.

V1: Ok now that they don’t know your status? Oh I hear your point.

V1: ok

V2: picture namba 3 apha bendikunde qha ayibonakali ngularonder uwileyo lowa.

V2: picture number3 here I was far its not clear its a rounder that has fallen there.

V1: yindlu ewileyo?

V1: it’s a fallen house?

V2: so ndiyendacinga ukuba ndinomntana and ndinomtana nje ingase mhla ke indithatha indibeka phantsi ndibe nento endiyenzileyo like ikhaya lakhe umntanam ndiyazi ukuba unalo ikhaya lakhe. Kuba kumaxesha akhoyo uye ufumanise ukuba abantu befamily bayasweleka ikhaya liyawa. So ndanequestion mark xa ndingenozama mna ikhaya for umntanam kulemo mna ndikuyo, uyakushiyeka esithini yena.

V2: so I was thinking I have a child and I wish that one day when it takes me down I wish that I would have made her a home and know she has a home. Because in this day and age you find that families die and a home falls so I had a question that if I cant try and make a home for mychild where would I leave her in this condition that im in.

V1: and we all know ukuthi isgala siyabangwa ngumntu.

V1: and we all know we will all die

V2: so iye yandivisa kabuhlungu landlu iwileyo phayana because ndaye ndacinga ukuba owam umntana uyakushiyeka esithini xa ikhaya libonakala linjeya. Xa mna ndingenakuzimisela kumachiza am.

V2: so that really hurt me to see a home like that because I thought what mychild would be if I don’t take my treatment seriously.

V1: ukuzolungiselela yena, picture namba4

V1: to prepare for her, picture number4

V2: picture namba 4 yiklinik le imbangi yokuba ndifote leklinic kukho abantu abangazaziyo istatus zabo, kukho abazaziyo abantu bayoyika ukuya kwiklinik because ufumanisa if uyile uyotesta mhlawumbi ufumanise uHIV mos iclinic yisecret yakho, andisiboni isidingo sento ubuyiyele eclinic ufumanise ukuba uthethwa ngaphandle. Ufumanise ukuba abanye abantu bayazishiya iclinic zabo kwivillages zabo, baya kwiclinic abangaziwayo kuzo like abantu bayoyika ukuya kule yakhe kulelali ahlala kuyo mhlawumbi make kuke aye kwaNdlambe woyika into ba lento ebeyoyenza phaya izakuthethwa ngaphandle yaziwe yilali yonke. Iphinde indivise kabuhlungu lanto, bakhona abantu abatshoyo apha kum ngokuncokola nje ngokuba sincokola nje athi hayi mna asoze ndiye eclinic ngoba eclinc kukho ulwimi.

V2: picture number4 it’s a clinic the reason I took a picture of a clinic there are people who don’t know their status, and there are those that know they are afraid to go to the clinic. If they go to test and found out they are HIV mos it’s a secret when you go to the clinic. Some people go to other villages as they don’t want people in their village to know what wrong with them like if someone from this village goes to Ndlambe clinic instead of this one here because they are afraid they will hear their business in the village.

V1: abe engazazi nestatus sakhe

V1: not knowing their status

V2: abe engazazi nestatus sakhe ufumanise ngoku umntu uyayishiya ivillage yakhe uya kwivillage engaphandle

V2: not knowing their status and people go to other villages that are outside

V1: noba akekho right akaphilanga?

V1: even if they are not well?

V2: noba akaphilanga kuba kaloku woyika into yokuba ndizakuhletywa phaya eclinic.

V2: even if they are not well because of the gossip at the clinic

V1: okungabikho kwePrivacy sistaff esi as onurse or ziCHW’s?

V1: who doesn’t have privacy is it the nurses or is it CHW’s?

V2: like mna andikayifumani loproblem but xa ndisiva ubukhulu becala mos amanurse ayonwaba nawaphina amanurse ufumanise apha ekonwabeni kwabo bayathetha also namavolontiya uthi usiza uya eklinik sele usaziwa ukuba uzele ntoni. That’s why abanye abantu besoyika but mna andikabi nayo loproblem. Like andikayiva mna ngam but abanye abantu bayakhala ba kwikliniks zethu kukho into enje

V2: like I haven’t had that problem but when I hear it the nurses have fun so when they are having fun they talk and even the volunteers as well. They talk about why are you at the clinic that’s why some people are afraid but I haven’t had that problem.

V1: ivela ithi saa ngokubuza ukuba uthethe confidential nonurse?

V1: it gets talked about even though you talked about it in confidence of the nurse?

V2: which is ingabanikisi mdla abanye abantu athi umntu ngoku efuna ukuya uyozitesta okanye lonto uyoyika ngoba kaloku uzakuyiva ithethwa ngaphandle

V2: which makes them not want to go even if they want to test or for something else because they are going to hear about it outside.

V1: which is true

V2: yabo apha picture namba 5

V2: you see photo number5

V1: picture namba 5

V1: picture number5

V2: liholo eli labahlali like imbangi yokuba ndifote eliholo ndiye ndacinga ukuba kukho abantu abangawaziyo umehluko phakathi koHIV+ noba neAIDS. Like ngokunokokwam ingaske kubekho istudies like nhe kubekho amaxesha kuzofika abantu bezobachazela abantu ngeHIV babachazele nangeAIDS babacacisele yintoni iAIDS yintoni iHIV. Also bakhuthaz abantu ukuba mabatye itreatment yabo.

V2: this a community hall the reason I took this picture is that I thought there are people who don’t know the difference between HIV and AIDS. If it was up to me I wish there could be studies where people would be explained to the public the difference between HIV and AIDS. and to encourage those that are positive to take tier treatment.

V1: sort of campaign?

V1: like a campaign?

V2: ewe sort of icampaign, like babakhuthaze abantu aba sele beyitya baqhubeke bayitye because mna ndinalafear yentoba mhlawumbi ngenye imini liyakuze livele ichiza lento inyangeke. Umzekele iTB yayinganyangeki ngo iyanyangeka so mhlawumbi nayo kuzakuhamba kuhambe ichiza libekhona. Ingaske kufike abantu abazakuhlelinje baya encourager like bangenzi icampaign yabantu abaHIV bodwa, ibe ngumntu wonke apho kungazocaca khona ukuba ngubani oHIV ngubani ongekho HIV. Like kwenziwe istudy circles wonke umntu afundiswe ngesisifo esisifo yinto

V2: yes like a campaign, like encourage those are already taking treatment to continue because I fear that one day this thing will be cured for example TB it also didn’t have a cure for a while but now its curable. I wish we could get people that would encourage all the time and do campaign not only for positive people but for everyone and do study cycles teaching what is this disease.

V1: and sinobungozi kangakananina

V1: and how dangerous is it

V2: ewe sinobungozi kangakananina because abanye uye ufunaseke ukuba uyaya eklinik ayo testa angayamkeli. Like umntu achazelwe ukuba yamkele and ukuyamkela kwako uzakuphila ubomi obude utye itreatment yakho.

V2: yes how dangerous is it, because some gets tested and don’t except it. Like people be told to except it and take their medication to leave a long life

V1: omnye athi setestile angafuni kutya itreatment

V1: some after testing and not want to take treatment

V2: also baencourage nangefuture yabo, umntu acinge ngefuture yakhe ukuba ifuture yakhe xa ungazoyitya letreatment kuzakwenzaka ntoni. That’s why difote leholo ngaske kubekho istudy cycles okanye laCampaign yakwaHealth apo khona kuzakufundiswa abantu ngalento nzulu ngayo. Because abanye abantu abayazi omnye uyokuthuka mhlawumbi athi uneAIDS akayizi Iaids yintoni.

V2: and also get encouragement about their future so people think about their future and how will happen to them if they don’t take their treatment. That’s why I took a photo of this hall I wish there could be study cycles or Dept of Health campaign where they can teach people more about it. Because some people say you have AIDS they don’t even know what AIDS is.

V1: babeliver ukuba sisifo esibad ukuba unaso uzakufa

V1: they believe it’s a bad disease and if you have it you are going to die

V2: that why ndiye ndafota leholo, apha ke bendihluphekile ke apha

V2: that why I took a picture of the hall here I was hurt

V1: picture namba6

V1: picture number6

V2: picture namba 6 bendihluphekile apha

V2: picture number6 I was hurt here

V1: yintoni le ifotwe apha?

V1: what picture is this?

V2: zihagu ezi aphendlini ndinehagu but ezihagu ayizozam, zezasefamilini so ezihagu bezilahlekile khangebehle ubuthongo ke kum kuse ndithe because ndafika ebusuku apha ndazifumana ngengomso apha pha ngaseholweni. Kusebusuku apha and istress sam sosokuba ubusela buninzi apha elalini, xa ndingazokuzikhathelela ezihagu because ekugqibeleni ibakhona into ezindingenisela yona ezihagu. So ndandihluphekile azange ndiyiphathe at all kuba ndafika ebusuku endlini ngo9pm ndikhangela ezihagu. Ndizakuthi bezihlukene njani neyadi kumntu wazo kuba ekugqibeleni ziyandinceda xa kuthengisiwe ndiyafumana into encinci so that is why ingathi ndiyazikhathelela kakhulu. so ndandihlupheke kakhulu apha zange ndiphatheke kakuhle kuba kaloku ndabhuda elaxesha lam ndafika ebusuku ngaboo

V2: its pigs here I have pigs that I look after for a family member. I could sleep when I couldn’t find them because I got home late at night I only found them the next day next to the hall. It’s at night here and my stress it that there is a lot of theft here in the village. I had to care for them as I get something when they are sold. So I was hurt and didn’t take them as I got home at 9pm what was I going to say to the owner.

V1: so awayitya

V1: so you didn’t take it?

V2: ewe andayithatha, nezibhokwe nazo same thing azabuya

V2: yes I dint take it even the goat dint come home

V1: picture namba7

V1: picture number7

V2: azibuyi nazo ezibhokhwe nazo zindenzela enye into because ibangathi akukhathali xa ugcine into yomntu same day yazo le

V2: these goat didn’t come home as well and there other thing is that when you look after someone else’s things and they get lost its as if you don’t care.

V1: more specially owefemeli

V1: more especially family

V2: yisame day yazo nezahagu

V2: it’s the same day with those pigs

V1: kuba kwakwenzekile akwabikho endlini?

V1: because it happened that you were not at home?

V2: apha

V2: here

V1: picture namba 8

V1: picture number8

V2: apha yindlu le mos xa usendlini usoyika mhlawumbi, like abanye abantu abakwazi mhlawumbi ngumntu o1 wefamily okwaziyo ukuba unjena. So uye unqonde ukuba xa kufike abantu endlini uye woyike ukuthatha elachiza lako pambi kwabantu also kufuneka ube neprivacy yazo apho uzakuzibeka khona zingabonwa because abanye abantu bayazazi ukuba zezantoni ezapilisi and ziyabhalwa ukuba yekabani le so uye woyike kengoku kufumaniseke ukuba ngase singabonwa ngumntu zibekwiprivacy yako. Naxa uzifihla woyika ukuba uzakuthina lomntu xa ezibina. Uyabo

V2: here is a house maybe when you are at home and your afraid like maybe only one person in the family that know about what you live with. So when there are other people you are afraid to take your treatment in front of them also you must find a private place to put them so that no one sees them. Because written on them are who they are for and some people know what are they for. You are afraid what will they say if they find out.

V1: but ke nje ilizwi lengcebisi don’t you think ukuthatha noba yicontainer kumamakho ingakunceda? Xa ungekabikhocomfortable

V1: but a word of advice, don’t you think maybe using a different container even if its your mothers it can help. When you are still uncomfortable

V2: mhlawumbi ingandinceda

V2: maybe it can help

V1: uzidibanise kwinto eyi1

V1: mix them in one container

V2: mhlawumbi ingandinceda because labhotile

V2: maybe it can help because that container

V1: because umntu as ixesha lihamba nguye oyaziyo so long ungekabicomfotable ukupreventa lanto ixesha lizakuhamba ungazityanga ipilisi kuba usonqena ukuvula lacontainer. Kuba attime sinefamily nje ngeGood Friday le izayo ngoku

V1: because you are the one who knows as the time goes how comfortable are you with telling other people. To prevent not taking your treatment use a container because its times for families now like its Good Fridays very soon.

V2: uyayibona le?

V2: do you see this one?

V1: picture namba 9

V1: picture number9

V2: ngu9 lixhegokazi eli umqweno wam nenzondelelo yam ombangi yokubandifote elixhegokazi, dithe xa ndibona elixhegokazi ndacinga ukuba xa ndinoqinisela ndizimisela ndingafika mna kwelixhegokazi. But kuba lona elixhegokazi lakudala zazingekho ezazinto uyandiEncourager. Qho xa ndidibana nalacontainer yam iyandikhuthaza ukuba nam ndizakufika kulapicture. And ndimbone umntam xa ukhula esenza izinto because elixhegokazi libabukele abantwana balo besenza izinto phambi kwalo. So xa ndibona elaxhegokazi nam ndiye ndiencourageke ndifumanise ukuba ndingalenza nam elixesha lenziwe lelixhegokazi.

V2: its9 its an old woman my wish is too live as long as this woman watch mychildren grow old and prosper in life. So when I see this old woman I get encouraged to take my treatment as to leave as old as her.

V1: injalo injalo, qha ixhomekeke kubasireose kwako.

V1: its like that and its up to your seriousness

V2: ixhomekeke kubasereuse kwako apha liliwa eli,

V2: its up to my seriousness, here is a cliff

V1: picture namba

V1: picture number

V2: 10 ifear yam kweliliwa xa ndinongazimiseli mna kuleproblem yam, umntanam uyakuhoywa ngubani uzakumgada xa ndingekhoyo agafikeleli azodlala kweliliwa.

V2: 10 my fear about that cliff is that when I don’t concentrate on my problem, who would look after mybaby and guide her not to go towards the cliff.

V1: And we all know ezantsi kwelaliwa ngumlambo

V1: and we all know under nearth that cliff there is a river.

V2: ingaske ke mna ndizimisele ndizokwazi ukuthi emntaneni wam hayi sukuya phaya ngoba omnye akazokwazi ukuba nomonde wokumane esithi hayi qho because ndiyayazi abantwana bangoku abamfuni uhayi. And akukhomntu uzafuna ukukadwa ngomntana womnye umntu. Ndibe mna ndingakhange ndizimisele ndabona ukuba mna mandimshiye apha emhlabeni lomntana so ndabona ukuba mandifote eliliwa.

V2: I wish I could concentrate on my treatment so that I can tell my child that its dangerous to go near that cliff because other people wont have time to say no all the time. I know kids today don’t want no! and no one want to be bothered by someone else’s child. Whilst I didn’t take my treatment seriously and died left mychild behind so I thought I should take a picture of this cliff.

V1: kuba usazi ukuba linobungozi?

V1: because you know its danger?

V2: kuba ndisazi ukuba linobungozi so eliliwa lindicingisa uba xa ndingekhoyo mnaumntana wam angaza apha azodlala apha.

V2: because I know the danger of this cliff its make me think that when I don’t take care of myself and be gone mychild might come here and play

V1: and nguwe wedwa onomxelela istory ezenzekayo kwelaliwa nalamlambo.

V1: and you are the only person that can tell her stories about that cliff and the river.

V2: yabona

V2: you see

V1: picture namba 11

V1: picture number11

V2: uyabona apha ndibafote bedlala apha ecreche imbangi yokuba ndibafote apha kukuva kabuhlungu kuba kukho abantwana abangaka okanye abangaphantsi kwaba abashiywa ngabazali babo because of umntu angayamkeli untoyokuba unjena and angafuni ukutya itreatment. So mna ndizibuze umbuzo mna uba xa ndizakushiya umnta wam ongaka ngubani ozakumhoya.

V2: you see here I took them playing at creche the reason I did that is the hurt I feel when I’m watching them because someone them their mother’s died at that age and some younger than that because they didn’t except and took their treatment. So I ask myself if I leave my small child who will take care of her.

V1: amkokose nje ngawe?

V1: gently take care of her like you do?

V2: amkokose nje ngam, ngubani ozathi xa ekhulile ambalisele ukuba kwakusenzeka ntoni kudala ambalisele into zakudala. And andibaweli umntan wam akhule engamazi umama wakhe, because abantwana abangaka bakhula bengabazi abazali babo. And ufumaniseke ngoku nakwezindawo bahlal kuzo abaphethekanga kakuhle.

V2: gently take care of her like me, who will tell her stories when she is grown, and I don’t like it for mychild to grow up without knowing her mother because kids this age grow up without know their parents. And u find out that even in the homes they stay in they are not treated well.

V1: akhule eneAnger

V1: grow up with anger

V2: akhule eneAnger ezibuza ukuba kwakutheni umamakhe za mshiye kuba akayazi ukuba kwakutheni ze amshiye umamakhe.

V2: grow up with anger and ask themselves why did their mother leave them because they don’t know what really happened for her to leave

V1: and I believe sishiya inkedama zangabom xa likhona ichiza lokuyithomalalisa lento.

V1: and I believe we leave orphans carelessly if there is a treatment for it.

V2: picture namba 12 ngumzekelo lo ophilayo lomzekelo uphilayo wayenomama but umamakhe akasekho, so lonto yandifundisa ukuba aphefemelini uyofumaniseka ukuba xa umntu engenamzali uphatheka kabuhlungu just like lomzekelo lo cause lomzekelo lo wabona ukuba makathathe inyawo aye loyise

V2: picture number12 this is a living example of a child that had a mother but know she is motherless. So that taught me in the family when a child doesn’t have a mother he/she gets treated badly just like this living example so this one decided to go to her father’s family

V1: kulotata

V1: father’s side

V2: ngoba kaloku akaphethekanga kakuhle kulomama uzizithuko. Akukho gama ke ndilicaphhukela athi umntu xa engazimiselanga kwitreatment yakhe athi akutshona athi umntana xa ufuna into kuthiwe nakuya ezantsi kobuhlanti.

V2: because she was not treat well at her mother’s she was called names, there is a word that I hate like when a child wants something she is referred to the outside of the kraal.

V1: emangcwabeni

V1: at the cemetery

V2: so mna indireminder ukuba xa ndingenozimisela

V2: so it reminds me if I wouldn’t really take my treatment

V1: which is uzakubanjena nowako

V1: which is yours will be like that

V2: uzakubanjena nowam

V2: mine will be like that

V1: ngoba yiclose family le yenza lento

V1: because it close family that is doing that

V2: also lomntana ebeneeder utando lomzali because kaloku ebelufuman utando kumamakhe but umamakhe akasekho ngoku ingaske kengoku abesemfuthweni wotando afumane utando uluzaba enough azakuthi xa ecinga isuke landawo.

V2: also this child just needed love of a parent because he got it from her mother but because her mother is no more he should get warmth of a parent and love would be enough so that he does think too much

V1: Ngoba yonke lento ayofault yakhe.

V1: because this is all not his fault

V2: yabo ngenye into endivisa kabuhlungu kuba ndinalantoba ubanangaba umamakhe wayezimisele okanye wayeyamkele mhlawumbi ngoku ngesaphila ehleli nomntanakhe. Indivisa kabuhleungu nyani yeyona indivisa kabuhlungu kuzozonke ezipicture.

V2: other thing that hurts me is that if her mother had taken her treatment she would still be alive and taking care of her child. This is the most hurting picture of them all

V1: ziphelele nhe khandiphe labhaka nana

V1: is that all? Could you please give me that backpack nana

V2: indivisa kabuhlungu nyani le ifoto kuba ndizibuza umbuzo xa kunje kuye kum kuyakubanjani?

V2: this really hurts me because really im asking myself if its like that to her how would it be to me?

V1: injalo because yiclose family le yenza lento kulomntana ingenakukwazi ukumamkela umntana nobunjani bakhe umntana because bayayazi ukuthi asiyofault yakhe, but bamstreata like dirt ngento engayofault yakhe

V1: its like that because its close family that is doing this to this child that can’t except this child the way that he is they know its not his fault but they treat him like dirt

V2: and akazenzanga ekugqibeleni uyayibona lento

V2: and he didn’t make himself you see

V1: tu

V2: yilento ndithi ukuba wayeyamkele okanye ngokuya kwakusuthiwa makaqiniseke etreatmentini ngesaphila.

V2: that is why I say if he had excepted and continued taking treatment she would stil be alive.

V1: ubani umama?

V1: who the mother?

V2: umamakhe

V2: his mother

V1: alright zimpendulo ezingaphendulekiyo ezo so ungazi ba uba ephila ayibone lento yenzekayo. So ngoku sizakuthatha lememory card indala sifake entsha jonga ezi ipicture kufuneka zihambile.

V1: alright these are unanswered questions that you wish if she was still alive she would see what going on. So now we are going to take the old memory card and put a new one look these picture must go
