# Supplementary material for: “If I don’t take my treatment, I will die and who will take care of my child?”: An investigation into an inclusive community-led approach to addressing the barriers to HIV treatment adherence by postpartum women living with HIV
Source: PLoS One. 2023 Apr 20;18(4):e0271294. doi: 10.1371/journal.pone.0271294 (PMC10118130; doi:10.1371/journal.pone.0271294)
Supplement: S7 File — (DOC) [file pone.0271294.s007.doc]

1. Sisanyamezele

We are still surviving

1. Okay sinopicture number 1 apha nhe?

Okay we have picture number 1 here isn’t?

1. Okay idate yanamhlanje yintoni yi26 kaMatshi 2000 n…2018… sine…sino picture number 1

Okay the date today is the 26 of March 2000 and… 2018… we are… we have picture number 1

1. Picture number 1 upicture number 1 undikhumbuza into yokuba, kukho abantwana…umzekelo bendibukele indaba izolo

Pictuture number 1, picture number 1 reminds me of the, there are children…for an example I was watching news yesterday

1. Um u

yes

1. Kukho abazali bebekhala phaya

There were crying parents that were there

1. Um u

yes

1. Bekukho abantwana babo abatestiweyo without ipermission yabo waye esithi lomama umntwana wakhe utestiwe waphuma iresults zithi upositive kungakhangwe kuinformwe phofu umzali into kuba sizotesta umntwana wakho uvele nje wasayinswa of which besikhe sanayo nathi apha iproblem enjalo indayazi iphelelephi na umntana wafika kowabo esithi ilantuka

There were children that were taken for an HIV test without the permission of their parents, this particular parents was say her child was tested and the results came out saying the child is positive, any way this have happened without the consultation of the parent that, that we will be testing your child. The child was given forms to sign, in fact even us here we had a problem which was like that before I don’t know how it ended when a child got home and said this…

1. Utestiwe

He or she is tested

1. Utestiwe

He or she tested

1. Um u

yes

1. Andiyazi iphelelephi na iyeyandivisa kabuhlungu lo nto ngoba lamzali mhlawumbi khange amxelele umntwana wakhe mhlawumbi wasuleleka lamntana kumzali wakhe so umzali wakhe khange abenandlela yokumxelela kakuhle

I don’t know how it have ended, that makes me bad because maybe that parent did tell her or his child that he or she got the disease through her , so the parent had no right time to tell the child on clear terms.

1. Oonesi baye bamchazela ukuba upositive lomntwana okanye

Did the nurses told that child she or he is positive or not

1. No, la mntana uchazelwe qha ba pha ukuba ukhe wabona imigca emibini upositive uba umnye lomnca awukho positive xabuzwa kangoku phayana kwabanye abantwana uthi omnye umntwana mna ndibuziwe ukba umama uyayazi nalento

No, that child was told there only this, if you have seen two lines there, you must know that you are positive, if the line is one you are not positive, when the other child amongst the others was ask whether his or her mother knows about this…

1. Um

Yes

1. Ukuba sizotestwa apha kwathiwa hayi akayazi sayina nantsi ifomu abantwana bakwiage group benza ugrade 7

If they are there for HIV test, they were told no they don’t know, here is a form just sign, these children are in the same age group, they are doing grade 7

1. Okay
2. And lento ayenzekanga kude yenzeke apha eBhayi benza oograde 7 basebancinci abantwana batestiweyo pha so indivisa kabuhlungu mna la nto

And this thing did happened very far, it had happened here in Port Elizabeth (eBhayi), they are doing grade 7, these children that were tested there are still young that thing makes me feel a pity

1. Um

Yes

1. Ngoba ndine question mark ethi xa lamntu etesta lamntana emxelela lamntana ukuba uHIV positive la mntana xanothatha iipilisi pha azibulale

Because I have this question, when the person who is testing that child tells that child that he or she is HIV positive if that child can overdose himself or herself with tablets and kills himself or herself

1. Um u

Yes

1. Okanye azifake emotweni bakaloku kuthiwa uHIV positive also lamntwana khange akhansilishwe nokukhansilishwa…. Uvele wachazelwa akayazi nokuyazi nokuba yintoi le nto

Or throws himself or herself on a moving car, because the HIV news and that was never been caunceld in the first place… she or he was just told about this and she or he doesn’t know a thing about this

1. Which is akayazi kakuhle lemeko the only thing ayaziyo yeyokuba uzokufa qha

Which is, she or he is not experienced about the situation, the only thing she or he is aware of is that she or he is going die

1. So iye yandivisa kabuhlungu kakhulu lo nto leyo and ngabantwana abakuleage group

So that thing made me to feel very bad and those are the children which are at this age group

1. And kangoku akukhonto indenza iconcern njengokuba akhomzali ngalaxesha

And the most thing that makes me concern is that there no parent

1. Akhomzali ngelaxesha umntana yena ufika ekhaya esithi nditestiwe kuthiwa ndineAids

There were no parent by that time, a child comes home saying, I was tested and I’m told I have AIDS

1. Um….Yabona nendlela ayibiza ngayo ithi lonto akayiunderstandi , kwalonto ukuba akayazi idifference between iAids neHIV…apha

Shu! You can even notice the way she or he puts it that she or he doesn’t understand that, even the way she or he cannot differentiate between the AIDS and the HIV… here

1. Yipicture number 2 apha…iye yandivisa kabuhlungu le nto lonto ke yabangela ukubana mandiqine because umtana wam usemncinci

This is picture number 2 here…that thing made me to feel very bad resulting on making me strong because my child is young

1. Um u

Yes

1. Ndikhe ndabona apha ezindabeni umntana I think una…I’m not sure ingathi wenza ugrade R or ugrade1 kulamacala aseTranskei otshene kwitoilet yasesikolweni usemncinci otshene kwitoilet yasesikolweni lo nto yandivisa kabuhlungu yandibangela into yokuba mna mandiqine ukukwenzela ukuba ndizoba necare for umntana wam because andiyazi into ozoshiyekanaye xandingazimiselanga kule treatment ozoshiyeka naye uzoshiyeka emtreata njani yena ingaba uzobanayo ilantuka yomjonga i…i….

Some other time I have seen here in the news, a child, I think she or he is… I’m not sure maybe he or she is doing grade R or grade 1, somewhere in those areas of the Transkei , who had drowned inside the toilet at school. She or he is still young, the child who have drown inside a school toilet, that made feel pity resulting on making strong so that I can the care for my child, because I don’t know who is going to be left to take care of her or him if don’t take my treatment regularly. I wonder if she is going to have this… what do you call it… this….this

1. Isineke

Patients

1. Isineke somjonga na

Patient of looking after her or him

1. Um

Yes

1. Uzokwazi uhlala naye phantsi amchazele ngento ewrongo neryt so ifear yam yintokuba ingasekhe mna ndi…ndi…ndiqine ukwenzela into yokuba ndimbone xa ekhulayo ndizokwazi uhlala naye ndimchazele

The one who is going to be able to sit down with her or him and tell her or him about what is wrong and right, so my fear, I wish I can be… be… be strong so that I can see her or him growing up so that I can tell her or him

1. For iguidance

For the guidance

1. ndimbonise ukuba kwenziwa njani ni na because abantwana bangoku abamfuni uhayi ekugqibeleni

show her or him that how things are done because in the first place children of nowadays they don’t want ‘no’

1. um

yes

1. so omnye umntu uzodikwa kuhleli nje uthethisa umntana womnye umntu ozenze ngamabomi phofu ongafuni ukutya itreatment yakhe wabhubha uyayibona lo nto

so somebody else can be irritated, now and then is rebuking somebody’s child, anyway the one who have sets up traps for herself and died of that

1. um u…picture number

yes…picture number

1. picture number 3 I think lo mntu una23 something kukho abantu abakuleage yalomntu abangafuniyo ukutestwa

picture number 3, I think this person is around 23, there are people who are at this age that refuses to be tested

1. um u

yes

1. boyika nto enye

they are scared one thing

1. una20 something

he is 20 something

1. um u…like usengumntu omtsha mandithi usengumntu omtsha

yes…like, he or she is a youth let me say she or he is a youth

1. um

yes

1. yabo abangafuni kuzazi izitatus zabo zinjani na because boyika intokuba umntu uzoyokutesta phaya ekliniki achazelwe pha ukubana isitatus sakhe sithini na. ngomso uzoyiva lo nto leyo eyibuzwa ngaphandle

you see those who doesn’t want to know how is their statuses because someone will go for a test to a clinic and there she or he is going to be told how is her or his status is. The next day he or she going to hear people outside the clinic talking about that.

1. um

yes

1. ba…okanye ithethwa okanye ayive ithethwa kuthiwa ubani uHIV positive because ngobukhulu becala uthi wena ikliniki yisecret yakho

that…or being told or overhear people saying so and so is HIV positive because you say a great deal a clinic is a place to keep your secret

1. um u

yes

1. but kukulamaxesha akhoyo ayiseyiyo ikliniki isecret because into oyenzayo pha ngaphakathi uye ufumanise ukuba iyathethwa pha ngaphandle

but in these days clinics are no longer places to keep secrets because the thing that are happening inside you heard them outside to everyone…

1. um

yes

1. umzekelo ukhona omnye umntu bendidibana naye kusasa namhlanje

there’s an example, someone that I met in the morning today

1. lo mntu use kule picture

the same person who is stil in this picture

1. ha a ngomnye umntu nje

no this is someone else just a person

1. okay
2. bendidibana naye apha endleleni esithi uqaqanjelwa ngumnwe ndibuza mna kutheni ungayi ekliniki nje uthi yena o hayi ndiyonqena andifuni mna ukuya kulekliniki…khange ndisibuze ke isizathu sokuba kutheni engafuni ukuya ekliniki

I met him here outside. He was saying his finger is aching then I asked him why don’t you go to the clinic. He said no I’m lazy to go that clinic. I don’t want to go to this clinic…I didn’t ask about the reason why he doesn’t to go to the clinic

1. um

yes

1. kukho abantu abanjalo ke abanye abantu bayoyika ukuyokuzitesta kwiikliniki zabo ingaskhe umntu aphume aye kwenye indawo most specially ulutsha ulutsha alufuni kuzazi izitatus zalo ingaskhe kubekho something ezokwenzwa for ulutsha bafundiswe ulutsha kakhulu intokuba umntu makahambe ayotesta ukwenzela ahlale ezazi nokubana ukwesiphina isigaba ukubana umntu omtsha ufumaneke epositive akwazi ukuhlala phantsi atye itreatment yakhe because aphinde acinge nangefuture yakhe at the end

there are people who like that, other people are scared of going for a test in their near-by clinics, it can be a good idea if someone can go to another clinic that the one in his area, most specially the youth, the youth does want to know about its status. I wish something can be organized for the youth, to be taught a lot that someone must go for a test so she can his or her status. So that if he or she tested positive she or he can sit down and take his or her treatment and think again about his or her future in the first place.

1. don’t you think ukubana nonompilo kukho apho balekisha khona bayineeda itraining

don’t you that even nurses there instances where they are lacking and they need a training as well.

1. bayayineeda itraining kwabona onompilo because isenokwenzeka ukubana ngabo aba baphuma neinformation pha ngapakathi bazokuyithetha ngaphandle

they need a training even themselves, nurses because it might happen that are them who are licking the information from the inside to the outside

1. um alryt picture number

yes alright picture number

1. picture number 4

picture number 4

1. 4 nhe

4 for real

1. Um um

yes

1. Yhaaa

yes

1. Pha ndifote ikari emileyo pha la kari ihleli ngerimu pha ndithi ndokujonga la kari indireminde intokuba themba yona bhetere kunokubana uthembe umntu

In that one I have photographed the cartwheel wagon that is standing on its rims, when I look at that cartwheel wagon it reminds me that, just trust it than trusting a person

1. Um

yes

1. Ngoba la kari xa ubuyishiye kulandawo uzophinda ubuye isekalandawo because ayikwazi ukuhamba kulandawo ngaphandleni itsalwe ngumntu into endibangela ukuba manditsho kulamaboyfriend ethu awakho honest udibana nomfana phaya umthande lo mfana ucimbe mfanaba lomfana userious ngalento kanti yena lomfana uyazazi uzokugalela ngalento azokugalela ngayo uyabona and sometimes uye ulithenge uthando ngoba kaloku…ama…amaO akhoyo awafuni ukuyisebenzisa ikhondomu and ungazomazi ukuba umntu uHIV positive okanye akekho HIV positive indivisa kabuhlungu ke lonto leyo because kukho abanye abantu abayifumene ngolohlobo kanti umntu uzomgalela ngaleHIV kuba eyigqithisa naye kuye ecinga ukubana izosuka kuye okanye esandisa ukubana ilizwe malichapazeleke ngomntu omnye

Because when you have left that cartwheel wagon in that place, you will come back and find it in the same place where you have left it before because it cannot move away from that place beside it is pulled by someone , the thin that makes say so, in these boyfriends of ours of nowadays, they are not honest, you meet a young man and fall in love with him, think in advance that this young man is serious about this affair, but in the true he is around to infect you by a thing he is here to infect you with it. You see sometimes you just have to buy love because anyway…these … these guys they don’t want to use condom, and you can’t able to predict that someone is HIV positive or is not HIV positive. That thing makes feel very bad because there are other people who got it in that fashion, in the true sense that someone have come to infect you with this AIDS or is just passing it as well because he thinks that by doing so it will disappear from him or spreading it around so that the world can suffer because of one person.

1. Uminisha ukuthini xa usithi ulithenge uthando

What do you mean when you say to buy love

1. Uthando ulithenga njani, iboyfriend yakho uyayithanda xa usithi kwiboyfriend yakho ndicela sisebenzise ikhondomu ingafuni caba uzokwahlukana nayo ngenxa yalonto kulapho uchaphazeleka khona ngenxa yokuba umazi nokuba unayo okanye akanayo and abafana abakhoyo abadibani nekliniki abafuni ukuya ekliniki

How do you love, you love your boyfriend when you say to your boyfriend can we use a condom and he doesn’t want and that slightly seems to a big deal to put your affair in the margins of losing him because of that. It when you suffer because you don’t know whether he got it or not, and the young men of today doesn’t to go to the clinic, they don’t want to.

1. Um okay

Yes okay

1. Uyabona ke

Do you see

1. Which is uendaphe uyivictim ngesosizathu

Then you just end up as a victim

1. Iyaaa

yes

1. Um

yes

1. Apha

here

1. Picture number 5
2. Picture number 5, apha indikhumbuza into kuba kukho abantwana mhlawumbi abaashiwa ngabazali babo nhe kufumaniseke ukuba akukho nkathalo umntwana endlini apho aphuma khona uyathukwa yabo mhlawumbi amamakhe waye ne HIV wamshiya mhlawumbi

Picture number 5, here, This is my child I took this picture because sometimes [I ask myself] if I don’t take my pills who will look after my child so even if I’m feel I’m forced to take it for child’s sake. You see others they always swear at him or her you see maybe her mother died. She had HIV then she died…

1. Um u

yes

1. uyathukwa kufumaniseke ngoku lomntwana akanadawo yokuhlala lo mntana usetawuni

they swear at him or her, then becomes that this child has no place to stay, this child ends up being in the street

1. m m

yes

1. ucelana neemali ebantwini

she or he is begging for money

1. m m

yes

1. mhlawumbi abanye bayamthuka abanye bayambetha

maybe others are swearing at her or him or they are hitting him or her

1. m m

yes

1. yabo ngoba kaloku uzulazula pha akafumani luthando luenough pha phakathi

you see because he or she loitering here because of not getting enough love from the the inside

1. endlini

inside of his or her home

1. endlini because kaloku akana mama azomthethelela

inside the house because she or he has no mother to defend her or him

1. m m

yes

1. kulapho kangoku mna ndiye ndicinge ukuba xa kukho abantwana benza le nto bajikeleza phakathi kwezimoto

that’s when I think when there are these children who loiters among the cars

1. kwezitaxi um u

yes among these taxis

1. ufike becela iiRandi neetwo Randi pha

find them here begging for R1 or R2

1. m m

yes

1. owam umntwana uyokwenza ntoni mna mhla ndingekho because ekugqibeleni umama wam naye ngulo uzigulelayo yabo so andiyazi umntu uyolentuka kuqala phakathi kwam naye ashiyeke nabana kangoku

what my child will be doing when I’m not around because in the first place even my mother is this one who is sick you see, so I’m not sure who is going to die first between her and me, and my child be left with whom then

1. and what is worse uyintombazana

and the worst is a she

1. uyintombazana noba yintombazana,

and she is a she

1. um u

yes

1. uzophinda yena abe yivictim xa ejikeleza phakathi kwezimoto ezi abe yivictim yoreyitywa aphinde abe yivictim yochaphazeleko le HIV uyabona

and she will end up aging being a victim when she loiters among these cars, she can be a victim of rape and again be a victim of HIV you see

1. picture number 6
2. uyabona apha uye, umntu osebenzisa itreatment nhe kufumaniske ukuba xa nihleli niloluhlobo

do you see here, you, when someone who uses a treatment you know, to find out when you are sitting around in this manner

1. um

yes

1. kufikelele elaaxesha lakho

you arrive in your time for your treatment

1. um u

yes

1. kuye kubenzima ukuba mawuthathe iipilisi zakho

it becomes so difficult to take your pills

1. um u

yes

1. because uye ushiyeke unequestion mark yento uba bazoshiyeka bezibuza ukuba ziipilisi zantoni ezi ndizityayo

because you will left with questions that what they will be asking themselves about these pills, I wonder she takes these pills for what for

1. um u

yes

1. okanye uve kuthethwa ngaphandle

or people talking about that around the town

1. um

yes

1. mos iitshomi ayizizo ezothenjwa ekugqibeleni

any way you cannot trust friends

1. um

yes

1. uve kuthethwa ngaphandle ebesitya iipilisi ngexesha elithile. Abayazi (yo) ke bayayazi ezapilisi zityiwa ngalaxeshe ziipilisi zantoni. Ufumaniseke uyaspreadeka kangoku apha elalini

you will just hear people outside (clinic premises) that she or he was taking pills at that time. Those who know, they know of the pills are taken by that time are of what. Then you will find out you are a talk of the village

1. ube ungakhange udisclose

without even, of disclosing your status

1. yes ufumaniseke kangoku ukuba ujongiwe ujongelwe le nto kulapho kangoku kufumaniseke into kuba uye ufeele guilty pho mna ndifeela guilty xa ndihleli nabantu okungathi kukho into endiyenzileyo ewrongo xaku fikelele elaxesha ngoba kaloku andiyazi xandithathatha ezapilisi ngelaxesha bazoshiyeka bejongana besithini okanye xabephuma ngegeyiti bazondihleba bathini na

yes, then you’ll find out that people are looking at you because of this, it is when that you feel guilty I mean I, I feel guilty when I’m with people as if there’s something wrong that I have done, especial when the time reaches for me to take the pills, they will be left behind saying what or when they are going out of that gate they will be gossiping about me saying what

1. don’t you think iyouth iyalekisha ngeknowledge more specially apha kwiivillages apha ezilalini

Don’t you think that the youth is lacking knowledge especially here in the villages?

1. yes iyalekisha yilanto ndandithe kuqala ingase kwibekho izistudy circles

yes it is lacking the knowledge, that’s why in the beginning I said I so wish there can group studies

1. um u

yes

1. aphokhona kuzofundiswa ulutsha okanye abantu bonke belali ngeAIDS and HIV yabo

where youth can be worked shopped or where the people of the village can be work shopped about AIDS and HIV you see

1. um u

yes

1. because kucacile bakhona abangayaziyo

because it is clear that there are still those who know nothing about it

1. um u

yes

1. abangakayiqondi ukuba yintoni na lento and abayohluli uba yintoni iHIV uba yintoni iAIDS

those who don’t understand what is it and those who don’t differentiate what is HIV – what is AIDS

1. um u…where’s kukho ezinantsika italks apha eziTvini nalapha nasezikliniki kukhona italks ingathi kum isesi*stigma* ingathi uba unaso ungaskhe *uisolatwe* ukuba bekusiya ngabo ngaskhe uisolatwe nalapha phakathi kwabantu

yes… where’s there are even tv shows and talks in the clinics, I feel that the HIV/AIDS thing is still a stigma. It feels like when you are having it, it can be better if you be isolated from the rest, if they had power, to be isolated among the people

1. yes okungathi like abayazi like bafundiswa le nto kuba isulela isuleleka xa kuthweni na because kufumaniseka ukuba omnye woyika kwaijagi le yakho

yes as if like they don’t know it, that they were taught that it infects when you have done what or in which ways because it happens that someone is even scared of your jug

1. ibhekile yokusela

the tin you drink with

1. yes ibhekile le yokusela ufumaniseke ukuba omnye uyayonyanya le bhekile asela ngayo kungathi kukho lento izomosulela

yes the tin, the one to drink with, to find out someone is scared of using the same tin to drink with as if she or he will get infected

1. um u

yes

1. uyabo

you see

1. picture number 7
2. picture number 7 , mos thina kwezindawo sihlala kuzo sihlala kufuphi

picture number 7, anyway in the place we are living at, we stay close to

1. yintoni le?

what is this?

1. Lidama eli

It is a dam

1. Okay
2. Sihlala kufuphi nemilambo namadama

We stay close to the rivers and dams

1. Um

Yes

1. And amadama alapha awakhomancinci makhulu so eli dama lindikhumbuza into kuba, xa mna ndingeno…mos mna ndinomntana nhe as usual nhe

And our dams here are not small they are big, so this dam reminds me of, if I cannot…anyway I have a child as usual you know

1. Um

Yes

1. So xa mna ndizoziyekelela ndingayityi le treatment yam lomntwana wam ngubani ozothi hayi kuye xa eyodlala kwelidama because elama linzulu and andithi uzobona abanye abantwana bezodlala apha afune udlala naye kanti lilishwa lakhe

So if I can ignore taking my treatment who goes to say no to my child when goes further to play away because that dam is deep and it is not true that he will see other children going there and wants to follow as well and maybe that can his misfortune

1. Um u

Yes

1. So mna like indicingisa into ezininzi yentoba imbhagi yoba mandiyitye phomfu mna ezipilisi zam indicingisa izinto ezininzi because ndithi ndobona ezizizinto ndifumaniseke ukuba ndiva kabuhlungu and ekugqibeleni nam ndisemncinci. Yintoni le nto izobangela into kuba mandingazimiseli like nam ndicinge ngefuture yam and nam ndimbone umntana wam xa ekhula

So it makes me think of many things, the reason of me taking my pills, it makes me me feel sad because if I don’t take my treatment I will die and who will take care of my child. I think about my future and even I can see my child grows up. She will be called names and your mother was killed by virus and other things like that.

1. Um

Yes

1. Akhulele ecaleni kwam ndimchazele ngezinto zobomi

Grow next to me telling her life experiences

1. Um u

Yes

1. Ukuba kwenzeka ntoni na ebomini yabo

So that I can tell her what is happening in life

1. Um

Yes

1. So xa ndingazozimisela mna xa ndingazozimisela mna kuletreatment kuzokwenzeka ntoni ngaye. Ngubani uzokuthi kuye hayi sukuya pha kuwrongo pha

So if I’m not going to be serious, if I’m not going to be serious in this treatment what will happen about her. Who is going to no to her don’t go there, that place is wrong.

1. Um

Yes

1. Kuwrongo phaya kulandawo sukuya pha kuwrongo kulandawo

That place is dangerous don’t go there that place is dangerous

1. Ubenomonde wakhe

To be patient of her

1. Abenomonde wakhe

Someone who will be patient of her

1. Um

Yes

1. So ingaskhe nam ndizimisele ndimbone xa ekhulayo because akekho omnye umntu ozobanomonde ngaphandleni kwam

So I wish I can be serious so that I can witness her growing up because there’s no one who will look after her besides me
