# Supplementary material for: “If I don’t take my treatment, I will die and who will take care of my child?”: An investigation into an inclusive community-led approach to addressing the barriers to HIV treatment adherence by postpartum women living with HIV
Source: PLoS One. 2023 Apr 20;18(4):e0271294. doi: 10.1371/journal.pone.0271294 (PMC10118130; doi:10.1371/journal.pone.0271294)
Supplement: S1 File — (ZIP) [file pone.0271294.s001.zip › 14-18 3rd a.doc]

TR2- 3^RD- 1st^ REC

V1: Good morning today is the 15 16 of May 2018 and I’m with participant number 1418. She’s got photos that she would want to talk about give us some more information on her journey with the treatment. Ungaqala ke ukuthetha sisi. Photo number1

V1: Good morning today is the 15 16 of May 2018 and I’m with participant number 1418. She’s got photos that she would want to talk about give us some more information on her journey with the treatment. You can start talking sisi, photo number1.

V2: foto namba 1 ndifote abantwana bam befunda besenza ihomework, ibizipast eight bahlala bacinga ukuba ngoku kufuneka ndithathe ipilisi. Wathi omnye oyinkwekwe “mama sele utyile ukrolokoxo wakho” ndithi kemna hayi khawuzenaye eze nawo ke sihleke lonto sihleke sihleli apha endlini senditsho ndiyayicinga naleyo imini.

V2: photo number1 I took a picture of mykids doing their homework, its was past eight they thought it was time to take my pill. The boy said “ mama have you taken your pill” and I said no you can bring it and we started laughing

V1: oh yenyinto ekukhuthazayo kuba uyabona nabantwana bakho bayakhuthazeka

V1: oh this is another thing that encourages you because even your children are encouraging you.

V2: noba wenza ihomework uyacinga ukuba lixesha ngoku lokuba mandityipilisi.

V2: even when they are doing their homework they know when its time for me to take mypill

V1: oh ngamanye amazwi wena unayo isupport bakho abanye abantu abakusupportayo?

V1: oh in other words you do have a support system, are there other people that support you?

V2: kakhulu aphendlini bayandisuppoter

V2: they support me well here at home

V1: nabantwana bakho?

V1: even your children

V2: ewe

V2: yes

V1: ungakanani lomntana ebesitsho ngumntana onangaphi?

V1: how old is the child that reminded you?

V2: una 10

V2: he is 10yrs

V1: umntana ona 10 oh yena akakho kwitreatment?

V1: a 10yr old child, is he on treatment?

V2: haha akekho kwitreatment yena

V2: no he is not on treatment

V1: kodwa uyayazi ukuba umama uthatha itreatment

V1: but he knows his mother takes treatment

V2: ewe wade wathi sele umtyile ukroloqo wakho, mama ukrolokoqo kaloku ungamlibali bakhawuleze bomthatha bazenamanzi ndihleli nditye.

V2: yes he even reminds me to take mypill and brings it to me with water and I take it.

V1: nawe ufumana nechance yokuba ubafundise, so xa besenza ihomework nawe ubakho ubancedise?

V1: so you also get a chance to help them with their homework as you sit with them?

V2: ewe ndibakhona ndihlala nabo

V2: yes I sit with them

V1: uyabancedisa

V1: do you assist them?

V2: ndiyabancedisa

V2: I assist them

V1: enkosi ke singagqitha siye kufoto namba 2

V1: thank you can we continue to photo number2

V2: lena indicingisa besuka esikolweni ndihleli apha endlini babauye ke “mama yimeeting ngomso esikolweni ngoku uzakuya njani akuzokuya klinik ngomso kwenzeluba ugqithe esikolweni” ndithi ndothini eskolweni “tyhini mama ayodate yakho akuzoyothatha treatment” ndithi andizoyothatha treatment mna ayodate yam ndisenazo ipilisi nazi niyazibona bahleke lonto ke kubemnandi. Hayi ke yimeeting yeskolo ke ngomso ndithi hayi ke ndizakuya

V2: this one reminds when they were coming from school and I was here at home they said “ mama there is a school meeting tomorrow, are you going to able to go to it are you not going to the clinic maybe you can pass by our school.” I said why should I go to the clinic? “ is it not your date tomorrow for treatment” I said no its not I still have pills here are they and they laughed. I said I will go to the meeting tomorrow

V1: so ababamntwana bakho uye ubaxelele ngedate zakho? Uba yidate yakho yoyothatha itreatment yakho

V1: so do you tell your children about your date? If its your date to collect treatment

V2: ewe bade bandikhumbuze

V2: yes they even remind me

V1: so ngelixesha ubusithi wena ayiyodate yakho kungelixesha wena ubungayityi kakuhle itreatment okanye kungelixesha ubuyitya ngendlela.

V1: so at the time you said its not your date was it at the time when you were not taking your treatment or was it when you were taking it properly.

V2: haha kungelixesha ndiyitya kakuhle, kungelixesha bendisandoyiqala ngoku.

V2: no it’s the time when I was taking it properly, when I was starting to take it

V1: oh ngoku not kudala so ngoku ubungayityi kakuhle bebesithini ngoba kaliku ukhe wangayityi itreatment?

V1: oh now not long ago so when you were not taking it what were they saying?

V2: bebethetha “ mama uziyekentoni wena ipilisi wena uziyekile ipilisi” kuthiwe mama uyanini eklinik ngoba uyabizwa eklinik kuba ngelinye ixesha andibizwa. Uyayakuya nini eklinik ndithi kemna ke andizokuya apho bathi hayi mama yiya eklinik, ufuna ukufa umama hayi ke uzufe kemntakabawo thina asikukhathalele batsho bedlala.

V2: they would ask why I don’t take mypills and they would ask me when am I going to the clinic and I would say im not going there. They used to ask me if I want to die

V1: ngoku wena yintoni eyayikubangela uthi sele unabantu abakukhuthazayo especially abantwana bakho ungafuni ukutya itreatment?

V1: so why didi you default whilst you had people that cared for you especially your children yet you didn’t take your treatment?

V2: hayi mandithi kukungakhathali ngoba ke akukhonto yayindibamba ayikho

V2: no I must say its carelessness because there was nothing

V1: ngoku ayiyoInfluence kwabanye abantu mhlawumbi osendele kubo, bakuinfluencer wrong?

V1: was it not the influence of other people that influence you wrong?

V2: hayi andifuni kuphosisa kodwa ke abakhe bandiseconder bathi like iboyfriend ithi Yoliswa ambotya itreatment ngabantwana abatshoyo nalapha endlini

V2: no I don’t want to lie but I didn’t have other people that encouraged me like myboyfriend telling me to take my treatment, only mychildren were encouraging me to take my treatment

V1: kanene wawusithi neboyfriend iyiyekile itreatment ayiyithathi?

V1: so you said you have a boyfriend that has stop taking treatment?

V2: ewe ibisandoyiyinikwa nguTAC wayitya kakanye yayiyeka yathi ayisoze itye

V2: yes he just got it from TAC and took it for ance and said he will never take it again.

V1: kwakungowuphi unyaka?

V1: which year was that?

V2: kulonyaka

V2: this year

V1: oh ebeqala yena kulonyaka? So wayiyeka , wena wawuqale nini ukuba ungandikhumbuza ngowuphi unyaka, kudala?

V1: oh did he start this year? So stoped it, when did you start taking it if you could remind me which year, is it long?

V2: inoba yitwenty nto yrs ngoku

V2: I think its about twenty something yrs now

V1: uyitya?

V1: taking it?

V2: ewe

V2: yes

V1: twenty nto!

V1: twenty something!

V2: ewe

V2: yes

V1: hayibo kudala so iyasebenza nyani itreatment yakho uyayibona kuba uphilile

V1: that long so your treatment is really working because you are healthy

V2: ngoba ndibenayo ndakuthi ukuziqaphela ngo99 ngoku ndimithe lo unayo ngoku. Lo ukugrade12 ngoku

V2: I was infected around 99 when I was pregnant with the one that is in grade12 now

V1: kodwa wawungekayityi itreatment ngoko

V1: but you didn’t take treatment then

V2: ndandingekayityi itreatment ndizoyitya ena 10yrs, ukuqala kwam ukutya itreatment.

V2: I wasn’t taking treatment I only took it when he was 10yrs when I started taking it.

V1: so akuyiqalanga ngo99

V1: so you didn’t start it in 99

V2: aa

V2: no

V1: so xa usitsho kanjalo kaloku usithi so mhlawumbi uyiqale ngo2010 xa usithi uyiqale ena10?

V1: so if you say so maybe you started it in 2010 if you started it when he was 10?

V2: ewe

V2: yes

V1: umntana ngoka99

V1: the child was born in 99

V2: ewe

V2: yes

V1: so inoba pha 2009 xa usitsho kanjalo usithi ebena10

V1: so maybe around 2009 if you say he was 10

V2: ewe,

V2: yes

V1: and then akukapheli ne10yrs mos uyitya?

V1: and the its not even 10yrs taking your treatment?

V2: ayikapheli

V2: its not yet

V1: usitya itreatment uba uthi uyiqale umntana wako ena10

V1: taking treatment if you say you started when your child was 10

V2: una18yrs ngoku

V2: he is 18yrs now

V1: yhuu yabona ke inoba una8yrs utya itreatment. Uyayibona kodwa ke iyinamsebenzi ungako lonto kodwa ke ndizama ukuthi xa usithi wena uqale umntana ena10 ngoku una18 it means una8yrs uyitya. Hayi ke singagqitha siye kufoto namba 3 andithi ngu2 lo namba 3 ubuzakuthetha ngantoni ke apha?

V1: Yhuu I think its been 8yrs since you have been taking treatment, but that doesn’t matter all im trying to say is if you started when your child was 10 and know he is 18yrs its means its been 8yrs on treatment. We can continue to another photo number3 what were you going to talk about here?

V2: yilabhotile yepilisi

V2: its tha bottle of pills

V1: oh yibhotile le?

V1: oh it’s the bottle?

V2: yilabhotile imhlophe yepilisi, kusebusuku nduvelothathwa kengoku ngabantwana. Ngoku beyibeke apha bayadlala idlala lo uyakroxomisa lo umncinci

V2: its that white bottle of pills, it’s at night mychildren were from fetching me. Know they have put it here and the little one I playing with it.(krolokoqo, the noise that is made by the pills in the bottle)

V1: oh yiyo lento besithi krolokoqo

V1: oh that is why they call it that

V2: athi lomkhulu sukungxolisa ukrolokoqo kamama yitreatment kamama le fihla iyafihlwa lento bayithathe bayibeke phaya amane eyileqa siyayihleka ke thina nosisi sihleli aphekhawutshini siyayihleka lento kakrolokoqo bayayazi itreatment kufuneka ifihliwe.

V2: the older one said don’t play with mama’s treatment, you should hide it they took it away and the little one run after it and we laughed. They know the treatment should be hiden

V1: nomama naye uyayazi?

V1: even your mother knows?

V2: ewe wonke umntu aphendlini uyayazi.

V2: yes everyone here at home knows

V1: ngoku bakukhuthaza ukuba yithathe.

V1: so they encourage you to take it

V2: ewe

V2: yes

V1: masithethe ngofoto namba 4

V1: lets talk about photo number4

V2: ndisegadini apha ndiyaklina kuba ndiqonda ukuba sifuna ukuvusa igadi nosisi. Legadi ipha ngaphambili sincokole ke ba ndifuna ukuvusa legadi ilapha ngaphambili ndingazifumana na izithole. Uyikhuthalele ke igadi uma lowam, abe esithi ngaske afumane into zokugalela amanzi kuba amzi ethanda ukushota. Ivege iyanceda ivege noba unomntana umseze lamanzi ntonintoni siyancokola aphegadini sobabini.

V2: im in the garden here im clearing weeds because we want to plant with sisi. We had had a chat about making our own garden infront of the house and where are going to get seedlings. Mymother like gardening and she said we should get containers for water as the water get scarce here. Veges helps even if you have a child you can cook spinach and let the child drink the water from it its healthy.

V1: ukhe ube nayo ingxaki nje ngobanje uthatha itreatment mhlawumbi ukutya kungabikho okanye ivege ingabikho ungabaweli ukuthatha itreatment andithi kuthwa yithathe sowutyile itreatment ukhe ubenayo longxaki yokuba kungabikho kutya itreatment ke ngoku ungabinamdla wokuyithatha?

V1: do you sometimes encounter problems like not having enough food and you don’t want to take your treatment because they say you must take your treatment after food? Do you ever have that problem?

V2: ewe ikhe ibekhona especially pha ngetwenty nto xa sekukudala kupeyiwe ufumanise ukutya kuyaphela pha nge20 ukuya pha ukuphela kwenyanga kodwa kendingade ndingatyi nditye lonto izanyiweyo nditye ke ipilisi. Okanye ndingazityi ndiDefaulter xa ndiqonda andihluthanga izakundilambisa worse ipilisi ngaske ndiyitye ndihluthi.

V2: yes especially around the twenty something of the month when everything is finished, I sometimes default when I feel im not full because its not easy to take treatment on an empty stomach

V1: so iyayenza into yokuba ungayityi kuba kungekho kutya uzama ukutsho

V1: so you are saying you sometimes don’t take it when you don’t have food?

V2: ngoba ke irumba kabuhlungu

V2: the pill on its own makes you hungry

V1: oh iyalambisa

V1: oh it makes you hungry?

V2: ilambisa kakhulu

V2: too much

V1: so igadi le iyivuselelwisa nayilonto

V1: so that the other reason you are doing garden

V2: kubekho into ekhoyo nokuba kukho lomieliemielie ufumanekileyo kubekho into yokudibanisa.

V2: so that there is something else even if we only have mieliemielie

V1: siya kufoto namba5

V1: lets go to photo number5

V2: lena yeyocinga ezizindlu bangase abantwana bakhule nam ndiphile bafunde bade baphangele okanye nobrother wam afumane umsebenzi sikhe sakhe silungise ezizindlu zisendleni ziphambi kwendlela zimane zijongwa ngabantu ininzi marn into endiyicingayo ngezizindlu. Ba isingathi nemoto xa zigqitha apha zijonga ezizindlu, andiyazi noba zijonga ntoni nditsho xa sihle ti mntakwethu ngske ufumane umsebenzi sisamente abaronta, kuba uyaqaphela noba uhlika etaxini inxibe kakhuhle ujongiwe uzakuhlala kwezizindlu zinje sibe singenamali.

V2: this one is about this houses, I want to see my children grow and find work to help us renovate this house or maybe my brother will get a job and help renovate it. I always say when we talk that I wish he could get a job and assist here at home by renovating this house. Sometimes when we are getting off taxi people are looking at us. I don’t see the need to take pills to live a life like this

V1: ngoku ke itreatment nezizindlu idibana njani? Ngoba kaloku eyonato ndifuna ukuyiqonda kakuhle yinto yetreatment. Uyidibanisa njani into yetreatment nendlu okanye nekhaya eli?

V1: so the treatment and this house how do they mix? Because what I want to know is about the treatment.

V2: iyadibanisa ngoba ndiye ndicinge ngelinye ixesha ukuba ukuba bendinokungabilkho okanye ndiyiyeke lotreatment ndingangabikho. Sometime ndizakuphila ubomi obude ndinganegalelo mhlawumbi ndingafumana umsebenzi noba kuophina ndisebenze ndizokuyenza kento ndiyicingayo. Ithi lonto xa ndingayityi itreatment mhlawumbi ngekhe afezeke amaphupha am ngezizindlu uba kaloku ndizakusweleka ndizishiye man.

V2: it mix because sometimes I think of not taking the treatment and be gone from all this. And then I think no maybe I will leave longer maybe get a job and make mydreams come true.

V1: oh ngamanye amazwi uthi ukuthatha kwakho itreatment kukukhuthaza kuledream, ndisenamaphupha okwenza izinto ezintle ekhayeni, kuba uzakuthi xa usempilweni ukwazi ukuncedisa. Oh hayi ke ndikuva kakuhle ke ngoku, yeyokugqibela le?

V1: oh so in otherwords you say taking you treatment encourages you in your dreams of making you home beautiful because when you are healthy you can be able to help out. Oh know I hear you, is that the last one?

V2: ewe

V2: yes

V1: oh enkosi kakhulu ke sisi ndiyabulela, izakuba yiveki yokugqibela uthatha ifoto ndizakushiya ke otherwise akukabi nazingxaki SIYABULELA

V1: oh thank you so much im greatful, its going to be the last week of taking pictures, don’t you have any problems? We are greatful.
