# Supplementary material for: “If I don’t take my treatment, I will die and who will take care of my child?”: An investigation into an inclusive community-led approach to addressing the barriers to HIV treatment adherence by postpartum women living with HIV
Source: PLoS One. 2023 Apr 20;18(4):e0271294. doi: 10.1371/journal.pone.0271294 (PMC10118130; doi:10.1371/journal.pone.0271294)
Supplement: S1 File — (ZIP) [file pone.0271294.s001.zip › 14-18 3rd b.doc]

REC 2

V1: Good morning today is the 9^th^ of May 2018 and we are with participant number 1418. She hasn’t been able to take any of the photo’s this week but know she’s got some story to tell about what happened during the week. Ungathetha ke mama, aphevekini kwenzeke ntoni nento ebangele ukuba ude ungakwazi nokuthatha nenye ifoto okanye kwanto othe wadibana naye yakwenza ukuba ungakwazi ukuthatha itreatment ngexesha. Okanye into ibikuMotivater ukuba uthahe itreatment ibiyintoni?

V1: Good morning today is the 9^th^ of May2018 and we are with participant number 1418. She hasn’t been able to take any of the photo’s this week but know she’s got some story to tell about what happened during the week. You can speak now mama, what happened during the week that made you not able to take pictures or whatever you came across that made you not take your treatment or something that motivated you to take your treatment.

V2: itreatment yona bendiyithatha kakuhle iveki yonke isizathu ke kukucinga abantwana bam ukuba xa ndinongabikho bangathinina. Enye into eyeyandiphazamisa ukuthatha ifoto kukucinga, ndihlelinje ndiyacinga ukuba iyakuze iphumelele njani lento. Ndiye ndanomsindo kwababantu bomthetho umzekelo abantu bakarhulimente bayaphosisa abakhathali okokoko ndingade ndiyifumane lento yalefood parcel ngalento yaleID.

V2: I was taking my treatment well this week reason being thinking about my children what would they be without me. What discouraged me this week was thinking. I was angry at the government people for example these government people are laying and they don’t care, I haven’t been getting food parcels because of this ID situation.

V1: Ungaphinda usikhumbuze kanene ukuba ubutheni ze ube uzakufumana ifood parcel kwakwenzeke njani?

V1: can you remind us why were you suppose to get food parcels what happened?

V2: yiID,iID yam yayithathwe yiloan shark kudala ngoku ndinabantwana ababini abangagrantiyo.

V2: its my ID, my ID was taken by the loan shark long ago now I have two little children that don’t get grant.

V1: kudala kangakanani iID yakho ingekho?

V1: how long have not had your ID?

V2: kudala kuba omncinci una3yrs ngoku akakabinasatifiketi

V2: its so long because the little one is 3yrs old and she doesn’t even have a birth certificate

V1: so okokoko leID iphaya?

V1: so you ID has been with this loan shark?

V2: okokoko leID iphaya kodwa ndibhatala yavala phaya ayabikho

V2: since then I was paying and the loan shop closed

V1: ubhatala bani?

V1: who are you paying?

V2: bendibhatala lasisi bendiyela etown ayithatha athi ndizakuza nayo iID xa ndijonga imali ishotayo ukuba yimalinina.

V2: I was paying that sisi she would go to town and take her money and say she will check my balance and bring back my ID

V1: ibalance?

V2: ibalance qho xa ndibhatala athi ndizojonga ibalance uba yimalinina ndize neID xa ndibona ibalance ukuba uyayigqiba. Wavala kengoku phayana.

V2: she would say that everytime that she is going to check my balance then bring back my ID until their office closed down.

V1: ok ngoku kwathini ke ngoku ude ube uzakufumana ifood parcel?

V1: ok so how did it come about that you were suppose to get food parcels?

V2: ingxaki bayibona ukuba ibuhlungu imeko yam abasisi kuba ndinabantwana bayi8 ndipeyela abathathu abanye andibapeyeli plus naba bancinci babini and kufuneka nditye itreatment ndityile ndinomtana okugrade11 naye otya itreatment ongagrantiyo. Yaphela igrant yakhe ina18yrs,

V2: they saw my situation was heart breaking and I have 8 children which I only get grant for three, I don’t get grant for others plus I have two little ones and I still need to take treatment on a full stomach. I have a child that is doing grade11 that also takes treatment that doesn’t get grant. His grant stopped when he turned 18yrs.

V1: unangaphi kanti yena ngoku?

V1: how old is he know?

V2: una ugqibe i18yrs ngoNovember

V2: he turned 18 in November

V1: so bayiyekisa igrant nangona esafunda?

V1: so they stopped the grant even though he is still at school?

V2: ewe usafunda

V2: yes he is still at school

V1: ok oh ngoku umsindo wakho nguwo lo ubangela ungakwazi nokuthatha ifoto?

V1: so your anger make you not to want to take photos?

V2: ewe ndazixelela ukuba mna ndiyiyekile yonke lonto leyo bhetere ndihlale nje.

V2: yes I had told myself im not doing anything its better if I just stay like this.

V1: kodwa ke ngoku uzimisele ukuba usezakuqhub aukuthatha ifoto?

V1: but know you are going to take photo’s?

V2: hayi ndizimisele ukuba ndiseza kuqhuba ngoba usisi ondihambelayo uyandicomforter kakuhle uyathetha kamnandi nam ndiye ndibeneworry ndiqonde ukuba izinto azithethayo zoyandakha.

V2: im confident that im going to carry on because the lady that visits me comforts me and she encourages me

V1: ok ngoku ke into yakho yeID njengokuba bekukhe kwakho abantu abakuncedisayo ucinga intoyokuba uzakuyifumana Iid yakho

V1: ok know what going to happen with your ID situation since there were people helping you, do you think you are going to get it?

V2: ndizakuyifumana kuba lasisi waseOfisini phaya kwaKhula bethe ufowunile phambi kwam waphendula ke lasisi wathi zendiye ngolwesibini kuba bendisithi ndingaya na ngomvulo wathi mandiye ngolwesibini kuba ngomvulo kuthanda ukuba busy nakosassa. Mandiye ngolwesibini ndiqale phaya kuye yena uzakuyithatha ibe phaya kuye.

V2: im going to get it because the lady at the Khula offices phoned infront of me and the lady answered and said I must go there on Tuesday because I was saying I will go on Monday and she said it gets too busy on Mondays. She said I must start at her office she is going to take it so I will get from her.

V1: so ngolwesibini kuzabe kungumhla we15 kuzabe kungekhobusy?

V1: so on Tuesday is the 15^th^ is it not going to be busy?

V2: bathe kodwa mandiye ngolwesibini, kuba kuthanda ukungahoyakali bantu ngemivulo.

V2: they said I must go on Tuesday because people don’t get attention on Mondays

V1: so ngamanye amazwi ubangaba ndikuva kakuhle into yokuba kungabikho igrant ungafumani grant ingayichaphazela indlela othatha ngayo itreatment nendlela ophila ngayo ngoba kaloku into ebangela ukuba ube nomsindo ungakwazi nokuthatha ifoto yintoba zingaphumelelanga izicwangciso zakho zokufumana ifood parcel negrant. So into yokuba umntu angabinakutya kakuhle iyachaphazela indlela yokuziphatha nendlela yokuphila.

V1: so if I hear you properly not getting grant can make you not take your treatment because not having grant affect your well being.

V2: iyachaphazela kakhulu, naxa uhleli uqondaba ucinga ukuba ey abantwana bam ngaske ngoku afumane le ayifunayo into. Ngaske ndingamboni bhetere ndingabikho ndingayazi kengoku ukuba ndizakusweleka kengoku ndithini. Kangangokuba nangoku kuleveki iphelileyo bendikhe ndafowunelwa ngulomntana wam usetrongweni esithi kuyabanda phayana ucela iskipa caba phaya kunxitywa iskipa ezinemikhono ezimhlophe nento zothambisa ndazicinga ezonto ndaqonda bhetere kunale imeko yokuba andinayo indlela yoba angazifumana njani ndinoske ndingathathi treatment ndigule ndife. But ndiphinde ndicinge ukuba aba bancinci bazakuphela besithini.

V2: it affects it because even when you think about your children that you wish they could get things that they need, sometimes you wish I couldn’t see their suffering and die but that wont help as well. Even last week I got a call from my son in prison asking for some warm white t shirt I can’t get it for him and I just wish I die rather than to suffer like this I would often just not take my treatment and wait to die. But I thought of the little ones and what would become of them

V1: so nalo usetrongweni usaxhomekeke kuwe? And exhomekeke kuwe nje igrant yiyo le encedisayo kuzozonke ezizinto.

V1: so the one in prison depends on you, and it’s the grant that assist you in all this?

V2: ewe uxhomekeke kum ngoba yonke into kufuneka ayicele kum.

V2: yes he depends on me because he has to ask for everything from me

V1: wayengaphangeli yena ngoku ebanjwa?

V1: was he not working when he was arrested?

V2: wayengaphangeli wayesandukoluka emncinci, ngoka94 besandusuka eTranskie komnye umama endandimgcinise kuye phaya. So ebezokwenza iID apha kum woluka ke besandokoluka

V2: he wasn’t working he had just came back from the bush, he is 94 kid he came back from Transkie from another mama that I gave him to. So he came so that I can make him ID

V1: kanti ubanjwe nini?

V1: when was he arrested?

V2: ubanjwe ngoDec lo

V2: he was arrested in Dec

V1: so umntana ka94 ebengekabinayo iID ngo2017?

V1: so a child fron 94 didn’t have an ID in 2017?

V2: oko ndandimshiye phaya eTranskie

V2: I had left him in Transkie

V1: zange wabanayo iID?

V1: dint he have an ID?

V2: zange abe nayo iID ebeqala ukuba nayo,

V2: he never had an ID its was his first

V1: so esacingela ukuba uzakufuna umsebenzi

V1: so he thought he was going to get a job

V2: ewe kanti kuzakwenzeka lento kengoku eneID sendimolusile, ndiqonda ukuba nam mandimoluse ngalegrant yaba bakhoyo uyase aphangele nam andincedise apha naphaya

V2: yes and this happened, I sent him to the bush with this grant money hoping he would get a job and assist me

V1: umoluse nini?

V1: when did you sent him to the bush?

V2: woluke ngoJune kulonyaka uphelileyo

V2: he was sent in June last year

V1: ngo2017?

V1: in 2017?

V2: kwazokwenzeka lento ngoDecember?

V2: then this happened in December?

V1: oh ubanjwe ngoDec njalo?

V1: oh he ws arrested in Dec

V2: ewe

V2: yes

V1: oh hayike kodwa ke into endiyivuyelayo usafuna ukuba uthathe ifoto and ndinalo ithemba lento yokuba ukusukela namhlanje uzakuqhubeka uthatha ifoto. Nale kengoku into sizakuyithetha nawe emva kwengxoxo sizakuyilungisa njani into yokuba ufumane igrant yabantwana. Ikakhulu kakhulu lomntana ulapha ecreche kuba kaloku kuzakufuneka eregisteritioler for ukungena esikolweni. Hayi ke enkosi kakhulu ke sisi ngexesha lakho, kubuhlungu nje kub ake izinto zakhoazihambi kakuhle. Ngokuncedisana nokusebenzisana likhona ithemba.

V1: oh what makes me happy is that you still want to carry on taking photo’s and im hopeful that as from today you are going to take photo’s. what we are going to talk about after this discussion about how are we going to help you get the childrens grant, especially the one here in crèche she needs to get certificate for school registrations. Ok sisi thank you so much for your time, its painful that things are not happening yet, we are going to assist there is hope
