# Supplementary material for: “If I don’t take my treatment, I will die and who will take care of my child?”: An investigation into an inclusive community-led approach to addressing the barriers to HIV treatment adherence by postpartum women living with HIV
Source: PLoS One. 2023 Apr 20;18(4):e0271294. doi: 10.1371/journal.pone.0271294 (PMC10118130; doi:10.1371/journal.pone.0271294)
Supplement: S2 File — (ZIP) [file pone.0271294.s002.zip › 22-18 4th.doc]

(sometimes I have change ‘sisi’ a ‘woman’ or ‘mis’ so that the word can fit to the sentence, sisi is word we give to the female side as a form of showing respect the level of that person usually with age while the male side use bhuti)

1. Good morning today the date is 26 of March 2018 and we are with the participant number 2218 she will be us telling on the very last week of the programme that she undertook. You can start ke sisi.
2. Good morning today the date is 26 of March 2018 and we are with the participant number 2218 she will be us telling on the very last week of the programme that she undertook. You can start sisi.
3. Okay le photo number 1 ndiyithathe ngoMvulo kuba ndiyendafota njengokuba ndifotile besihleli kwenye indlu, sihleli nosisi waphaya. Usisi waphaya kudala wagula. Akahlali apha elalini. Ukhulela apha. Uhlala eBhayi. Kudala egula ngoku ekubeni sihlali naye ngala mini uyandathanda uhlala nabantwana bangangathi ngathi uyathanda ukuhlaliswa pha kowabo so wasibiza pha nZethu sayotsho phana ubasimncedise uvasa iwashing sacikica. Kweegqi enye intombazana ke

Isuka ingathi isuka eBhayi yagqitha ithi kuye hayi wethu Zukiswa wena ziyakuthanda eziARVS…ndithande landlela amphendule ngayo ngoba uthe kuye hu u, yiza apha ndikuxelele andifuni ube ndikukhwaza njengokuba wena undikhwaza ngeARVS esistratweni, wathi uyabona le ntsholongo yaqala kuthi kungekabikho zipilisi zayo njegokubana kuni zikhona nje nikwazi ukunikwa eyione wathi kuthi zazininzi singazi maneji abantu aabaninzi baabhubha ngokuba bengafuni ukutya le nqwaba yepilisi. Wathi uyandibona mna ndiloluhlobo ndilulo kuba kaloku ndiyazazi so kokoko besihleli naye emana esixelela u u lona mos ucousin wam uyathetha xa enxilile ‘hayi sisi khandiphe…kahndiphe into eluhlaza. Ndiyagula kaloku mna ntoni ntoni.’ Wabe ethanda ke la sisi

Okay this photo number 1, I have taken it on Monday because ihave photographed as I have photographed, we were sitting in another house with the member of that house. This member of that house she has been sick for a long time. She doesn’t stay here in our village. She grew up here. She stays in PE. She is been sick for a long time. When we were sitting with her in that day, she likes to hang around with teenagers; to find out she likes to have company there in her home then she called us Zethu and me, we went there so that we can help her washing, we washed it then. Another girl appeard on the way, seemingly is from PE as well. On its way going past, the girl remarked ‘Zukiswa these ARVS suits you well… I was impressed by theway she responded to her because she said to her, ‘yes, come here let me tell you this I don’t want to be rude going on calling in the street with ARVS,’ she continued, ‘you see this virus started with us in the time when there were no pills for it while in your time there are some for you and you can be given only one’ she said in our time they were many and we couldn’t managed them, many people died because of not wanting to take this whole pack of pills. Then she said you see, me I’m like this because I know myself. We stayed with her all along. This one, any way my cousin like to talk when she drunk, she kept on talking; ‘yes sisi may you please give…may you please, give me something which is green. By the way I’m sick and so on and on.’ This Miss. enjoyed that.

1. Yintoni into eluhlaza?

What is the green thing?

2. Uyathatnda okwakugeza

She likes being silly

1. Um um

Yes

2. ‘khandi phe apha amagqabi ekhapheshu ndiyoziphekela isipinashi ekhaya

Give me please the peels of a cabbage and go cook myself the spinach

1. Ohhh ivege?

Ohhh those are vegetables?

2. Ee ke athi la sisi ndiyayithanda le nto uyithethayo kuba kaloku abanye abantu baya bathi xa bebona mna ndinje ndineemoto ibengathi mna andiguli ntoni ntoni ntoni. So ndamthanda olwahlobo bendihleli ngalo naye.

Yes, and the sister continues to say I like what you are saying, because other people look at me and think I’m not sick when they see my luxery, my cars and other things. So really liked the way I stayed with her.

1. So benimotiveyitha

So she was motivating the two of you

2. Hayi besimotiveyitha gqithi. Kuba nalapha endlini sifike sabalisa… sithi yhu!.. xa umjongile ngumntu owonqenekayo but uthe ngalamini ezosibiza apha. Ndathi yhu lo mntu ungafane ahlale namntu kowabo, but apha ekuthetheni kwakhe saqoda ukuba okay uyakhuthaza kwezinye izinto

Yes she was motivating us a lot, because even when we arrived here at home, we said a story…we said yhoo!...she looks like a difficult person but on that day she came here. I said yhoo! This person who doesn’t go well with the members of her family, but in her ways of speaking we noticed okay she likes to motivate in some other instances.

1. U um

yes

2. Wathi okho niks xa uziva ukuba kuthiwa uyagula yitya itreeatment yakho

She said really there’s nothing when it is said you are sick just take your treatment

1. Okay, siye kweyesibini kangoku

Okay let’s us go to the second one then

2. Eye sibini

The second one

1. Number 2 lo

This one is number 2

2. E e…le photo number 2. Yibegi endiyifoto kuba ndisazi xa ndisiya eklinikhi, ndiphatha yona kwaye ngoku eyona nto iye yandityhafisa abantu bafike befuna ukukwazi ukubana, yintoni caba xa uphethe le begi yintoni lento indisa ngalebhegi inkulu eklinikhi. Ndithi kubo no kaloku ngoku ndinomntana. Apha ndifaka iinapukeni nokutya komntana. Bathi ke bona ke ‘hayi suka uyaxoka uyaxoka behleli pha ngalaamini ngolwesibini ngokuya ndandiye eklinikhi

Yes…this photo number 2. I have photographed this beg because I know when I go to the clinic I carry it, but what worries me is that, all the time people wants to know what makes go to the clinic with a beg as huge as this one. And I say to them, because now I have a baby I put the napkins and the food stuff of the baby in it. And they respond by saying ‘Get away, she is lying, she is lying,...’ on Tuesday by the time I went to the clinic.

1. Um um

yes

2. ‘uyaxoka kaloku siziva ngokroxoma sisiva ngokroxoma noba beyothatha zona.’ Ngoku ndithi kubo hayi yizani. Ndiye ke nyani. Ndiyotsho pha kubo emthunzini. Nditsho ndicela amanzi. Omnye umntana kangoku yhi uzoshukumisa le bhegi kuba ufuna ukuva ukuba yintoni le. So, ithe ngoku seyindityhafisile. Ndaqonda yhuu. Mani, ndiya ngoku…ndaqonda uba ndizoyitshintsha le bhegi. Ndaphinda ndazixelela ukuba ha a. xandiphinda ndisiya eklinikhi ndizophinda ndicele elaa cebo ndandizicelele lona ukufika kwam apha. Because ufika kwam apha. Ndandifika ndinikwe kulanto ngoba ziya ngxola ke pha

‘she is lying because we can their sound by a shaking sound, may she was there to take them (ARVS).’ Now I said to them guys come. I went to them in the shade, semateneously I ask for water. Then the other one from them, look, she comes to shake this beg so that she can know what is inside. So it had happened to me, after I was discouraged. I thought, yhoo! Now I’m going…I said I’m going to change this beg. Then again I told myself that no, when I’m going back again to the clinic I’m going to ask again for that suggestion asked for before the first day I came here. Because the first I came here. They used give me (pills) with that thing (container) they a lot of noise in that that.

1. Phi?

Where?

2. Eklinikhi. Likhona elaqogobhe likhulu kade unikwa kuzo. So ndacela ndathi ha a zingumjojo xanizigalelela pha. So ezam khani zikhuleni eplastiki eplastikini so ndaqonda ukuba okay ukuze bangaziqwalaseli mandenze loo nto leyo.

At clinic, there’s that huge container that they usally hand them to you using it. So I made a request, I said no they attract lot of eyes when they are inside of that container. So mine can you put them inside a plastic instead. So that’s what I thought, if I want them not to take a note of them, I must do that.

1. Ohooo… Bayavuma kangoku eklinikhi?

O! do they comply with in the clinic?

2. Hayi bayavuma ukukhuphelela eplastikini xa ucelayo because baye babuze yintoni unomjojo ewe nzawuts…ndandibaxelele phofu ufike kwam ndathi hayi mna ndinomjojo shame uziphatha oluhlobo.

Yes they comply when you request, to put them inside the plastic, because sometimes they ask ‘why, you don’t want to be the center of attraction’ yes why must I do that…I told them the first time I came here that ‘no I’m very shy to carry them like this (with the container, that makes noise when she walks).

1. But ibhegi yeyakho nje kutheni wena uzozihlupha ngenxa yababantu bafuna ukude bazova ukuba kukho into engxolayo ebhegini yakho?

But the beg belongs to you, why do trouble yourself by the people who are interested of things that are making a noise inside your beg?

2. Yilanto yokubana..i..i..into eyenza iworry yilantokubana kaloku ngoku bakufunda, bayakufunda, ungakhange ubaxelele wena kuba besiva laa krolokoxo. Inomohluko yona labhotile yezapilisi zingxolayo inomohluko yayo

It is that thing of…that…that thing that creates worries. It is that they can predict, study you without even telling them (a status) because the sound that is made by the pills inside the container. They have just their sound when they are inside that bottle.

1. Oooo
2. So bavela ngokufunda ba O! uyakroxomisa lo. Usuka wothatha izinto ezithile. So kubhetele xa size…ndizigalela pha kulaaplasitiki

So they just predict that okay O! she is shaking the container this one.

1. Oooo
2. Iplastiki hayi iye ingavakali niks nie ngoba iyafana ingathi zipilisi neziphina

The plastic, no usually it creates no noise at all. It makes them look like any kind of pills.

1. Ummm, ucinga ukubana nabanye abantu babanayo ingxaki yokubana icontainer lena ibenzela umjojo?

Yes, do you think even others (patients of HIV) are experiencing the same problem with the container, that it makes them the center of attraction?

2. Ewe

Yes

1. Okanye ku…mhlawumbi nguwe qha akukuba ngababantu balapha ekuhlaleni aba bathanda izinto mhlawumbi nabanye abantu baye babenayo intetho enjalo

Or it’s like…maybe it’s only you who think in this way. It not like are these people who likes other people’s businesses or maybe other people (patient of HIV) have the same remark about the container?

2. Ha a…abantu abanintsi baye bayikhalazele laa nto yala*tromile* but mna ndandi..ngayikhathalelanga kuba ngalaxesha bendise Bhayi. EBhayi mosi akukho mntu undaziyo

Yes…most of the people they do complain about that container as well but me I didn’t…I didn’t care about it because by that time I was in PE. Anyway in PE there is nobody who knows me.

1. Oooo
2. Bendifika qha ndithathe ndihambe, so bengena lanto ukubana zakroxoma. Andaziwa mos kwesisistrato ukubana…ndihla…ndizophangela apha akukho mntu undaziyo so kangoku, apha bona…

I used to arrive just take my medication and go, so it had that feeling that the container will make a noise when I walk. Because there’s nobody who knows in this street if I’m…(HIV positive)…I’m staying…I’m here for working commitments, there’s nobody who knows me , but these one here…

1. Kuba bekwazi

Because of they know (status)

2. Bendazi ukhe ubone, bavela ngokufunda bazigqibele ngawe ukuba nabo ukubana sihleli nabo sometimes uyava apha ezincokweni zabo ukubana ‘iyadika le nto yenziwa ngusister Midas, afune ukubana i…i…ezipilisi makakunike ngala*tromile* because kaloku iyangxola le nto. Ubonwa…ubonwa nje xa usiya yothatha ezipili – le treatment uphuma egumbhini aba bantu badwele apha ezitulweni bavela ngoyiva laanto kanti sasibaxelel iplani yokuba mabasikhuphelele noba umntu uze neplasitiki yakhe, umntu agalelelwe phaya’ nabo iyabahlupha nangona ke beya umntu avela ngonikwa la*tromile* avele ngothatha afake ebhekhini kuba kebona bebena nalanto yokubana because abanye bona ngaba sisi sebebadala…

Because they know me you know, they just conclude about your status. Sometimes even them when are just chilling you hear these remarks ‘the thing that she does sister Midas, is annoying of her insisting of giving you the pills with that container. Because that thing makes such a noise. People just notices you when you are going for your pills – this treatment, when you are coming out of that room, this people who are lining up on those chairs; they just pick the shaking noise of that thing. The plan should be of them transferring these pills, even if maybe someone comes with his or her plastic, so that they can put the treatment of anyone on his or her arrival’ even them it troubles them although someone was just given that container (tromile) and just take it inside the beg, because of that I don’t care attitude anyway these are those older ones…

1. Okay
2. umntu uvela ngothatha i*tromile* yakhe athetha afake ebhegini…athi ekugqibeleni andizifihli mna… kudala…ndaziwa mna ukuba ndiya gula.

Someone just take his or her container (tromile) and put it inside the beg….and say at the end I don’t hide my status…it’s been years so sick I’m being known about that.

1. U m

yes

2. So ke thina sisebancinci fori ukubana siyaqhayisa ngale*tromile*

So us we are still young for that of doing an open about this container (tromile)

1. Oooo. Okay ndiyakuva. Siye kunamba 3…mna (sound of papers) kuphi kanene…okay.

Oooo. Okay I hear you. Let’s going to number 3…

2. Lo nantuka namba 3 yena ndifote le suitcase

This number 3 here I have photographed this suitcase

1. Yhaa

yes

2. Kuba

Because

1. Okay
2. Ndifote le suitcase kuba ngalaxesha ndandiseBhayi

I have photographed this suitcase because by the time I was in PE

1. Um um

Yes

2. Ndafika eBhayi ndingena suitcase. Ke le ndlu ndihla kuyo kukho oobuilt in bewadrobe

I came in PE without having a suitcase. Then in this house I’m staying in it there are built in wardrobes.

1. O
2. Ngelixesha bendiyokuqala ukuthatha le treatment. Zange ndifike mos ndivele ngobaxelela ngalasuku lokuqala

By the first time I was there to take the treatment. I didn’t came and tell them by the first day

1. Okay
2. Ndakhe ndalala olasuku lokuqala. Ndabe ndiqonda ndiyazicingela ndingaxelela bani ndixelele bani so ndaqonda ukuba eyi..ndiza…ngoku kangoku sezikum nazi ezi nantsika zilapha kum ebhegini. Ndaqonda ndizozifaka phi kuba…andinasuitcase ilapha..andinayo nebhegi…isuitcase bendize ngayo apha ibiseyikrazukile ndayithatha ndayoyilahla. Ndapeya ndathi upeya kwam ndahamba ndayozithengela isuitcase. Nadmane kangoku ezi pilisi ndifaka pha..ndizifaka pha. So ngenye imini ndithi ndiyaklina ke (a sound of a babe) (background voice uphi yena) ukhona umntu…ngubani lowo…ikhona icousin enye yapha

I slept the first day. Thinking ‘I wonder who must I tell, who must I tell,’ so I continued to think ‘I wonder… I will tell who… now… then here are these things with me inside the beg .’ I thought whrer I’m going to place because… I don’t have a suitcase here… even a beg I don’t have… the suitcase I came here with I threw it away becaused it already apart. Then I got paid (grant money) when I was paid I bought myself a suitcase. And then I began to get used in placing these pills in it. I put them in it… then I was cleaning… there is another cousin who belongs there.

1. Um um

Yes

2. Enye icousin yapha kuba ndidndi…akho…mos uzovela ngokuthi emntwini ‘hayi suyiphatha into ethile yam

Their cousin… any way it is not easy to say to someone ‘don’t touch that thing, it belongs to me’

1. O…e e

O…yes

2. Ndibone ngaye ekhuphela isuitcase yam…’hayi kuthiwa makuklinwe yonke impahla apha nam ndizo songa…lo built in wale wardrobe nale suitcase yakho xa esithi ubona eeeeliqogobhe…wayeyinesi lasisi ngaphambili

She just scoops my suitcase down….’they are saying here we must clean everything, even me I’m going to pack…this built in wardrobe and your suitcase’ wwhen she sees this container…thist woman was a nurse.

1. O
2. Abone ezipilisi ‘Mazandi, yiza apha mntana’ m zintoni ezi’ ndithi ke mna ‘hayi mani sisi Dezi zipilisi ezi’ ‘zezikabani’ ndithi ke mna ‘zezam’ andibuze zezantoni

And she sees these pills ‘Mazandi, come here my child, what are these?’ and then I say ‘no sis’ Dezi these are pills’ ‘of who’ then I say ‘of me’ then she asks ‘of what’

1. Ungekaba xeleli wena?

You haven’t told them?

2. Hayi ndandingakaba xeleli

Yes by that time I haven’t told them

1. O
2. But nda…yayisandakba yiveki ndisandoya eklinikhi

But it was some few days from the clinic

1. O
2. Qha ndisaqonda ndizoyiqala ngaphi

I was still thinking of how I’m going to tell them

1. Mmmm

yes

2. Ukuyithetha kwam kubo bendiyithethat ukubana ndiyajoka

I was telling them in a joking manner

1. Okay
2. Xa sigqibo kutya ndiphakame ndithi ‘o hayi ke mna ndotya itreatment yam’ ntoni ntoni ukhe ubone

After a meal,stand up and say ‘okay guys let megoing and take my treatmsnt’ so and on

1. Um um

Yes

2. Bona abaunderstandi ukubana ndibhekisele entweni

So they don’t understand my point

1. Um um

Yes

2. So lo sisi akhuphe ke ‘hayi, yintoni le mntwana, cacubana ziARVS ezi

So she pulls out ‘what is this child, it looks like these are ARVS…

1. E e

yes

2. Ndithi ke mna ke ‘hayi andizazi’. Hayi kutheni unikwa ezi pilisi? Hayi kaloku kuba bendiyele ukuqhina bendinako eklininki mna ndibone sendinikwa ezipilisi… Hayi mntwanam unikwe ipilisi eziwrongo. Ndabangathi kuye ndingumntu ozobuyela ekliniki ndiyoxela ntoni ntoni ntoni. Kulaphoo kangoku ndaye ndazithatha ndazifaka ngaphantsi apha esuitkheyisini so kangoku njengokuba ndifote le suitcase indikhumbuza ngelaaxesha because nalapha endlini ngelaxesha ndifika ndithi kubo ‘eyi nako ndifumana idyavi eBhayi ussis Dezi egubhulula le suitcase.

Then I say ‘no I don’t know what are these?’ No, I was there for constipation in the clinic, they just gave me these pills… ‘no my child they have given you the wrong ones.’ And then I acted as if I was going to go back again to the clinic and so on. It is then that I decided to put them right beneath my clothes here inside my suitcase. So that’s why I have photographed this suitcase it reminds me of that time even when I arrived here at home I told them ‘Ay there I get an embarrassment in PE, sisi Dezi scoops my suitcase.’

1. Um um

Yes

2. Ebhaqa ezilantuka

Find these

1. Um um, ngumntu enimaziyo enizala naye lo…usisi

Yes, isn’t someone you know…this woman

2. Lo lo sisi Detsi…ngucousin wapha

This sisi Dezi…. It is their cousin

1. O kodwa akangomntu nimaziyo

But it is not someone who do you know

2. Andimazi mna…bayamazi bona aba phana..kaloku mna njengomntu beyokusenzela phaya

I don’t know her…they know her… I was only there for working purposes

1. O ubuphangela wena pha ubungazalani nabo

O, you were just for working purpose you were not related with them

2. Ewe so bona bebe mana befikika iicousin zabo…qha xa kuklinwa kufumaniseke ukubana sonke apha endlini sibabhizi asikuba kuthiwa uqeshiwe wena uzokwenza yonke into ha a. beklina sikline sonke kondlulwe kuthweni newashing ivaswa ngumntu wonke bana nangenye igame andiyazi kwakusebusuku ndahamba ndayoyithatha u8 ubethile ngelixesha sendibaxelele. Ndiyithathe kangoku. Andiyazi ukubana ndiphaza nyiswe yintoni. Ndise ndiyifaka apha kum empokothweni

Yes, so their cousins usally come now and then… but when it is time for cleaning, all the people do the cleaning it is not like you’re hired to do the cleaning job as yours only. No. all of us we clean, we do the bedding and other things, we do the washing, the clothes are washed by everyone even by the other time if I can rmember I’m not sure it was at night I went to take it (treatment) it was already past eight by this time I was already told them.

1. Um

Yes

2. Ndihlale. Ngelixesha kuvaswa le washing ndibizwe ngulosisi ‘Mazandi, kaloku mntana wam ezizinto zakho suzibeka careless, kuba kukho umntana apha one6 years osafundayo because uzovela ngothatha

I stayed. By this time when this washing was being made here this woman calls me ‘Mazandi, look my child don’t leave these things of yours careless, there’s a 6 years child here who is still curious of experimenting, he/she just take them.

1. Um um

Yes

2. ‘Uzovela ngothatha’ ndithi ke mna okay sisi zange abe ethetha ukuba yintoni zintoni ezi zintoni ntoni ntoni njengokuba lo ndithetha ngaye ukuba. Bendisithi ukuba ndiphumile. Kuba ndathi mna ndingekabaxeleli. Ndithi bandiphumile ndive uba…uyababona mosi abantu apha efacesini

‘He/she can just take them’ then I say okay sisi, she didn’t said much saying this and that like this one I’m talking about, because it was like this if I went out. Because I have not told them yet, but if I went out, pick up some remarks….you can tell by the look from their faces that there’s something wrong.

1. Um

Yes

2. Ndiqonda ukuba kwenzeka ntoni apha. Kutheni ingathi namhlanje akuxolwanga kuze kangoku ndiqonde ukuba hayi suka mandibaxelele but zabathethe

I thought what is happenng here? Why it seems like people are not happy now, then I was defeated I decided to tell them.

1. O ucimba mhlawumbi kuba wayeyinesi naye. Ayinoba mhlawumbi pha eklinikhui kukho umntu owafumanayo ukuba ubuze apha uzothatha itreatment kuba wayeyinesi naye

O you think because maybe she was a nurse. It is not that she a have a contact maybe inside the clinic, that she asks from if you were there to take a treatment.

2. Ha a because intobana wayeyinesi andiyazi ndizothi nini

No because she was a nurse, I don’t know what I’m going to say now

1. Um

Yes

2. Kwakukudala ngoko

It was long ago

1. So akazani nabantu basekliniki

So she is not in cotact with the clinic people

2. Akazani nezinesi zikhoyo ngoku

She doesn’t know these new nurses

1. O
2. Ha a abazani

No she does’t know them

1. Hayi into ebangela ndibuze yinto yokuba ndicimba ngalento uthi waskhe wathi xa ezosorta iimpahla askhe afune usorta nezi zisesuitcasini

The thing that makes me ask is this, when she came to sort the clothes and sorted yours too inside your suitcase

2. E e asele ebuza ubana hayi ke…

Yes and she then she asked…

1. Ngokungathi kukho into ebeyirhanela

As if there’s was something she didn’t trust

2. Into ayaziyo…ngoba ke waye wa… ndambona ukhona losisi la aba…ezitshomi zabo basela bonke

Something she knew… because she began to… I noticed that there’s this woman –these …these friends of her that she goes out with to have drinks

1. Um

Yes

2. Ngoku ngenye imini ndogqitha pha ndibeka evenkileni… athi kengoku lo sisi ‘heee! Kuthiwa ukhulelwe uzoza nomntwana…uzoza nomntwana otya ine*Varapi.’* Awu! Lo sisi mosi akandazi

Then some other day going past there on my way to the spaza shop…this woman said ‘heee! I have heared that you are pregnant. You are going to bring the babe…the babe who takes ‘Varapi’.’ Ah! This woman doesn’t know me

1. Um

yes

2. Ke mna ke… Njani yintoni lo nto? Nam ndizezenze lost

And this is me… How, what is that? I began to make myself confused

1. Ewe

Yes

2. ‘Hayi kaloku la mntu uhlala naye pha koweni unolwimi akhonto angasixeleliyo yona ngawe’ qha ndabe mosi ndiqonda uba andinaxesha lenu mna ngoba anindazi nondazi ndinibona nje xa ndi…mhlawumbi ndizobiza yena apha. Kuthiwa uyabizwa endlini

‘anyway your family likes to gossip nothing she misses not to tell us about you’ you know what then reminded myself I don’t care about these people they don’t even know me I see them when… maybe when I’m asked to call her here. With a note they are calling you at home.

1. Um

Yes

2. So ndabe ndiqonda okay inoba uyathetha ngam lo

Then I realized okay this one talks about me behind my back

1. Um

Yes

2. ‘Ba lona uloluhlobo noluhlobo noluhlobo’

‘That this one is like this and like this’

1. Ewe, okay. Kutheni ingathi icimile uxolo kancinci. Ingathi kucime kwale…okay…icinyiswe yintoni khange ide iphele ibhetri apha?

Yes, okay. Why it’s like it’s dead (camera) sorry. It’s like even this one is off…okay…what made it off. The battery was not flat?

2. Ha a

No

1. Okay…nantso ke…uxolo masiqalise lena yesine

Okay… that’t it… sorry let’s begin again this is the fourth one

2. Lena yesine

The fourth one

1. Number 4

Number 4

2. Le foto namba 4 yona. Ndifote le top kuba le ‘top’ (impahla) …ikhona ngenye imi…ngexesha ndiseRhini. Ndihlala nefriend yam

This photo number 4. I have photographed this ‘top’ (clothing)…. There was another day… while I was still in Grahamstown. Staying with my friend

1. Um

yes

2. Siyaphangela…ndaavuka mna ndanxiba le top. Ndinxibe le top nefriend yam sehle. Qha ifriend yam ithi ngale mini ayibaweli uvuka iTS…iTS mosi bekuphangelwa apha ekuseni o2

We were jobbing… I woke up and then I put on this top. I wore this top with my friend we went off… but my said in this day. She doesn’t want go to wortk at Chillis…at Chillis we work at dawn around 2 o’clock

1. Oh! Ubuphangela phaya

O! you were working there

2. E e. ndithi kuye. Hee mani! Ndithi mna ngoku sendigqibile ukunxiba. Ndithi hamba mtshana thatha nantsi itopu. Nxiba letopu kuba usithi uyonqena ubugubhulula ngoba besilayita lento icandle apha

Yes. Then I say to her. You know! Then I said after I had finish to put on my clothes. My friend here is a top take it. Wear this top because you said are lazy to search for yours. We were using candles here.

1. Um

yes

2. Ndimnike…ahambe ke le tshomi yam ‘hayi ke Mazandi ndahamba’ ndithi hayi mani if ndibuziwe ngabantu begroup yam uzusele usithi uphathwe yintloko. Ndiqonde mxim andinamdla mani ndiyonqena. Yahamba ifriend yam yabuya noba yabuya pha ngee to four, ibuye ivele ngotyhala ucango apha endlini ‘Mazandi, Mazandi ndireyityiwe’ ndithi ke mna ke phi

I give her… this friend of mine goes ‘Mazandi I’m leaving’ then I say to her if they asked me at work my where about say I have terrible headach. A nnoyed very moody I felt very lazy. My friend left and came back maybe around to four. When she came back home she just pushed the door open ‘Mazandi , Mazandi I’m raped’ then asked where

1. E e

Yes

2. ‘Apha endleleni apha etheni’ ndithi ke mna ke hayi suka uyaxoka. Nditsho ndizgquma

There on the way, there on the tar road’ then I said no get away you are lying, pulling blanet over my head

1. Um um

Yes

2. Ndithi hasuka uyaxoka mani. Akabonakalisi nto ukuba uyakhala

I said no you are lying now. She was not showing any signs that she was crying

1. E e

Yes

2. Nyani akhwele ebhedini. Ndibuze iphi ifowuni. Athi ifowuni ixuthiwe

She climbed on the bed. I asked where is the phone? he answeres back it was snached

1. Hayi bo

No

2. Ixuthwe ngababantu. Ndibuze ngabantu abangaphi. Athi bathathu. Ndithi ngoku ucinga uthini ngoba seyingo4 ngoku ekusini. Athi hayi ndizolala…ndizo…ndizokubona uvuka kwam. Sivuke nyani. Athi uya ePolistation

I was snached by these people. Then I asked how many were they? Then she answers back they are three. Then I asked her so what are you going to do about this, because the time was around 4 at dawn. She says I’m going to sleep… I’ll see this when I wake up tomorrow. Truly we woke up. Then she said she is going to the police station.

1. So kumaa ebuya emsebenzini ngo4

So it was by the time she was coming back from work around 4

2. Uyaya…amaxesha

She is going…these are the times

1. O
2. Ngo2 wasekuseni ukubethela upha

When 2 o’clock strikes you should be there already

1. Iyhoo!
2. U2 wasekuseni

2 o’clock of the morning

1. Um umm

Yes

2. Ndithi kuye okay athi hayi uzocela enye ifriend yethu ekwakuthiwa ngumaGaba naye ngumntana ebemdala nje apha kuthi caba naye kudala wagula but bengathethi akazixeli but ezincokweni zethu mhlawumbi sithe ‘heee uyehla ubani eweyithini’. Athi hayi kaloku mna ntoni ntoni ntoni abakwazi ukopa kum jongani mna anisoze nitsho ukba…qha thina sicimba intokba uyajowuka. Because siyamazi uhleli nje ungulamntu because usisi slender kakade…so kangoku lona abuye…ahambe…ndishiye ke mna ke apha endlini…yifriend yam le yaseTranskei…ndihlala nayo ndayithatha ndahlala nayo

Then I said to her okay then she says she is going to ask another friend of ours maGaba, she is just a friend of ours who is bit older than us, even her it’s been years so sick but she was not disclosing anything about her status but in our conversations maybe we said ‘heee so and so is losing weight’. Then she says this and that I don’t know why they are setting me as their example copy what I do, guys look at me. You won’t say that I’m…but us we think that she is joking because we know her she is that person who likes to do that and thing is she is a slender..then this one comes back…goes away again…then I stay behind here at home…this is my friend this one of Trankei…I’m sharing a house with her I took her and stayed with her

1. Okay
2. Ndayithatha ndahlala nayo ngokwam kuba ingenandawo yokuhlala eRhini

I took her and stayed with her because she no place to stay here in Grahamstown

1. Uum

Yes

2. Ayizange indixele ukuba kunjena kuyo

She didn’t told me about her status is like this (positive)

1. Um

Yes

2. Sahlala sahlala iminyaka kwazofika iboyfriend yakhe pha ngoo6 semkile andayazi iboyfriend yakhe ive ngabani because lento yenzeke ekuseni

We stayed years and years… her boyfriend arrived around 6 o’clock she was already not around I don’t who told her boyfriend because this had occurred in the morning

1. Um um

Yes

2. Ibuze kum… isele le boyfriend yakhe…ubana uphi uZolelwa. Ndithi kemna uZolelwa uthe uya ePlisistation… ‘ukuxelele na uZolelwa ukuba uretyiwe and eretyiwe nje…uyakuxelela ukubana utya itreatment yeHIV’ ndithi kemnake yhuu! Hayi andiyazi. Hayi yiminyaka ndihlala naye

He asks from me…this boyfriend of hers is drank… if where is Zolelwa. And I say Zolelwa said is she is going to the police staion… ‘did Zolelwa told you that she is raped, and she takes the treatment of HIV? Then I say ‘yhuu! I don’t know. And it’s been years staying with her

1. Um

Yes

2. But akazange ayiphathe le nto injena kum. Ndothuke kemnake ndiqonde ukuba heyi, mandilindele abuye. Abuye. Ndibuze ubambile. Athi ewe. Apha endlini kangoku njengokuba ndiklina. Ndiphakamise umatrasi. Ekuwuphakamiseni kwam umatrasi kwelicala alala ngakulo ndibone eliqwewqwe. Ndandingazazi kemnake ukuba zinjani ni na

But she never touched that this thing is like this to me. And I was shocked. I thought I must just wait for her to come back. Ultimately she comes back. I asked her did you manage to report this to the police station. She says yes. Then here at home while I’m doing a spring cleaning, I lift the mattress, after I have lifted the mattress on the side where she sleeps I s ee a container. I wasn’t aware of how they look

1. O ubungakazisebenzisi wena

O you were not using them

2. Hu u..bendingekazisebenzisi…ndioqala eBhayi mna ukuzisebenzisa

No I was not using them then…I started using them when I came in PE

1. Um u

Yes

2. Ndibone eliqweqwe ndilithathe kangoku ngokubana ndiyaklina. Ndolilahla etoile. Ukubana zipili ezitheni ezi. Njengokubana iliqweqwe nje zither saa apha ebhedini iqweqwe livulekile ndizithathe mna ndozifaka etoilet abuye. ‘Hee Mazandi’ ndiyambona ukuba uyakhangela. Ndingangxami ngokuthi ‘iboyfriend yakho ibilapha. Yandixelela uku…’ akhangele abuze ‘hayi mani Mazndi khange ubone plasitiki apha iipili?’ ndithi ke mnake hayi iipilisi bezilapha ndizifake etoilet ‘hayi Mazandi ngongazilahlanga, ndithi ke mnake zezikabani ‘zezam ezapilisi Mazandi’ atsho ke esithi ‘hayi mtshana ndandngaifuna ukuxelela kuba sihla..hlale sixabane apha endlini silwe sithukane ndandisoyika mhlawumbi ungandithuka ngaloo nto

I see this container and I took it because I was cleaning, to throw it inside the toilet. If these are pills of what. They were scattered everywhere on the base of the bed, the container was open. I took them and threw them inside the toilet. She came back. ‘HeeMazandi’ I can see her she is searching for something. I was not in a haste to say her boyfriend was here and told me that…she continued searching and then she asked ‘Mazandi din’t you see any plastic here…the pills?’ Then I answered by saying the pills which were here I threw them inside the toilet then she said ‘no Mazandi you should have not done that’ then I asked they belong o who she said ‘these pills are mine Mazandi’ at very same time saying ‘you know my friend I didn’t wanted to tell you about my status because now and then we quarrel here at home I was scared that if told you about it you were going to swear at me about my status

1. O um

O yes

2. Ndithi kemna yintoni dani zezantoni ‘hayi mtshana kuloluhlobo noluhlobo’ ndthi ke mna oh ndithi kemnake ukhe ubone lanto ukuba andifuni ukumtyafisa ndithi hayi mtshana kaloku kuba besihleli nje sihamba kunye. Nam kufuneka ndiye ndoziqonda nam

Then I say these pills are of what ‘my friend I’m like this (hiv positive)’ then I say…did you ever get that feeling that you don’t want pull her confidence down then I said my friend because we were always together I must go and do an HIV test as well…

Um um

yes

1. Athi hayi akho ngxaki ndizokukhapha tshomi yam. Sahamba kenyani sobabini qha xa ndifika ngalaxesha kuthiwa ha a andikabi lolohlobo mna
2. then she said there’s no problem my friend I’m going to accompany you. We went together but by that time I was tested negative
1. Mmm

Yes

2. Ndabuya kangaoku ndamxelela wathi ‘hayi akho niks nie’ but yena eyano nto yayimstressa ‘eyi ndineworry ubana ndiya funa umxelela umama wam but ndizomxelela njani kuba umama wam unecancer yesibeleko

Then I came back and I told her. She said ‘there’s no problem’ but her the only thing was stressing her ‘ay I’m worried I want to tell my mother but I don’t know how because she has a cancer of ….

1. O
2. And enecancer yesibeleko nje njengomama’m ulolihlobo

And she has both cancer of … my mother…she has this like me

1. Um

yes

2. Ngoku ebudaleni bakhe but kengoku nda…mosi kangoku mna ndingulamntu qondikuba yhu ndiyam yeka. Ndayoxelela lo ndithi ubana wayesilender umo wakhe. Ndixelele umaGaba ke njengokuba esonwabile uba ‘hee maGaba apha…akekho ke lo ndihlala naye. Ndiyathetha ke eyi…uSi..unento enje into enje into…lo mntana sumbona ehleli nje uyakhla uyakha uthi ABCD’ wathi umaGaba okay myele kum wayethanda ke yena. Wayethanda ke yena uyasithehtisa. Wambiza nyani uSisindiswa pha kuye endlini bathetha bathetha but wabuya kengoku ndambona ukuba ukhululekile

Now at her old age but then I did…any way I just leave alone a person who…then I went to tell this one I said she slender by nature. I told maGaba while she was still in the happy mood. ‘Heee maGaba’… This one I stay with is not around by the way… ‘Eyi… uSi…she has something which is like this…don’t see this child always crying and saying that ABCD’ maGaba said leave her to me, she was the person who was usefull in putting us on the right way. Truly she called Sindiswa to visit her in her home. She spoke with her and she came became as a friendly person.

1. Um

Yes

2. Wayeka ukundifihlela kangoku. Naxa engazokuya. Mhlawumbi uyaphangele because mna ndaphela ndiyeka

Then she stopped to hide things for me. Even when she wasn’t going to attend. Maybe she is going to work because I’m no longer working

1. Um

Yes

2. kuLow Chillis. Andisoze mna ndibe nndizireyipisa because ndandingena xa kungenwa apha ngo12 emini ukuba yilashift yokungena pha ngo2 ndingayi mna iveki yonke. So kwafumaniseka ngelixesha kudendwa kwadendwa thina bantu bebengaphangeli kakuhle

in that Chillies. I’m not going to make myself a victm of rape because I used to go to work in a day time shift around 2, if is that shit of 2 o’clock at dawn I used not to work the whole week. So when it was the time of retrenchement we were the first ones to be retrenched because we were not consistant on work.

1. um um

yes

2. so ndaqonda ha a ndiyalento…kangoku

so I thought no I’m doing this…then

1. so ngoku ikukhumbu ntoni kanye kanye le ntopu le

so this top reminds you of what

2. letopu kaloku indikhumbuza into bana. Ela xesha ndandi yinika ifriend yam ukum nika kwam because bendi ngekho ngaka mosi kuqala

this top reminds me of this…it reminds me by the time I was handing it over to my friend…I was not in this weight at first

1. o
2. naye, yena mna bendi but owam umzimba ubusemkhulu kunowakhe ngoku ndithi xa ndijonga letopu mna ngoku sendiyinxiba indilingana le topu ndandiyithenge ngo2014 uqala upeya kwam eChillisini

even her, by my body was bit bigger than hers but now when I look at top trying it on and fits me well. I bought this top at 2014 with my first payment of Chillis

1. um um

Yes

2. ndithi ukubana ndiyinxibile ndiqonde ukubana hayi iryt…inkulu

so when I try it, to think that it’s fine… it’s big

1. um

yes

2. but iryt… ngokuthi ndithi xa ndiyi…ngokubendimana ngokuthi le mpahla yam…ndithi heyi kukho lejini nale topu…ndazithenga ngemali yaseKh…zazinkulu.. zinkulu but ndisiya eklinikhi kungade kufunyanwe nto

but it’s fine…so when I tried it sometimes…I used to said, these clothes…there was jean and this top… I bought them with the money of…they were big but I was attending clinic. They finding nothing

1. o
2. kulapho kuze kuqale bathi. Hayi mani yileTB

it is then when they began to say that it is TB

1. o
2. uzotya itreatment yakho ethi for 6 months…so nangoku njengokuba ndihleli nje. Ndithi heee because le topu ndisanda kuyithatha kuye kulonyaka uphelileyo ngoku ndipreg. Bendikhe ndaya mos kuye ngoku ndine7 months

you are going to take your treatment for 6 months…even now… I was shocked because I have just taken this top from her last year when I was pregmnant. I visited her when I had a 7 months.

1. um

yes

2. ndakhwela ndayotsho eRhini

I took a ride and to Grahamstown

1. um

yes

2. ngoku singqengqile mos ebhedini siyancokola ‘E mtshana iphi la topu yakho, wawundinike yona..ntoni ntoni ntoni

while we were lying on bed chatting ‘E my friend where is that top of yours, the one you gave me and so on and on’

1. um

yes

2. ‘usakhumbula ukubana kwenzeka ntoni ngala mini yala topu’ ndithi kemnake hayi ke mtshana isekhona lootopu ndiyayazi

‘do you still remember the incident of that day of this’ then I said yes my friend the top is still around

1. Um

yes

2. ndiyayazi isitori sayo

I know the story

1. um

yes

2. hayike mtshana ndomelela

anyone my friend I got strong

1. o
2. and nalomntu ndihlala naye apha endlini. Ndamxelela ukuba ndiloluhlobo ndilulo but wathi akanagxaki kuba naye akazazi uthi but njengokuba sihlala sobabini ndimthathile ndamsa apha ekliniki e7 uthi watesta watsho ke endibonisa iipilisi zabo. Wathi njengokuba eleli kwelicala nazi ezakhe nazi ezam ngapha.’ Ndathi hayi mtshana akukho niks as long qha usitya itreatment yakho

and that person I shared a house with I told him that I’m like this but he said he doesn’t have a problem because either he wasn’t aware anyway about his status but because we are staying together I took him to the clinic at 7, she told me that he tested and began to show me his results. Then she said his treatmement is at the side of the bed where he sleeps and hers on her side where she sleeps. And I said there’s nothing as you take you take your treatment .

1. um um..okay..oryt. nantsi kangoku elandelayo…unamba 5

yes…okay…alright. Here is the next one number 5

2. unamba 5…

number 5

1. e e …namba 5

yes number 5

2. o…okay yile…lo…le tafile yona…le natsika namba 5

o…okay this…the one…this table…this thing number 5

1. um um

yes

2. ndiye ndacinga

ithought

1. kulapha endlini apha

it is here at home

2. yhee kulapha endlini

yes it is here at home

1. o
2. but mna bendiqonda kuba biba…yinto bendiyicinge ndiseklinikhi

but I knew I was influenced by the thing which was in the clinic

1. um u

yes

2. but ndabe ndiqonda ngalaamini mosi ndoyikile uphatha le camera. Ndaqonda uba o abantu bezobe ‘hayi sukwenza into ethile ntoni ntoni ntoni ujajwe ubengathi ufuna…andiyazi ufuna uthengisa ngabo abantu balapha mosi ucinga…so ndaqonda uba okay ingqondo yam izandikhumbuza mandithathe kwalapha endlini le lantuka

but I remembered on this day I was scared to carry with the camera. I knew prior that people are going to react in this way…’no don’t do this thing and that thing, and judged as if you want…as if you want to sell them in anyway, people of this place they thing in that way…so I thought anyway my mind will remind me again let just photographed this here at home.

1. o okay
2. le na ke ndiyithathe ndisekliniki (maybe photo number 6). Usister ecenga omnye umntana

I have photographed this while I was at the clinic (maybe photo number 6) the sister trying to convince the other girl

1. um um

yes

2. inoba walibala. Ngoku ubusandokuza apha inoba 3weeks back ukhona umtana apha endandikuxelela ngaye

maybe you have forgotten. When you were here 3 weeks back, there was another girl here, the one I was telling you about

1. ndakhumbula um um

I remember yes

2. lo mntana mos unefamily yakhe…ifemile yake irich uhlala kwalapha

this girl has her family…her is from here. It is so rich.

1. ewe

yes

2. so ngoku sise…

now we are at…

1. ngulo waweuthi ubanexhala lakhe uhamba ebusuku…ayinguye lo

it is not that one you said that you are worried about her she likes to go out at night

2. ha a…ukhona omnye…bekhe weza apha…but ugqibele kudala apha..uhleli uhamba apha esithubeni

no, there’s another…bekhe weza apha… but she is scarce at the moment…she is everywhere not at one place.

1. e e

yes

2. so ngoku njengokuba siseklinikhi sihleli apha ezitulweni ambize usister athi ‘hayi mani ndicela wena’ kufika le moto yokutsalisa amagazi ihamba ngo8 mosi la moto

so while we were sitting on clinic’s chairs she calls her and say ‘can I please see you’ because the car for blood testing had just arrived, it comes around 8 o’clock that car (it goes away around 8 o’clock that car)

1. um um

yes

2. athi yena hayi andizotsala gazi mna. Andifuni utsaliswa gazi. Qha kangoku usister athi ‘hayi ndenza into bana andifuni ibe ngathi ndiyakungxolela because kuthiwa masinicenge’

then she says no I’m not going to do blood taking. I don’t want my blood to be taken. Then the sister said ‘I don’t want it looks like I’m shouting at you, because we are told that we must convinced you politely.

1. Um

Yes

2. yiza. Nditsho kangoku kuye ngena mani Zikhona akho niks nie because nam ndizowuza ndizotsala igazi ekugqibeleni. Yena ixhala lakhe ubana. Uqhele undijaja

come . then I said to her…there’s nothing Zikhona because even me I’m oing to come for blood taking anyway, her worry is, because most of the times she judges me

1. um um

yes

2. xa mna ndibheka eklinikhi so kangoku…andazi noba usister uyam menya xa esithi makaze azotsala igazi

when I’m attending to the clinic…I know whether she thinks the sister is embarrassing her when she calls her

1. o
2. ‘hayi andizokuyenza lo nto leyo. Uyathanda ufika ujongisana nabantu wena kule klinikhi’ ntoni ntoni ntoni. Athi usisi Khuselwa ‘okay’ ndithi kuye hayi mani ngena Zikhona wabe kangoku engafuni ukutsalwa eligazi ‘hayi libuhlungu andizo yenza…andizoyenza lo nto leyo exelela usisiKhuselwa…’uyabona ke..ukuba uzothi uhleli nje wenza le nto…mna ndizokuyeka…because wawugula uleli nge…uyathetha kangoku because xa enomsindo mos la sister uyathetha

‘no I’m not going to do that. You like to make people here a centre of attraction in this clinic’ and so on. Then sisi Khuselwa said ‘okay’ then I said as well no Zikhona go in, then she made it clear that she does’t want her blood to be taken ‘no it is painfull to pull blood out’ (can’t think of the term used to take blood)…I’m not going to that thing’ telling usisi Khuselwa… ‘you see…if you continue doing this, I’ll just leave you alone…because you were very sick before…’ this sister speaks so much when she is angry

1. Um

yes

2. Because lomntwana uyaphendula apha naye phakathi kwabantu. Athi kangoku omnye hayi mani sister kutheni ungambizeli ecaleni apha ekoneni. Agqwashule lamntana ‘andizoyenza lonto leyo’…ntoni ntoni ntoni

Even this girl is exchanging word while she is standing among the people. Then the other girl asked the sister why don’t you call her aside and speak with her…then she went on blazing again that girl ‘I’m not going to that’ and so on.

1. Um

yes

2. Baphuma athi ubuya kwakhe abuze kum ukubana ‘sowucaliwe wena igazi’ ndithi ewe ndigqityiwe utsalwa igazi mna ‘hee sowugqityiwe akhonto ibuhlungu pha’ ndithi ke mnake tsala igazi mfondini akho niks nie phayana hayi wangena watsalwa

Then they went out then she came back, she asked ‘they have taken your blood?’ then I said I’m already done with blood taking in awe ‘you are already done. There’s nothing painful.’ Then I said just go in for blood taking there’s nothing painful. She went in and her blood was taken.

1. Um

yes

2. Kwemkwa neligazi lakhe…abuye azothi kum ‘ngoku iipilisi zakho uzinikiwe’ ndithi andizanga uzokuthatha iipilisi kaloku mna qha bendizotsala igazi

They went away with her blood…then she came back to say to me ‘then they have given you your pills’ then I said to her I wasn’t here to take my treatment I was here for blood taking.

1. Um

Yes

2. Iipilisi ndise nazo endlini so ngoku njengokuba sibuya nje uhamba endisearcher ukbana ‘nawe utya ezipilisi ndizityayo’

I still have the pills at home. On our way here at home she is interoating me ‘even you do you take these pills I’m taking’

1. Um

yes

2. Ndithi kuye hayi ndiyazitya Zikhona but andibe ndikhwaza ngalonto mna

Then I said yes I’m taking the same one Zikhona but I’m not a loud speaker about that

1. Um

Yes

2. Nawe..ubangaba ubungomnye umntana uthi keyena ke ‘hayi mna, into endityhafisayo ekutyeni ezipilisi sisi ndingulo ungenadawo yokuhlala ndihamba ndibhampula’… ndithi kuye ingxaki yakho…naankuya umam’ akho umama wakho kudala ekubiza phana usister kamam wakho awufuni uhlala naye kuba ufuna ukwenza lento uyi…ufuna ukwenzwa nguwe

Even you…if you were another child then she says ‘no the thing that discourages me in taking these pills sisi I’m this one who don’t have a place.’… then I reminded her saying, there’s your mother. Your mother is been calling you. You don’t want to stay with the sister of your mother because you want to do whatever as you please yourself…only the thing you want to do.

1. Um

yes

2. Weza ke nyani…ngoku xandizofika apha endlini…athi uzogqitha nam aze apha endlini. Ndifike apha endlini ndimphe. Sitye, caba unazo…nazi ezipilisi apha kuye empokothweni. Kuba njengokuba sibuya nje ubalekile waya kuleboyfriend yakhe…impahla yakhe mos xa efikile ihlala pha

Truly she came…then when I was close home…she said she will accompany to my home. When I arrived at home I gave her. Then we ate, seemingly these pills are inside of her pocket, because as we were approaching home she ran to her boyfriend. When she is around her belonging stay there…

1. O
2. Wathatha iipilisi zakhe wafaka empokothweni

She took her pills and put them inside her pocket

1. Akahlali apha?

Doen’t she stays here?

2. Akahlali apha

She doesn’t stay here

1. O
2. Wayothatha…apho afike khona ufika abe nomntu ahlalisane

She went to took her belongings…where ever she stays she gets someone to stay with (man)

1. O
2. So kangoku umama khe zezanto angazifuniyo kuwe

So those are the things her mother doesn’t like from her

1. Um um

yes

2. Ba uthi uzazi ukubana wawugula njani but ngoku waufuni uphinda umamele kwam mna

Because her mother always reminds her you know how you were ill and now you don’t even want to listen from me

1. Um

yes

2. Uyaphinda ubaleka esitratweni. Wafika apha endlini ukugqiba kwethu wathi. Mandimphe amanzi ndabona ekhupha iipilisi, uyasela uyatya. Ndithi kemna uyabona kangoku Akhona akukhonto ibitheni ‘hayi bhere ndihlale nani mani nina noZethu because nina nithetha gca umntu niyakwazi umcebisa, nomakazi ke noba naye uyathetha ‘hayi Zikhona xa ungena nto, nathi asithi ukba siba nento okokoko yiza uzofumana loo nto uyifumanyo. Okba usele iipilisi zakho

Again you want to be in the streets. When she came here at home she said, I must give her some water, and then I saw her taking it some pills, she drank and ate. Then I say you see Akhona there’s no stress ‘yes it’s better I stay with you and Zethu because both of you say the truth the way it is, you know exactly how to advise someone, even our aunts said something ‘Zikhona when you have nothing, not that we always have something but you can come you’ll get what we are having. So that you gave something before you take the pills.

1. Um u…so ucinga mhlawumbi yena.akazithathi kuba engana ndawo ncam yokuhlala okanye engena..suba esonqena ukuba uzothatha engakhange atye

Yes…so do you thenk maybe her, she doesn’t take them all the time because of not having a place or because of…or it’s because of having no food.

2. Ewe isiszatho sokuba la mntana anagazithathi eza pilisi yilento yokubana akana ndawo yokuhlala

Yes the major reason of that girl not taking her medication it is this thing of not having a place to stay

1. Um um

Yes

2. Engenandawo yokuhlala nje uyathukwa ngumakazi wakhe usister kamama wakhe

She don’t have a place to stay because her aunt always swear at her, the sister of her mother

1. Umama wakhe yena uphi?

Where is her mother?

2. Umama wakhe wasweleka because yena lomntwna egula nje wayifumana through kumama wakhe

Her mother passed away and that’s the reason even this child is sick she got it through her mother

1. O
2. So uhleli nje bayamenza lanto lamntana because uba ufikile apha ufika ahlale uzothi asahleli azobizwa ngulamakzai wakhe athi ‘uhleli apha’ ntoni ntoni ntoni uyathukisa uyathuka

So they treat her in that way all the time because she visits me, that aunt of hers will come to call and say ‘you are here still!’ and other swear words

1. Um um

Yes

2. Uyathukisa aphele etyhafa kangoku la mntana. Athi bemfuna ukba makaye ekliniki amke negam lakhe uzovela abuye kulandawo sekuthiwa akaphilanga uyagula njalo njalo

She always says swear words and ultimately the girl get discouraged. The aunt says I wanted you to go to the clinic and then the girl disappears. When she comes back, we always learn that she is so sick…

1. Um um o okay. Hayi! Ke sigqibile kangoku

Yes okay. Then we are done at this time

2. Sigqibile

We are done

1. Yeyokugqibela le?

This is the last one?

2. E e yeyokugqibela ziyi5

Yes is the last one they are 5

1. Hayi masibulele kakhulu ke sisi. Siyayibulela kakhulu impathiswano yenu..yethu. nokungatyhafi kwakho. Ukuba uqhubeke usiphathele ezifoto. Ndinethemba le nto ba. Ekubeni sizoqhubeka sisebenzisana nawe sifundisana ngeba…ukuba uyakhumbula kuqala ngoku ndandisiza apha ndandisiza ndithi…ikakhulu kakakhulu kukufundisana intoba ithi kangoku xasithe sazifumana ezingxelo sikwazi ukufundisa nabanye abantu. So into oyifundile nathi esiyifundileyo izoncedasana nekliniki incedisana nabahlali incedisane naye ugovernment incedisane kwanabafundi abasafuna ukufunda ngesisifo ikakhulukazi ngomama abancinci noomama abanantwana abancinci okanye abakhulelweyo. Siyabulela kakhulu kakhulu sinethemba lokuba ininzi nawe into ozoyifunda

Let us thank you sisi greatly. We are really thankful about how you have handled us without getting annoyed. To continue bringing photos for us, having a hope of that we will continue working with you educating one another…I should be…if you still remember the first time I came here I was saying…the biggest thing is to teach one another so that when we got these reports we can be able to teach others. So what you have learnt and us what we have learnt is going to benefit the clinic, the community and the government; it will also benefit the students who are still interested in learning about this disease, it will benefit a lot young mothers, mothers that have young children and those who are pregnant. We are very greatfully we have a hope that even you – you are going to learn a lot.

2. E e

Yes

1. As sisezokuba…njengokuba ndisitsho sisezojonga ke ngoku uba singafundisa njani uqhubeka nayo yonke inxaso ebantwini abanengxaki yokuthatha iipilisi

As we are going… as I’m saying we will see on how we are going to elighten each other to continue supporting the people of are having this problem of taking pills

2. Um um

Yes

1. Enkosi kakhulu

Thanks very much

2. Enkosi

thanks

1. Akhonto ufuna ukuyithetha wena?

There’s nothing do you want to say?

2. Hayi akhonto qha nam ndizobulela bekumnandi ukuqhubekeka nani. Ndizophinda ndiqubekek ukubana ikhona esekhana ichance akukho nto ibitheni

There nothing but I will just pass my gratitude as well it was very enjoyable to work with. I’m looking forward of working with you again if we get a chance again.

1. Enkosi kakhulu

Thenk you very much
