# Supplementary material for: “If I don’t take my treatment, I will die and who will take care of my child?”: An investigation into an inclusive community-led approach to addressing the barriers to HIV treatment adherence by postpartum women living with HIV
Source: PLoS One. 2023 Apr 20;18(4):e0271294. doi: 10.1371/journal.pone.0271294 (PMC10118130; doi:10.1371/journal.pone.0271294)
Supplement: S5 File — (ZIP) [file pone.0271294.s005.zip › 34-18 3rd.doc]

4^th^ rec

V1: Good Afternoon today is the 16^th^ of May2018 and im with participant number 3491. She hasn’t been able to take a lot of pictures but she’s got some few photo to tell her story thank you. You can start, ungaqala ke sisi usixelele

V1: Good Afternoon today is the 16^th^ of May2018 and im with participant number 3491. She hasn’t been able to take a lot of pictures but she’s got some few photo to tell her story thank you. You can start now sisi

V2: lena yifoto yasesibhedlele lento yeyesibhedlele infact ngeba ndithi iclinic, into eyenzekayo kwakunzima kakhulu ngexesha ndandidilishana neclinic. Ubunzima obabulapha eyonanto yayindenzela istress ngexesha lokuya eclinic ndandiye ndicinge ey ndizakudibana nabantu endibaziyo especially ndaye ndathi ndakuqala ukungena ezipilisini ndadecider ukuba mandikhe ndisuke apha kulendawo ndaziwa kuyo

V2: this a photo of the hospital maybe I should say it a clinic, you know it was hard when I was dealing with this clinic. The hardship that was there the stress that I had was that I was going to the clinic and people that know me would see me there. So when I started taking treatment I decided to take them away from here in an area where no one knows me.

V1: oh wemka

V1: oh so you left

V2: ndemka ndaya kwesinye isibhedlele endingaziwayo total kuso,

V2: I left and went to a hospital that no one knew me

V1: oh zezinye zecallenges owadibana nazo kwakufuneka uhambe apha kulendawo uhlala kuyo

V1: oh those are the challenges that you had, you had to leave your area

V2: ewe ndikhwele ngemali ndiyoqabela, kuba ndandiqonda ndinentloni zentobana into eskakhulu ndandicinga ukuba lihlazo ukuqala kwam ukuba nesisifo ndinento ethi inoba kuzakuthiwa ndingumntu ophandlapha khange aziphathe kakuhle. Although ndandizazi ukuba ndayifumana njani, andizazi ndayifumana phi na, but ndandinento ethi mna ndaye andaziphatha kakuhle. Ndinentloni

V2: yes had to take a taxi to get there because I was ashamed of having the virus, although I knew how I got it but I didn’t know where I got it. I felt I didn’t take care of myself and I was ashamed

V1: so yenze kengoku ungathathi kulendawo uhlala kuyo

V1: so that made you not take your treatment where you are staying.

V2: ewe, ndemka kengoku ndayoyithatha kwesinye isibhedlele.

V2: yes I left and took it from another place.

V1: so wathini ke wemka kwalapha kulendawo wohlala kwenye indawo?

V1: so what happened did you go to stay in that area?

V2: zange ndimke ndandisimka ekuseni ndiyokuthatha

V2: no I didn’t leave I would go there in the morning

V1: so uhambe uye kwenye indawo

V1: so you would go some where else

V2: kwenyidolophu, so yeyonanto yayindinika uxanduva ndaphantsa ndaziyeka kuba sometimes yayifika idate ndingenayo imali yokukhwela but kwanyenzeleka into yokuba mandizame ndiphumelele ndiye ndiyokuzithatha. Nyani kwahleleka ixesha elide ndade ndaba ndiyaqhelana kengoku nokuzithatha kulendawo. Now kengoku ndaqhubekeka ndazithatha apho ndaqhela ukuzithatha ipilisi.

V2: in another town, so that was what was giving hard time because I didn’t have money for taxi when my date comes but I had to make means to get here to get my treatment. For a long time I took it there until I got used to taking it here, now im taking it here.

V1: so esisibhedlele sikukhumbuze into yokuba unohambo olude oluhambileyo.

V1: so this hospital reminds of the long way you have come?

V2: bendinohambo olude ukuya kwesibhedlele ibiyilonto leyo xa ndibona esisbhedlele. Ndaba nengxaki kakhulu nokuthatha apha kuba ndandibanento ethi ey xa ndingena apha ingathi ndibhaliwe into yokubana ndizokuthatha ezipilisi. Ndizive ndingakhululekanga nyani ndaphantse ndaziyeka ipilisi but zange ndiziyeke ngenxa yalonto. End of story ke ngehospital.

V2: I had o long trip going to this hospital. I had a problem taking it here because I used to feel like its written on me that I'm here for what. I almost stopped taking the pills but because of that I didn’t. End of the story about the hospital

V1: ok siya kufoto namber2

V1: ok we are going to photo number2

V2: foto namba 2 zipilisi ezi

V2: photo number2 its my pills

V1: ok

V2: zipilisi zam ezi ziloluhlobo, ipilisi zam ngelixesha sendide ndaqina isibindi sentoba mandize kuleclinic yam ndandiba nestress because usister wayengandenzeli kakuhle wayenoqhekre eziveza umfumanise ukuba yena akanayo lanto ukuba uyokuzithatha uzakuzithi dleke akakhathali noba udibene nomntu epasejini okanye. Uyabonakala mos umntu xa esuka eDispesery kweyam iklinik uyavela apha ebantwini and engenayo nalangxaki yalangxolo lakrwalakaxa yeyonanto yayindenza ndiphambane walakralakwaxa because yaziwa nasisibhanxa lakrwakaxa zezipilisi. Ndandiba nestress nalapha xa ndizakuya, ndive ukuba akukhomnandi kangokuba bade bandiUndertander ndabacela ukuba mabagalele phi kwiplastic yepilisi bazikhuphele kulamakhohlombe ezabhotile zazo noba ndiyahamba endleleni ngelaxesha ndandivela ndigoduke straight ndiye eclinic sele ndiphethe iplastic ndivele ndizikhuphele eplastikini ndizikhethe endlini yayindinika ingxaki.

V2: its my pills, I used to be stressed because the sister used to just hold them and not hide them from everyone else. You see the dispensary is in front of people so she would pass with our pills just carelessly. I used to be stressed to go to the clinic but in the end they understood me and I put my pills in a plastic bag instead of the container.

V1: Bendingayiqondi ke le into, azikho ezinye ipilisi ezibakweza container?

V1: I didn’t understand this, are ther any other pills that are in the same container?

V2: Zibakwicontainer

V2: they are in a container

V1: senditsho ezinye ipilisi ezingezizo ezi zeARV’S? zizo zodwa ezibaphayana?

V1: I mean other than ARV’s?

V2: zizo ezibaphayana nganeno kwezapilisi kuthwa zikhusela iTB. Ikhona ndiyilibele ukuba kuthiwa zintoni zezokukhusela iTBsiyazinikwa ke thina bantu baHIV.

V2: there are those I forgot what they are called but there are for TB prevention we get them as well.

V1: but ezenza langxolo

V1: but they don’t make that noise

V2: zeziya zethu

V2: its only our

V1: ndibuza nje ukhona omnye bendikhe ndathetha naye wabuthetha lonto ukuba abantwana bakhe babiza mama sowuthathile ikrolokoqo wako

V1: im asking because there is another lady that I spoke to that had the same problem of the noise, her children call her pills Krolokoqo

V2: nantsoke

V2: there you go

V1: so indikhumbuza lonto lento uyithethayo.

V1: so that reminds of that

V2: ndaye ndimcaphukela ke lakrolokoqo yeyonanto yayindenza ndibewrongo, so ndaend up bendiUnderstander kangongokuba nale isister idla ngokundinceda ndidlangokuyincoma ndithi uyabona uyacaca ukuba usengumntana uyandiunderstander. Athi ken aye xa usister xa ethetha hayi mntanasekhay wonke umntu akaphila qha asikazazi ngokwempilo zethu iright xa ungakholwanga ngempilo yakho ngohlobo ekwenziwa ngayo uze ngaphambili. Senditsho ukuba ke uyayazi uyazikhuphela

V2: I hate the noise that is made by the pills it used to make me wrong. So they ended up undertand me even the sister that used to help me underatood. She would say everyone is sick but some don’t know their status yet and you are fine to talk when you don’t feel fine about what concern you health.

V1: so istaff saseclinic sinayo intoyokuba sikhawulelane nani,

V1: so the clinic staff assist you guys

V2: kakhulu

V2: to much

V1: so kulula ukuyochaza ingxaki yakho?

V1: so its easy to chat about your problem?

V2: ewe and abayithi pahaha ingxaki yakho, senditsho ke bayandimamela nalapha. Bazame ukukhuphela qho xandifika nale inkulu ithi xa isithi ndithi hayi sister ungandenzeli ingxolo mna aphendleleni ube undijongisa ngabantu uyayazi avele akhuphele andihleke akhuophele. Ndiminshiba kukangako ndendlela yepilisi.

V2: yes and they don’t talk about other people’s problems I mean the understand me. They put them in a plastic bag even the sister incharge knows.

V1: Enkosi kakhulu yiveki yokugqibela,le sizakuyiqala siEcxited nathi ukuyiqala lephase, enkosi kakhulu otherwise akukabi nangxaki ngecamera usayisebenzisa nje kakuhle.

V1: thank you so much this is the last week, the one that we are going to start we are excited To start this phase. Thank you so much otherwise you don’t have a problem with the camera you are still using it fine?

V2: ndisayisebenzisa kakuhle

V2: im using it fine

V1: ok enkosi kakhulu siyabulela.

V1: thank you so much we are greatful
