# Supplementary material for: “If I don’t take my treatment, I will die and who will take care of my child?”: An investigation into an inclusive community-led approach to addressing the barriers to HIV treatment adherence by postpartum women living with HIV
Source: PLoS One. 2023 Apr 20;18(4):e0271294. doi: 10.1371/journal.pone.0271294 (PMC10118130; doi:10.1371/journal.pone.0271294)
Supplement: Attachment — Submitted filename: Response to Reviewers May 2022.docx [file pone.0271294.s008.docx]

Dr Melissa Sharer

Academic Editor

PLOS ONE

15^th^ May 2022

Dear Dr Sharer,

**Subject: Submission of revised paper ID [PONE-D-21-37540] [EMID: 83f081e41b56eb7) entitled “Replacing sticking plasters with sustainable change: An investigation in an inclusive, community-led approach to addressing the barriers to HIV treatment adherence by people living with HIV”**

Thank you for your email dated 1^st^ April 2022 enclosing the reviewers’ comments. We appreciate each reviewer’s careful and thorough reading of this manuscript and for their useful comments and constructive suggestions. As requested our responses are given in a point-by-point manner below and we have attached both an unmarked and marked copy of the revised manuscript.

We hope the revised version is now suitable for publication and look forward to hearing from you in due course.

Sincerely,

Katy Pepper

**Responses to review: Reviewer #1**

**General comments:**

This paper addresses an important topic: the intersection of HIV adherence and stigma among a highly vulnerable population, namely, postpartum women. The study thus has very high public health significance. Additionally, the use of participatory action research (PAR) and the active, seemingly equitable way study participants were involved in the research is important and offers valuable insights into different, less hierarchical methods of knowledge creation. These are key strengths that make me excited about the paper. Despite this, however, there are many limitations with the current version of the paper, which must be attended to before the paper is suitable for publication in a high-impact journal like PLOS ONE.

Thank you for your remarks and for noting the high public health significance of the study. I am particularly encouraged by your comments on PAR and its importance in providing a less hierarchical method of research. I am pleased that I got across the core involvement or partnership of the women in the study and their extremely valuable insights.

I note and respect the need for revisions, and I believe I have been able to transform the paper into a paper for PLOS ONE. I am very grateful for the time you have given to assist me in making those changes.

| **Suggestion, Question, or Comment** | **Authors’ Response** | **Change in the Manuscript** |
| --- | --- | --- |
| Title: I do not understand the first part of the title "Replacing sticking plastics with sustainable change". What does this mean? I do not see the relevance to the issues discussed in the paper. If it means something important to the study community then this should be explained somewhere in the paper Additionally, I think that the authors should refer explicitly to "postpartum women living with HIV" rather than simply to "people living with HIV" in the title. | The paper title was entitled ‘Replacing sticking plasters with sustainable change”. This was to point the reader towards the inadequacy and temporary nature of South African public health facility responses in managing non-adherence. The paper aimed to provide a more tangible approach, that was owned and led by the PLHIV that brought about a sustainable change in care for those living with HIV.  However, I understand that this might be a bit confusing. I have proposed an alternative title. It is a quote from one of the co-researchers and reflects the dilemma that postpartum women feel about their adherence to ARVs. | I have changed the title of the manuscript to:  “If I don’t take my treatment, I will die…who will look after my child?” An investigation into an inclusive community-led approach to addressing the barriers to HIV treatment adherence by postpartum women living with HIV |
| Methods: The authors should provide more information on the recruitment of the 10 women who participated in the study. How many women were eligible and how were these 10 women subsequently selected? Does one assume that there were more than 10 women who fit the inclusion criteria? | More women met the criteria for the study. I have rewritten the section to include more details about the process by which they were selected. | The section has been re-written and is placed under the heading “Study participants’ selection and roles”  *The study was made up of two different groups of collaborators or members:*  *The women co-researchers were all isi-Xhosa-speaking and had attended one of the 4 clinics in the Ubunye programme area at least once for antenatal care. The women were selected based on 3 criteria: first, they were living with HIV; second, they had given birth in the previous 3 years; and third, they had been diagnosed and initiated into ARV treatment either during their pregnancy or at the birth of their infant. A month was set aside for recruitment and women meeting these criteria (based on records held at the clinic) were informed about the study by the local clinic staff. Of the 37 women who met the criteria ten women were randomly selected and all of them agreed to participate in the study. One participant left the study after the orientation and training and another participant was randomly chosen to replace her and she agreed to participate.* |
| Additionally, in Table 1 can the authors include the years that study participants had been living with HIV or the year they first learned their HIV status. This is an important variable, given women's concerns about infecting their children which emerges in the findings section. | The study criteria selected those that had been officially diagnosed with HIV during their pregnancy. This does not mean that they did not know their HIV status before this time. Some of them did likely suspect, however, that their official diagnosis date was during their pregnancy (at antenatal care). Since this is part of the criteria, I have not added it to Table 1. | The following text was added under the heading Study participant’s selection and roles  The study was made up of two different groups of collaborators or members:  The women co-researchers were all isi-Xhosa-speaking and had attended one of the 4 clinics in the Ubunye programme area at least once for antenatal care. The women were selected based on 3 criteria: first, they were living with HIV; second, they had given birth in the previous 3 years; and third, they had been diagnosed and initiated ARV treatment during their pregnancy. |
| Table 2 can also be incorporated into Table 1 and does not need to stand alone. Alternatively, the Ubunye Foundation team's socio-demographics can simply be described in narrative form, not via a Table. Given that the study is located in South Africa, I believe that the race of the Ubunye researchers should be stated as this could influence the interaction between the co-researchers and Ubunye researchers. | I have changed the Ubunye Foundation’s socio-demographics into a narrative form and included their racial group and a bit more of their background. | I have taken out Table 2, re-written the section and have placed the following text underneath Table 1:  *The other group of collaborators were selected because of their work on the health programme at the Ubunye Foundation. Only one of these staff members is living in the study area. She is an isi-Xhosa woman, born and still living in the area. She is a trained* Community *Health Worker and worked as a Field Officer for the Ubunye Foundation and as a study facilitator for the study. The other two study members live in the nearby town of Makhanda. One is a Professional Nurse who worked as a Programme Coordinator for the Foundation and was a Study Facilitator. She is also an isi-Xhosa woman, born in the Eastern Cape. These two study facilitators’ roles were to set up the study in the local Ubunye Foundation programme areas, facilitate the Photovoice data collection, coordinate the data cleaning and analysis and share in the writing up of results and dissemination. The third study member, the author, is an English woman, living in South Africa for 15 years. She was employed by the Ubunye Foundation as a Programmes Manager. She was the research lead for the study, managing the overall study process. She ensured the study protocol was completed and that ethical approval was submitted, cleared and adhered to. She used Nvivo for the qualitative analysis, coordinated the sharing of key themes from the data analysis and the feedback, writing up and the dissemination of findings. The Ubunye Foundation facilitated the programme development process that emanated from the Photovoice and generated funding for the programme interventions.* |
| Findings: the authors present very interesting study findings, but the organization of these findings needs major revision. Despite the use of sub-themes, the findings sometimes read like a program report rather than findings for an academic paper. This is especially the case on pages 21-25. There seems to be a conflation of the key components of the program and study findings. The following sub-headings are not necessary and reinforce the "report"-like nature of the findings: "A request for Lelethu members to meet" (page 24), "September 2021 Review" (page 22), "VideoVoice Letters" (page 23) and "Community-led Programme" (page 21). | The section referred to has been excluded from the paper in response to Reviewer #3, (item 3) which removes the community-led programme evaluation data and focuses on the research study findings leading to programme interventions. | Section removed from the manuscript |
| Additionally, the authors focus on "two main barriers" to taking ARVS (page 14) but do not state what these are. Corrected on Page 15 I assumed that the two barriers were "anticipated stigma" and "poverty".  If this is the case, it is not clear what the data presented on page 19, under "The key differences for postpartum women" refer to. Are they still part of the barrier "poverty" or "anticipated stigma?" | I see the potential for confusion and have rewritten the section.  I see the ambiguity here too. The key difference for postpartum women is not that they alone experience poverty and anticipated stigma but that they have different perceptions of living with HIV that influence the way they will or will not take their ARVs. At the end of that section, I conclude that these different perceptions leave postpartum women in a dilemma - to either take or not take their ARVs. | The section has been re-written and is immediately under the heading Results: *Barriers to ARV adherence*  *The co-researchers identified two main barriers to taking ARVS: Anticipated stigma and poverty. At times these two barriers were insurmountable. It is important to note that none of the barriers cited relates to physical barriers such as lack of access to supplies, including clinic stock-outs, or an inability to get to the clinic.*  This section has been given a new title and the text has been re-written. It is placed in the Findings section as a third finding  ***Key differences in the perceptions of living with HIV amongst postpartum women***  *The barriers to ARV adherence of anticipated stigma and poverty could be related to many other people living with HIV. However, there were specific differences in the way postpartum women experience and perceive their lives with HIV. These differences may ultimately influence their ARV adherence.*  *First…*  *Second…*  *Third…*  *For these three reasons, these co-researchers fear not taking their ARVs, and every day, they balance that fear against the fear of taking them and being found out as having HIV.* |
| If a sub-heading only has one or two quotes or a couple of sentences, then it should be deleted because it suggests that there is insufficient data on the topic. This applies to the data presented on page 24 "A request for Lelethu members to meet" to page 25. All the subheadings used in this section have insufficient data and thus must be collapsed into one paragraph or more data must be presented in each section. | The section referred to has been excluded from the paper in response to Review #3, (item 3) which removes the community-led programme evaluation data and focuses on the research study findings leading to programme interventions. | Section removed from the manuscript |
| Is the picture shown on page 15 taken from a book or is it a drawing made by one of the women? | This is a co-researcher’s photo of a drawing from a book | No change to the manuscript |
| Figure 3 is not necessary for its current form. | Figure 3 has been removed and the text near it revised to include the information contained in the figure | This section has been re-written.  ***The HIV Stigma “hot spots” surrounding ARV consumption***  *The co-researchers described a series of HIV stigma “hot spots” or situations where they felt at high risk of other people discovering that they were HIV-positive. These hot spots surround the entire ARV pill-taking process: getting re-supplies of ARVs from the clinics; storage of the ARVs at home and even whilst transporting the ARVs from the clinic or outside the home); the packaging of the ARVs which indicate their use; and the space and time for taking the ARVs at home and outside the home. The co-researchers described the lengths they went to avoid others finding out about their status and these hot spots represented areas where there was a high risk of their status being revealed. At these times the only way to manage that high risk was to not take their ARVs.* |

| Discussion: I wonder if the discussion can go beyond summarizing the findings and linking them to other studies. A more critical discussion of the "so what" of the study findings is important. Perhaps an explicit framing of the findings around the concept of "intersectionality" by Crenshaw, might help elevate the study findings to be more critical versus purely descriptive. It appears to me that what the authors are trying to show is that postpartum women face additional challenges that centre around their role as mothers and having HIV is seen as a type of "failing" to be a good mother because of the HIV risk these mothers pose to the children. Notions of "good" mothers vs "bad" mothers are thus central to how these women experience living with HIV. Poverty interferes with these women's abilities to be good mothers.  The authors do not present any data on breastfeeding: I would assume that this would be a key concern women have as well. Did they breastfeed their children and worry that this may also have exposed the kids to potential infection? Were any of the women's children living with HIV? This data should be reported in the findings section as it helps shed important insights into "mothering" and "motherhood". | The entire Discussion section has been rewritten and is structured around the concept of “intersectionality”. Thank you so much for the idea – it has made a big difference to the whole section  The women did not bring up the issue of breastfeeding in the Photovoice data although they did talk about having protected their infants by taking their ARVs carefully during the postpartum period and therefore, the breastfeeding period. The research aimed to enable the co-researchers to come up with the issues that were important to them vis a vis their adherence or non-adherence to ARVs. We did not prompt. Therefore, although their views on breastfeeding would have been an interesting theme, we did not have data on it. | The entire Discussion has been re-written, and one part is structured around intersectionality.  *…Yet we need to unpick the findings that led to these apparent answers to evaluate the effectiveness of the study methods. The theory of intersectionality lends itself to an analysis of the predicament these women have described. Intersectionality maintains that a person’s experience is shaped by multiple identities that interconnect and interact (52). These identities are shaped through social conditioning and reinforced by institutions such as health care services. Campbell and Gibbs talk of women living with HIV as having multiple forms of stigmatised identities as vectors of HIV/AIDS, often poor and socially excluded (53). The sentiments expressed in the Photovoice data describe women as being at the intersection of HIV-related stigma and gender inequality with a specific focus on the women’s poverty….*  *…Looking at these disparities through an intersectional lens rather than as individual and independent issues, enables a view of the overlapping systems of power that affect postpartum women in this unique way. This makes it important that the interventions to address these disparities are created by the women who experience them. They alone can get to the core of how to address the intersecting issues of HIV stigma and poverty amongst people living with HIV in their communities…* |
| --- | --- | --- |

| References: There are way too many references provided: 56 to be exact. This could partly be because the data are from a doctoral thesis. Please streamline your reference section and only site those sources that you refer to directly in the paper. | The number of references has been reduced to 36 references | Only key references have been included in the References list |
| --- | --- | --- |
| Overall writing: the authors tend to use short paragraphs. Please combine paragraphs for better flow and coherence. For instance, the last paragraph on page 4 could be combined with the first and second paragraphs on page 5. As a rule of thumb, a paragraph that is only two sentences long should not be stand-alone. Please review the entire manuscript and use longer paragraphs. | Thank you, that is valuable feedback I have combined paragraphs and kept to the rules mentioned. | The paragraphs have been rationalised combining them where appropriate according to the rule mentioned by the reviewer. |

**Responses to review: Reviewer #2**

**General comments:**

Thank you for the opportunity to review this manuscript. I found this to be a singular manuscript. It is notable in its use of PAR. While the findings are not new, certainly the use of PAR with the priority population is new. I was impressed with the engagement of 10 community women, who appear to be extremely vulnerable, as co-researchers. The figures are very helpful. The manuscript held my attention and I feel that it makes a unique contribution.
With all that said, there are some minor revisions that I feel need to be made:

Many thanks for your comments. It has given me great courage to hear that you consider it to be a “singular manuscript”. The research was certainly a new departure for the communities in which we work, these 10 women were extremely brave pioneers of the approach and we have all learned a lot together. Thank you, especially for your extremely supportive and encouraging observations. Thank you also for the revisions. I hope I have done them justice.

| **Suggestion, Question, or Comment** | **Authors’ Response** | **Change in the Manuscript** |
| --- | --- | --- |
| The term "sticking plaster" was unclear to me. The term is used in the title and the conclusion, and I think that the term is similar to the American version of "putting a bandaid" on a problem However, it would be helpful to the reader if the authors could clarify the term. Also, did the use of the term "sticking plaster" come from data analysis? the co-researchers? | The paper title was entitled ‘Replacing sticking plasters with sustainable change”. This was to point the reader towards the inadequacy and temporary nature of South African public health facility responses in managing non-adherence. The paper aimed to provide a more tangible approach, that was owned and led by the PLHIV that brought about a sustainable change in care for those living with HIV.  However, I understand that this might be a bit confusing. I have proposed an alternative title which is a quote from one of the co-researchers and reflects the dilemma that postpartum women feel about their adherence to ARVs. | I have changed the title of the manuscript to:  “*If I don’t take my treatment, I will die…who will look after my child?” An investigation into an inclusive community-led approach to addressing the barriers to HIV treatment adherence by postpartum women living with HIV”*  This is a quote from one of the co-researchers and reflects the dilemma that postpartum women feel about their adherence to ARVs. |

| Some grammatical and language issues need to be addressed - for example, several sentences start with "they" and I had to re-read the sentences to better understand who/what "they" meant. I suggest revising to clarify by using nouns, rather than pronouns, to start sentences. | Many thanks for that useful observation. I have reviewed and revised the entire document and have substituted pronouns for nouns where necessary. | Changes made throughout manuscript |
| --- | --- | --- |
| There are several what appear to be one sentence paragraphs - - perhaps this is just a formatting issue. | Thank you, that is valuable feedback. It was more than a formatting issue. I have combined paragraphs. | Changes made throughout manuscript |

**Responses to review:** Reviewer #3

**General comments:**

Overall, the article is very interesting. I haven't seen nor read a similar study before, which means the novelty is good. The modification of the photovoice method, to protect the anonymity of PLWHA, was commendable from an ethical point of view, and I hope that method would be replicable for similar studies involving vulnerable populations.

Thank you for your comments and suggestions. They are very valuable. It is very encouraging to hear that you found the article interesting with its novelty status. Your comment on the modification of the photovoice method is very useful and we hope that it is, indeed, going to be a method used when we and others work with vulnerable people.

| **Suggestion, Question, or Comment** | **Authors’ Response** | **Change in the Manuscript** |
| --- | --- | --- |
| However, I suggest the writing be more concise and straightforward in the method section, with less focus on the old photovoice model and more on the modified one. | Thank you. I have reviewed and revised the Method. I have cut out the discussion of the old photovoice model and expanded the description of the way we used the adapted version. | Under Study Procedures in the Methods section, there is now a heading The Photovoice research process with a more detailed description  *The Photovoice research process*  *In preparation for the research process with the co-researchers, the lead researcher conducted a three-part training session with the Ubunye research associates. These sessions were conducted over several weeks before the start of the study and were usually 1-2 hours long. It covered three areas: aims, approach and practical considerations for the study; practical use of the cameras, taking photos, introducing and encouraging the Photovoice technique with co-researchers and arranging the 4-week study cycle; training and mentoring the staff in running the weekly debrief sessions with the co-researchers.*  *The next step was for each of the 10 women who agreed to take part in the study to participate in an orientation and training about the Photovoice study aims and processes. The training covered three sections: the use of the camera; rules for taking photographs; and orientation to their work as co-researchers Written guides in isi-Xhosa were shared for each section, and participants were encouraged to ask questions and practise with the cameras. The session on-camera use was mainly on how the camera worked and how to ensure the photos they took were stored. It also included ensuring their safety as they used the camera and how to explain their photo-taking to others without risking their HIV status. The main rule for taking photographs was ensuring all the photographs taken did not include faces or any image that could identify another person. This included their image and that of their family and friends. The session on their work as co-researchers centred on thinking through how they would take photos on the research theme “the motivators and barriers that they experience or have experienced when taking their ARVs”. They were encouraged to think about events in the past and current times when they have been taking their ARVs and the scenarios which make it easy or difficult physically or emotionally to take their ARVs.*  *For the study period, all the co-researchers were given cameras to use for over 4 weeks. At the end of each week, they met with the Ubunye staff member and went through the photos they had taken. These debrief sessions were recorded. The co-researcher was able, at any time, to remove a photo from the collection or stop the recording or not discuss a photo. The aim was for them to choose what best illustrated the issue they wanted to discuss about taking their ARVs.* |
| I also did not see a clear timeline of when the data was collected. | I have added a timeline to the study context – thank you. | This text has been added to the Study context:  *The study took research section of the Participatory Action Research was conducted in 2018-2019. The action section, the programme development and implementation are on-going.* |
| Further, I read into the result and discussion section, and I got the impression that there were at least 2 studies in this article. One using Photovoice and one using Video Letters as an evaluation of the Lelethu program (?). While the notion of inclusivity and participatory research that led to the community-led program to reduce perceived barriers to ARV adherence is admirable, this could be at least two different scientific articles with a more deep and focused discussion section. My suggestion is the author may choose to focus on one, either the one with the photovoice method or the evaluation of the Lelethu (?) program. | Thank you, this was a very insightful and extremely helpful observation and solved the main conundrum I faced with the paper. I have now taken out the community-led programme data. I have added information on the other Action done with the study on the dissemination of findings, detailed the interventions designed by the co-researchers and put a brief outcome statement for the interventions with a link to future papers. I have revised the paper throughout to focus on the photovoice research and expanded the discussion as suggested. | The entire paper has been revised to reduce the focus to just the photovoice study. Below I have put the shortened account of results from the follow-on programme as an example. This is in the newly labelled section: Action: Development of a Community-led programme.  *The effectiveness of these interventions will be evaluated by the Programme Working Group over the coming months using the PAR methodology. However, there have already been several notable outcomes from the Lelethu programme: of the 85 women aged 16-24 years in the programme 31 women have been supported to find jobs, 19 women started a business and 13 have gone on to further study. Nine Lelethu women have started work advocating against HIV and TB stigma in their local communities; one Lelethu woman was highly commended on her presentation about the Lelethu programme at a multidisciplinary seminar on Gender-Based Violence organised by the French Embassy in Cape Town in 2022.* |

**Responses to review: Reviewer #4**

**General comments:**

The topic is interesting and has the potential to inform how researchers work with people living with HIV and organizations that serve them. However, as currently written, the manuscript requires more clarity about its objectives, clear alignment of the manuscript’s content with its objectives, and better organization of multiple sections of the manuscript. The presentation of findings, the introduction, description of the methods, and results was disorganized and confusing to follow. The program development steps based on qualitative data findings were unclear/link not compellingly described. For example, it is unclear how the two important barriers to ARV adherence (anticipated stigma and poverty) were addressed in the program development.
I offer the following suggestions for the author to consider.

Many thanks for your encouragement about the topic of this paper. I am particularly grateful for the detailed suggestions you have provided for the revision of the paper. This has been a very useful learning experience for me. Thank you so much.

| **Suggestion, Question, or Comment** | **Authors’ Response** | **Change in the Manuscript** |
| --- | --- | --- |
| Study title: It is not obvious to me how the first part of the title (“replacing sticking plasters …”) reflects the content of the article. | This is a common comment to 3 of the reviewers – thank you. The paper title was entitled ‘Replacing sticking plasters with sustainable change”. This was to point the reader towards the inadequacy and temporary nature of South African public health facility responses in managing non-adherence. The paper aimed to provide a more tangible approach, that was owned and led by the PLHIV that brought about a sustainable change in care for those living with HIV.  However, I understand that this might be a bit confusing. I have proposed an alternative title which is a quote from one of the co-researchers and reflects the dilemma that postpartum women feel about their adherence to ARVs. | I have changed the title of the manuscript to:  “If I don’t take my treatment, I will die…who will look after my child?” An investigation into an inclusive community-led approach to addressing the barriers to HIV treatment adherence by postpartum women living with HIV |
| Throughout the manuscript, the author described the inclusive and community-led aspects of the project. Yet, the manuscript has one author. Please clarify the role of the co-researchers and the CBO in disseminating and writing-up study results. | Due to various health programme delays and challenges including staff changes and the movement of co-researchers out of the province, a jointly written paper for a peer-reviewed journal has not been possible. However, the co-researchers and the NGO staff have been central to the dissemination of findings. I have now included a section on the dissemination activities they have led and partnered in and an explanation for the sole authorship of this article. | This section has been added to the Results section as one of two actions that have resulted from the PAR process.  ***The Action part of Participatory Action Research: Dissemination of findings***  *In line with the Participatory Action Research methodology, the Photovoice research led to Action. The Action was in two phases, first the dissemination of findings: and second the development of the programme. The dissemination of findings aimed to advocate with policymakers, and programme managers for inclusive, participatory programmes for people living with HIV and to address HIV stigma both locally and nationally in South Africa. To address these aims, the Ubunye Foundation and co-researchers identified three conferences and a community research competition to which they successfully submitted abstracts. An abstract was accepted and presented as a poster presentation at the International AIDS Conference in Amsterdam in June 2018 by Ubunye staff and at the Durban AIDS conference in 2019 by both co-researchers and staff. Co-researchers and staff members presented at the Public Health Association of South Africa conference in 2018. The jointly developed submission to the Community Chest Impumeleo Awards won a Social Innovation Award in 2018. To address the need for advocacy for change, co-researchers and Ubunye staff presented findings at Department of Health HIV programme meetings both locally and in the district. Unfortunately, due to various health programme delays, staff challenges and the movement of co-researchers out of the Province, a jointly written paper for a peer-reviewed journal focused on promoting the programme approach has not been possible. In addition, the co-researchers did not want their names published with the article. Therefore, this article is offered as a collaborative effort of thirteen people who were all key contributors to the Photovoice research, dissemination and programme development.* |
| Introduction: Overall, the introduction reads well and provides relevant info to set up the rest of the manuscript. Organization can be improved.  Details are below. a. Page 4: Please clarify what you meant by measures. If referring to programs or interventions, please use these terms. Measures could be confused with instruments/scales etc. | Thank you, the first part of the introduction has remained the same. The latter parts have been reordered and relabelled. The term “measures” has been replaced. | The start of the Introduction section has been rewritten:  *Health care services’ interventions aimed at addressing non-adherence to antiretrovirals (ARVs) by those living with HIV in South Africa are often derived from a single source. HIV health care services pick their targets of the support they provide for adherence from the findings of health services research. Health service research frequently recommends a focus on commodity-supply changes such as ARV supply-chain improvements or using community distribution networks, dispensing machines, and telephone reminders (1-3). Some interventions have centred on differentiated care for self-help groups and adherence clubs.* |
| Page 5: Please include relevant stats, e.g., data comparing poverty levels among female-headed households by HIV status. | Recent stats for South Africa on female-headed households’ income and HIV status are not easy to find unless we extrapolate: women have the highest burden of HIV and the increasing prevalence of female-headed households. I did not include this in the text. But I did find more details on female-headed households in Stats SA and I have included them. | I have added the following to the section under Background in the Introduction:  *Poverty levels are the highest amongst female-headed households. StatsSA points to a 2.3% increase in the poverty gap to 23.2% for female-headed households in 2015, compared to less than a 1% increase to 13.6% in the poverty gap for male heads of households(21).* |
| It seems poverty and low socioeconomic status are being used interchangeably. I would acknowledge the identical use of these terms, while also noting limitations when researchers use poverty and low socioeconomic status interchangeably. | Thank you this is a very useful point. I have instead referred to poverty throughout the text. | I have changed the text to include poverty where possible. The most changes were in the text in the paragraphs under background in the Introduction section:  *Several studies reveal a strong link between poverty and poor adherence to ARVs by women living with HIV(25). The studies indicate that the effects of poverty, particularly food security, inadequate housing and unaffordable travel costs, meant women struggled to take their ARVs regularly (26). The link between poverty and food insecurity is undeniable. The most common reason for missing ARV doses is a belief of severe illness if the pills are taken on an empty stomach (27). When food supplies are extremely scarce, ARV adherence may cease entirely (28-30). The poverty experienced by women triggered by their unequal access to education and employment opportunities means they often feel compelled to stay with their families, or with a male partner, even when this may risk violence (31). A longitudinal study of South African women postpartum, found that those living in poverty were more likely to experience, and were more vulnerable to, intimate partner violence (IPV) and the accompanying psychological distress than those with more financial stability (32). This in turn increases their risk of failing to take their ARVs at* *the time of the violence and depression (33) (34).* |
| Page 6: Did the review only identify anticipated stigma as a barrier to treatment initiation? How about other stigma experiences, e.g., perceived, internalized and enacted stigmas? Also, please define anticipated HIV stigma. | The co-researchers referred to anticipated stigma as the overriding influence on them. Therefore, I have concentrated on the literature on the influence of anticipated stigma. I have defined each of the types of stigma so that anticipated stigma can be identified as the relevant type found in this study. | I have rewritten the section in the Background of the Introduction to include the definition of all stigma types:  *People living with HIV often describe HIV stigma as being the main barrier to them seeking care and remaining on treatment. This stigma is often divided into three types of stigma experience: “internalised” stigma, their assimilation of stigmatizing beliefs leading to feelings of shame and anxiety associated with being HIV-positive or on ART; “externalised” stigma based on their direct experiences; and “anticipated” stigma based on their fears of prejudice and discrimination based on HIV status(35). This experience of anticipated stigma is well documented as being a major factor in controlling the taking of ARVs by people living with HIV. In their review of research into why people newly diagnosed with HIV do not start their treatment, Ahmed et al show how those experiencing anticipated stigma felt that starting ARVs would broadcast their status, putting them at risk of stigmatised reactions from others. These reactions could be social rejection and isolation by family, current partners, peers and the community around them (35). Peltzer et al. in their longitudinal study of stigma experienced by pregnant women living with HIV found that anticipated stigma was associated with alcohol abuse, depression, decreased HIV knowledge and non-disclosure of HIV status (36). For mothers living with HIV, their fear that HIV would compromise how they are perceived by family and others often delays disclosure of their status to their children or other close family members (37-39)*  And I have repeated the definition here in the results section to underline the way the co-researchers have described the stigma they experience  *The women in this study spoke of stigma as being the single most powerful determinant of the way they manage their lives. They described the predominance of “anticipated’ stigma” defined as being based on a person’s fears of prejudice and discrimination because of the HIV status (35).* |
| Page 7, 1st sentence, 2nd para. “This study was developed to enable postpartum women to unpack and begin to address these pressures.” Please specify “these pressures.” Are they stigma and poverty? | Thank you for pointing this out. No, the study was aimed at identifying and then addressing the barriers to ARV adherence in a programme for women living with HIV in the area. I have corrected the text by rewriting the entire paragraph to tie in with the comment h below | I have rewritten this sentence at the end of the Background section to the Introduction:  *This Photovoice study was developed to enable postpartum women to not only unpack the barriers they experience in taking ARVs but to address those barriers by disseminating their experiences to others and developing a programme of interventions designed to tackle them.* |
| Page 7, 2nd para beginning 2nd sentence should be moved to the methods section.  g. Page 8 the entire page should be moved to the methods section. | Thank you. I re-ordered and rewritten sections of Methods. have moved both sections; the former to the ‘Study Context’; and the latter to the Study participants' selection and roles. | The manuscript has been reordered |
| Please acknowledge the previous studies that used photovoice methods when working with people living with HIV and situate your study given previous similar works. Please refer to Teti M, Koegler E, Conserve DF, Handler L, Bedford M. A Scoping Review of Photovoice Research Among People With HIV. J Assoc Nurses AIDS Care. 2018 Jul-Aug;29(4):504-527. DOI: 10.1016/j.jana.2018.02.010. | The section on PAR and photovoice has been revised to include setting the work within the context of previous Photovoice studies with PLHIV including the reference you have given – thank you. I have also expanded the section to include the challenges associated photovoice method and its relevance to the adaptation of the method used here. | This new heading and paragraph now begins with the Methods section  ***Participatory Action Research using the Photovoice method***  *The study described in this paper follows Participatory Action Research (PAR). PAR contains four essential elements: Participation of those who are the focus of the research, in collecting knowledge through a systematic Research process, resulting in Action that leads to Social Change (42). In essence PAR re-arranges and blurs, the roles and responsibilities of a traditional research process turning the researched into researchers. In PAR “knowledge is co-created, acted on, and learning from action is sought to bring about and sustain change” (43). The method used in this study was Photovoice. Photovoice enables people to use the photographs they take to express and share their views. It is a method that has been widely used in health settings and particularly in research on people living with HIV (PLHIV). Teti et al reviewed 22 studies using Photovoice with people living with HIV and indicated that the method* *can enable them to “inform research and practice priorities…but that more studies should be undertaken resulting in action” (44). In a review of reviews of thirty years of Photovoice use in health research, Seitz and Orsini also identified a lack of following through to action. They also found inconsistencies in the way the method has been used in these studies. A central finding was a lack of participant ownership of a project. The studies did not follow through on ensuring co-researchers collected the data, analysed the data, and published and presented findings. They also pointed to an absence of descriptions of the ethical procedures used and the value placed on co-researcher well-being(45). In this Photovoice study with postpartum women living with HIV, there has been a concentrated effort to avoid these inconsistencies and to provide the co-researchers with not only a public forum where they can describe the barriers that they face in taking ARVs but also lead to a programme to address those barriers.* |
| Methods Page 9: Please clarify if ethics approval was obtained for the entire project or the Photovoice research component only | Ethics approval was obtained for the Photovoice research. The data that was used for the programme description was Programme M&E data. That data has been removed for use in another paper as suggested by Reviewer #1. | The section has been removed from the manuscript |
| Page 9, 2nd para: Please revise that information and consent forms do not only allow recordings of discussions and photographs; more importantly, they are included to protect research participants' welfare and ensure that their participation is informed and voluntary. | Thank you for pointing out this important issue. I have revised and rewritten it. | This text has been rewritten in the Ethic Methods section rewritten:  *This research followed an explicit and detailed process aimed at ensuring both the postpartum women co-researchers and the Ubunye Foundation staff were equal collaborators in conducting this study. The inclusion of the Ubunye Foundation was specifically targeted because of its community-led approach to programme development -design, implementation and monitoring and evaluation. Therefore, the preparation for the study was focused on not only introducing but actively ensuring the co-researchers ownership of the data, outcomes and programme. that followed. In addition to this ethos of ownership, protocols for ensuring the co-researchers welfare were strictly adhered to including sessions on the study methods, co-researcher and Ubunye staff roles and responsibilities and specific attention to co-researchers' welfare throughout the study process and beyond. Formal consent forms in isi-Xhosa were explained and time was given for queries and clarification before the co-researchers chose to sign. Consent centred on the co-researchers' participation in the study is fully informed and voluntary. It also allowed for the recordings of all study discussions and photographs to be used primarily for programme design but also for dissemination to wider audiences at conferences, public meetings and peer-reviewed journals.* |
| Page 9, 3rd para: Please provide examples of how this process was done by the participants. Were they allowed to show their faces, other peoples' faces? | This section does not describe the photo-taking process and the rules around showing the co-researchers other people’s faces. But I have added a section where the preparation for the Photovoice research is described. See section below | Revised text is shown in the row below under ‘The Photovoice research process’ |
| Page 10, 2nd para: How were those women selected by the clinic staff? In addition to health conditions, were there other inclusion criteria? | I have rewritten and expanded upon the selection process for the co-researchers | This paragraph now comes below ‘Study participants selection and roles’ in the Methods section:  *The study was made up of two different groups of collaborators or members:*  *The women co-researchers were all isi-Xhosa-speaking and had attended one of the 4 clinics in the Ubunye programme area at least once for antenatal care. The women were selected based on 3 criteria: first, they were living with HIV; second, they had given birth in the previous 3 years; and third, they had been diagnosed and initiated into ARV treatment during their pregnancy. A month was set aside for recruitment and women meeting these criteria (based on records held at the clinic) were informed about the study by the local clinic staff. Of the 37 women who met the criteria, ten women were randomly selected and all of them agreed to take part in the study. One participant left the study after the orientation and training and another participant was randomly chosen to replace her and she agreed to join the study.* |
| Page 10, 2nd para: Please elaborate on the role of the women co-researchers, including any training they received related to Photovoice (e.g., topics/themes that their photos should capture, selection of pictures, confidentiality and safety, number of photos taken). How did the women co-researchers select the photos to be presented? | Thank you. This whole section on process has been rewritten and expanded to address the issues you have listed and to give a detailed account of the role, training and process used by the co-researchers. | These paragraphs have been added under the new title: ‘The Photovoice research process’ in Study procedures.  *The Photovoice research process*  *In preparation for the research process with the co-researchers, the lead researcher conducted a three-part training session with the Ubunye research associates. These sessions were conducted over several weeks before the start of the study and were usually 1-2 hours long. It covered three areas: aims, approach and practical considerations for the study; practical use of the cameras, taking photos, introducing and encouraging the photovoice technique with co-researchers and arranging the 4-week study cycle; training and mentoring the staff in running the weekly debrief sessions with the co-researchers.*  *The next step was for each of the 10 women who agreed to take part in the study to participate in an orientation and training about the Photovoice study aims and processes. The training covered three sections: the use of the camera; rules for taking photographs; and orientation to their work as co-researchers Written guides in isi-Xhosa were shared for each section, and participants were encouraged to ask questions and practise with the cameras. The session on-camera use was mainly on how the camera worked and how to ensure the photos they took were stored. It also included ensuring their safety as they used the camera and how to explain their photo-taking to others without risking their HIV status. The main rule for taking photographs was ensuring all the photographs taken did not include faces or any image that could identify another person. This included their image and that of their family and friends. The session on their work as co-researchers centred on thinking through how they would take photos on the research theme “the motivators and barriers that they experience or have experienced when taking their ARVs”. They were encouraged to think about events in the past and current times when they have been taking their ARVs and the scenarios which make it easy or difficult physically or emotionally to take their ARVs.*  *For the study period, all the co-researchers were given cameras to use for over 4 weeks. At the end of each week, they met with the Ubunye staff member and went through the photos they had taken. These debrief sessions were recorded. The co-researcher was able, at any time, to remove a photo from the collection or stop the recording or not discuss a photo. The aim was for them to choose what best illustrated the issue they wanted to discuss about taking their ARVs.* |
| Page 12: Rather than a general description of what are PAR and the Photovoice methodology, it would be more instructive to describe how these methods were used in the current study.  Page 13: Study procedures generally include all the research procedures conducted to obtain the project's goals, including sampling, design, data collection, etc. | The description of PAR and photovoice methodology has been replaced with a description of all the procedures, design and data collection etc. in the ‘Photovoice process’. See section above. | The text has been replaced with the text given above |
| “Changes to the Photovoice process” should be combined with the earlier section on PAR using the Photovoice methodology. | Given the re-ordering of the Methods section, the section ‘Changes to the photovoice process now falls below the description of the Photovoice process used. | The manuscript has been reordered |
| Page 13: “Data analysis at both Photovoice and Programme M&E” is more about data management. Please elaborate more on the qualitative analysis, including specific methods or techniques, used to analyse the data, including the role of women co-researchers and the Ubunye Foundation. | This section has been revised and rewritten to include the process used for data management and Nvivo the qualitative tool used for analysis and the roles of all the collaborators of the research. | *This paragraph comes after the photovoice process description*  ***Data analysis***  *After each debrief session the photos on the camera and the recording were encoded and saved on a hard drive. The recordings were transcribed and together with the recordings were given to external translators (Masters Language students) to translate into English. The transcriptions were reviewed by both the Ubunye staff team and the co-researchers for accuracy. This was done at further individual co-researcher visits. During these visits, the co-researchers also reviewed the work of other co-researchers and the similarities, trends and differences they identified were recorded. The photos and English transcriptions were then coded using Nvivo, a qualitative research application which traces and identifies trends in the data. Once the trends and main themes had been identified the Ubunye staff and co-researchers met again in individual meetings to review them, discuss their accuracy, and implications and agree on the final findings.* |
| j. Page 14: “Programme development” does not describe the process of using data to develop a program. Please be more specific about how the data collected were used to inform program development.  Figure 2. “Steps for programme development” should be a section on its own in the results section. A figure that illustrates the various steps is helpful, but not enough, given the importance of programme development in the entire project. I would suggest creating a section on programme development that describes in greater detail how the findings from the Photovoice activity were used to develop the program. | The programme development section has been rewritten and expanded to include an additional action – that of dissemination done by the research team together.  The programme development action section describes each of the steps involved in programme development managed via the NGO community-led governance system. | The following section has been added to the Action section as described in the suggestion above on the involvement of the co-researchers in the action.  ***Participatory Action Research - Action: Dissemination and Programme Development***  *The Action part of Participatory Action Research is an essential part of the methodology. For this PAR study, Action centred on two activities: first, dissemination of findings. Once the findings had been agreed upon by the co-researchers and Ubunye staff, abstracts were jointly developed and submitted to international and national conferences, meetings and workshops. The results section documents the success of this process and the joint presentations developed and presented by the collaborators.*  The following section has been added to the programme development description  *Second, the co-researchers and the Photovoice data designed and played a leading role in the implementation of a programme to address barriers to adherence among women living with HIV in their area. This Action followed was structured around the Ubunye Foundation’s community-led governance system, Siyakhana. Siyakhana ensures that programmes are designed and led by those who are at the core of the programme's purpose. At the grass-root level are Programme Working Groups (PWGs). PWGs are made up of up to seven community representatives (who consult with the wider community) and two Ubunye staff members. Each PWG is responsible for designing, implementing, monitoring, adapting and revising the programme as required. The co-researchers contributed directly to the work of the Programme Working Group (PWG) and led the programme design via 3 steps:*  *Step 1: Co-researchers agreed on the key findings of the study and identified the areas for interventions. This was done during the Photovoice data analysis discussion process outlined above.*  *Step 2: The PWG (which included co-researchers) met to review the intervention areas and design the specific programme activities, targets and expected outcomes.*  *Step 3: Ubunye sought funding for the programme and with the PWG identified the local resources that were to be mobilised for the programme.*  *The outcomes of this process are reported in the results section below.* |

| Page 19: “The key differences for post-partum women.” Please clarify this section’s purpose. How are the results described here different from the earlier findings of poverty and anticipated stigma? | Thank you, this was raised by another reviewer and adjusted. | This section has been given a new title and the text has been re-written. It is placed in the Findings section under the section on Poverty.  ***Key differences in the perceptions of living with HIV amongst postpartum women***  *The barriers to ARV adherence of anticipated stigma and poverty could be related to many other people living with HIV. However, there were specific differences in the way postpartum women experience and perceive their lives with HIV. These differences may ultimately influence their ARV adherence.*  *First…*  *Second…*  *Third…*  *For these three reasons, these co-researchers fear not taking their ARVs, and every day, they balance that fear against the fear of taking them and being found out as having HIV* |
| --- | --- | --- |
| Page 20: “The impact of health facility responses” The title is misleading, as the section does not describe any impact that could be attributed to health facility responses. | This heading has been removed | The text has been removed |
| Page 21: In the preceding page, the author spoke about program development informed by study findings. But here, the subsection is titled community-led program: monitoring and evaluation. Before discussing the M&E, please include your findings supporting the development of the program that was implemented and evaluated. Again, please discuss how the qualitative research component of the study informed the program's development. | The section on the programme development findings including the methodology used, the findings and discussion of the community-led programme has been taken out. This will form the basis of a further article.  I have addressed the way the research findings have informed the programme development by outlining the specific interventions that are linked to each finding under the guidance of the co-researchers. | This section has been expanded and comes under the findings of the dissemination Action.  ***Action: Development of a Community-led Programme***  *The other Action emanating from the Photovoice research was the co-researchers' leadership in designing and implementing a programme to address barriers to adherence in their local area. As described above this was achieved via co-researchers’ leadership in the Programme Working Group through Siyakhana, Ubunye’s community-led governance structure. In the 1^st^ and 2^nd^ steps of the process co-researchers with Ubunye staff identified, designed and implemented a set of programme activities designed around the findings of the Photovoice research. Calling the programme, Lelethu (isi-Xhosa for “it's ours”). five interrelated programme interventions are being implemented:*   (1) *To address individual experiences of stigma and social and psychological barriers which constrain their adherence to ARVs, Community Health Workers (government employees attached to clinics) and Ubunye staff worked with women living with HIV to create and follow Life Plans. These Life Plans provide a roadmap to addressing specific difficulties, particularly around family relations, disclosing their HIV status, and accessing clinic care. These Life Plans also detail the steps to women’s economic empowerment in (3).* (2) *To address the anticipated stigma that prevents women from accessing health services or taking their pills in public, the Ubunye Foundation supported women who were open to advocating to address stigma in local schools, health facilities and community organisations.* (3) *To address the need for economic empowerment the Ubunye Foundation provides Life Skills training and mentoring which includes support for business development, job searches and applications and seeking and applying for further training and education related to obtaining their ideal jobs.* (4) *To provide peer support whilst respecting the need for confidentiality and concerns around HIV status disclosure, WhatsApp groups were formed with a distribution of data (and in some cases mobile phones) to enable women living with HIV across the area to share their thoughts, obtain accurate information on HIV, treatment and care join together on advocacy initiatives and provide details of job, training and education opportunities available.*   *The effectiveness of these interventions will be evaluated by the Programme Working Group over the coming months using the PAR methodology. However, there have already been several notable outcomes from the Lelethu programme: of the 85 women aged 16-24 years in the programme 31 women have been supported to find jobs, 19 women started a business and 13 have gone on to further study. Nine Lelethu women have started work advocating against HIV and TB stigma in their local communities; one Lelethu woman was highly commended on her presentation about the Lelethu programme at a multidisciplinary seminar on Gender-Based Violence organised by the French Embassy in Cape Town in 2022.* |
| Page 22: “September 2021 Review” This section does not fit here. The results section could also be better organized and clearer.  Page 23: “VideoVoice Letters.” What were the VideoVoice letters and their objective(s)? How were they originally used in the study? This appears to be the first time these letters were mentioned. Please include a description of VideoVoice letters in your methods.  Pages 23–25: “Anticipated stigma” This entire subsection on managing anticipated stigma is confusing, and I am not sure what's the purpose for including a separate subsection on "managing anticipated stigma." | All this text has been removed and will be used for a follow-on article | The entire section has been removed |
